# Faecal metaproteomics analysis reveals a high cardiovascular risk profile across healthy individuals and heart failure patients

**DOI:** 10.1080/19490976.2024.2441356

**Published:** 2024-12-22

**Authors:** Chaoran Yang, Leticia Camargo Tavares, Han-Chung Lee, Joel R. Steele, Rosilene V. Ribeiro, Anna L. Beale, Stephanie Yiallourou, Melinda J. Carrington, David M. Kaye, Geoffrey A. Head, Ralf B. Schittenhelm, Francine Z. Marques

**Affiliations:** a Hypertension Research Laboratory, School of Biological Sciences, Faculty of Science, Monash, Clayton, Australia; b Monash Proteomics & Metabolomics Platform, Monash Biomedicine Discovery Institute & Department of Biochemistry and Molecular Biology, Monash University, Melbourne, Australia; c Charles Perkins Centre, University of Sydney, Sydney, Australia; d Heart Failure Research Laboratory, Baker Heart and Diabetes Institute, Melbourne, Australia; e Department of Cardiology, Alfred Hospital, Melbourne, Australia; f Preclinical Disease and Prevention Unit, Baker Heart and Diabetes Institute, Melbourne, Australia; g School of Translational Medicine, Faculty of Medicine Nursing and Health Sciences, Monash University, Melbourne, Australia; h Neuropharmacology Laboratory, Baker Heart and Diabetes Institute, Melbourne, Australia; i Department of Pharmacology, Faculty of Medicine Nursing and Health Sciences, Monash University, Melbourne, Australia; j Victorian Heart Institute, Monash University, Clayton, Australia

**Keywords:** Metaproteome, disease risk, short-chain fatty acids, machine learning

## Abstract

The gut microbiota is a crucial link between diet and cardiovascular disease (CVD). Using fecal metaproteomics, a method that concurrently captures human gut and microbiome proteins, we determined the crosstalk between gut microbiome, diet, gut health, and CVD. Traditional CVD risk factors (age, BMI, sex, blood pressure) explained < 10% of the proteome variance. However, unsupervised human protein-based clustering analysis revealed two distinct CVD risk clusters (low-risk and high-risk) with different blood pressure (by 9 mmHg) and sex-dependent dietary potassium and fiber intake. In the human proteome, the low-risk group had lower angiotensin-converting enzymes, inflammatory proteins associated with neutrophil extracellular trap formation and auto-immune diseases. In the microbial proteome, the low-risk group had higher expression of phosphate acetyltransferase that produces SCFAs, particularly in fiber-fermenting bacteria. This model identified severity across phenotypes in heart failure patients and long-term risk of cardiovascular events in a large population-based cohort. These findings underscore multifactorial gut-to-host mechanisms that may underlie risk factors for CVD.

## GRAPHICAL ABSTRACT

**Figure:**
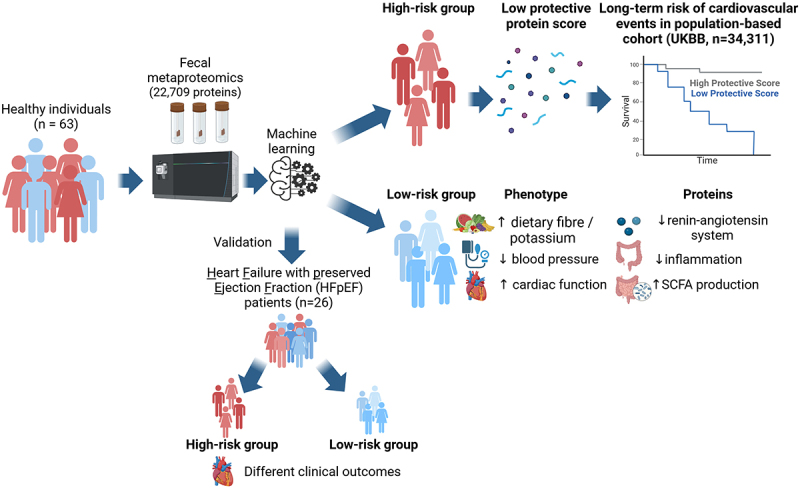

## Introduction

According to the latest Global Burden of Disease Study, both high blood pressure (BP) and dietary risks were among the leading global risk factors for death, as they are both recognized as risk factors for the development of cardiovascular diseases (CVDs).^1,2^ The relationship between BP and diet is well-established,^3^ with dietary changes listed as first-line therapy for hypertension treatment in international guidelines.^4^ The Dietary Approaches to Stop Hypertension (DASH) and Mediterranean diets are examples of how diet can be successfully manipulated to reduce BP.^4^ These diets are characterized by a high intake of potassium- and fiber-rich foods, such as fruits, vegetables, legumes, whole grains and nuts, and a low intake of red meat, saturated fat, refined sugar, processed foods, and sodium.

Mounting evidence in the past decade has underscored the pivotal role of gut dysbiosis (i.e., changes to the gut microbiome and gut barrier) in the development of hypertension^5^ and CVDs.^6,7^ Unhealthy diets directly impact human health through metabolic^8^ and gene expression changes^9^ and indirectly affect host health, including BP and cardiovascular health, by interacting with the gut microbiome and responding to downstream signaling.^10,11^ However, the intricate mechanisms underlying the host and gut microbiome interplay in CVD development remain largely unknown.

Through the assessment of microbial DNA using amplicon (e.g., 16S) or shotgun sequencing, metagenomic analyses have revealed changes in gut microbiome taxonomy associated with CVDs.^5–7^ Functional profiling and differential abundance analyses of gene families can indicate shifts in gut microbiome functions. However, metagenomic analyses are limited to estimating the presence of microbial genes, which may differ from the actual protein levels expressed by the microbial communities. Moreover, metagenome analyses cannot elucidate interactions between the host and gut microbiome. This is due to their inability to provide insights into human protein expression in the gut lumen. Thus, functional omics, such as fecal metaproteomics, have been suggested as an alternative technique to address the limitations of metagenomic studies. Fecal metaproteomics is an emerging technique capable of capturing all types of proteins in a fecal sample, including human, microbial, and food proteins,^12^ along with the biomass of different types of gut microbiome.^13^ This makes fecal metaproteomics suitable for elucidating the functional crosstalk between the host and gut microbiome, holding enormous potential. However, it remains to be explored in most disease settings, including in cardiovascular research.

In this study, we aimed to understand the functional crosstalk between the host and gut microbiome to CVD risk. We hypothesized that differences in human and microbial fecal proteins would be observed in healthy participants at higher risk of CVD and that these would influence CVD outcomes. We performed metaproteome analysis on fecal samples from healthy individuals whose BP was measured by ambulatory BP monitoring (ABPM) and patients with heart failure with preserved ejection fraction (HFpEF), diagnosed with cardiac catheterization. We further employed machine learning to establish connections between the expression profile of human proteins in the gut lumen, the composition and expression profile of gut microbial proteins, and cardiovascular risk factors. We then assessed these proteins in a subset of the UK Biobank cohort, where proteomics data is available, to validate that these proteins increased the long-term risk of cardiovascular events. Our results suggest that cardiovascular risk factors have a combined impact on the metaproteome, and that the resulting human and microbial protein changes may drive some mechanisms behind the development of CVD.

## Materials and methods

### Study population

**Figure 1.**
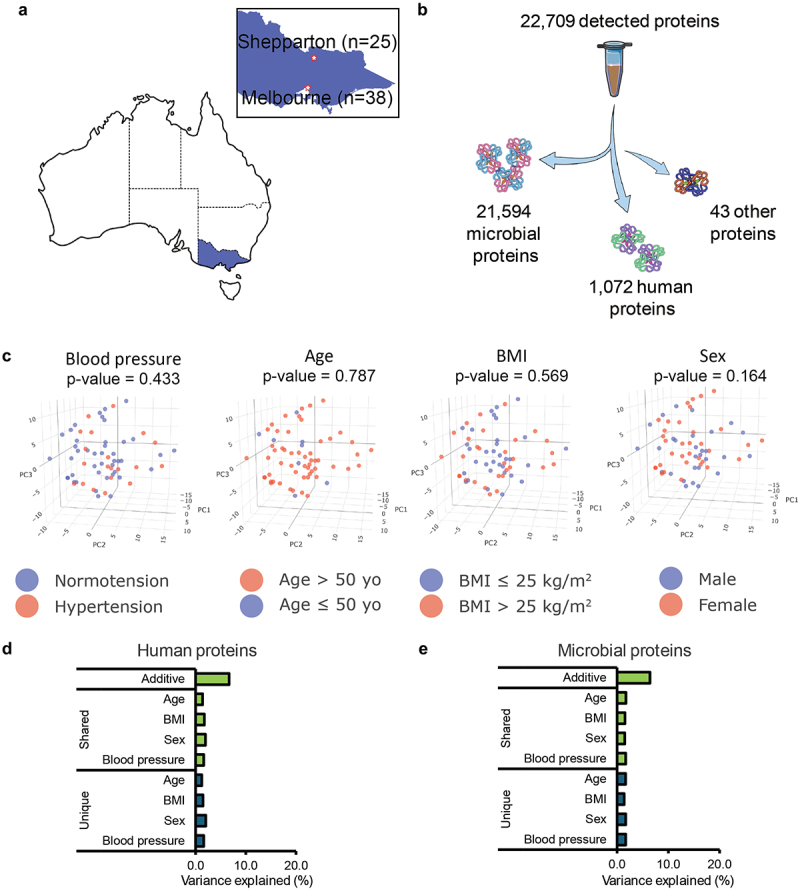
Overview of fecal metaproteome data from the VicGut cohort and the influence of conventional cardiovascular risk factors. (a). Geographic distribution of sample collection sites depicted on a map of Australia and Victoria. Fecal samples were collected from participants at the Baker Heart and Diabetes Institute in metropolitan Melbourne and Shepparton, a regional town in Victoria. (b). Graphical representation illustrating the distribution of detected proteins in the metaproteome dataset and their origins. Among the 22,709 detected proteins 21,594 were of microbial origin, 1,072 were human proteins, and 43 proteins originated from other sources such as food. Some icons are from SciDraw and Bioicons program (CC-BY). (c). PCA plot illustrating the influence of traditional cardiovascular risk factors on gut lumen human protein expression profiles. (d-e). Percentage of variation in d. human and (e). microbial proteins could be explained by four conventional cardiovascular disease risk factors: sex, BMI, age, and blood pressure. The ‘shared model’ represents an additive model incorporating all four risk factors, while the ‘unique model’ indicates separate analyses of individual risk factors. Sample size *n* = 63.

This study complied with the Declaration of Helsinki and was approved by the human research ethics committee of the Alfred Hospital, Melbourne, Australia (approval 415/16 and 477/17). All participants provided informed consent and were recruited between October 2016 and January 2020. The study was registered in the Australian New Zealand Clinical Trials Registry under ACTRN12620000958987. The recruitment and inclusion/exclusion criteria of the healthy cohort (VicGut) and HFpEF patients were described previously.^14,15^ Briefly, for the VicGut, healthy participants were 40–70 years of age, either sex, had body mass index (BMI) 18.5–30 kg/m^2^, were not using BP-lowering medication, and were recruited in two sites across Melbourne and Shepparton (Figure 1a). These participants were healthy, without diabetes or gastrointestinal disorders. The HFpEF patients were diagnosed by a right heart catheterization (RHC), which confirmed a resting pulmonary capillary wedge pressure (PCWP) ≥15 mmHg or exercise PCWP ≥25 mmHg and a left ventricular ejection fraction (LVEF) >50% according to recognized diagnostic criteria for HFpEF. Exclusion criteria included, amongst others, intake of antibiotics or probiotics in the last 6 months.

### Blood pressure measurement and hypertension diagnosis

In the VicGut cohort, office BP was measured using an automatic BP monitor (Omron), and 2–3 measurements were taken.^15^ Twenty-four-hour BP was measured using a calibrated ambulatory ABPM device (AND or SpaceLabs).^15^ Hypertension was diagnosed based on 24-hour systolic BP >130 mmHg and/or diastolic BP >80 mmHg.^16^

### Exercise right heart catheterisation protocol

Exercise RHC was performed using supine cycle ergometry, as described previously.^14^ A 7-F Swan-Ganz catheter was inserted through the brachial or internal jugular vein with the patient under local anesthesia. End-expiratory measurements were taken from the right atrium, right ventricle, pulmonary artery, and pulmonary capillary wedge pressure (PCWP) was measured.

### Food frequency questionnaire

Dietary intake over a period of 12 months was assessed using the Dietary Questionnaire for Epidemiological Studies (DQES) version 3.2, a self-administered and validated food frequency questionnaire (FFQ) developed by the Cancer Council Victoria that reflects the dietary intake of the Australian population.^17^ Dietary intake estimates of 98 nutrients were derived from two Australian databases, AUSNUT 2007^18^ and NUTTAB 2010.^19^ Out of these, the following nutrients were assessed due to their relevance to BP and/or gut microbiome: sodium (mg/day), potassium (mg/day), fiber (g/day), fat (g/day), protein (g/day), fruit (g/day), vegetables (g/day), whole grains (g/day). Dietary fiber intake was classified as adequate or inadequate based on a threshold of 25 g/day, defined according to a meta-analysis on fiber intake and CVD prevalence and mortality.^20^

### Protein extraction from samples and enzymatic digestion

Metaproteomic analyses were performed on the fecal samples of 63 healthy participants and 26 HFpEF patients – these participants still had fecal samples available. Samples were cryo-pulverized and solubilized in 5% sodium dodecyl sulfate (SDS) 10 mm Tris HCL, with heat inactivation at 95°C for 10 minutes followed by centrifugation at 13,000 rcf for 5 minutes. Samples were cleaned by additional three times centrifugation at 13,000 rcf for 5 minutes then processed using the S-trap (Protifi) protocol as per the manufacturer’s instructions.^21^ Briefly, samples were reduced and alkylated using 10 mm TCEP (Thermo, #77720) and 40 mm chloroacetamide (Sigma, C0267-100 G) with incubation at 55°C for 15 minutes. Enzymatic digestion was performed using Trypsin (Promega, V528X) at a 1:50 wt:wt ratio alongside Lys-C at a 1:25 wt:wt ratio (Promega, VA1170) at 37°C for 16 hours. Digestion efficiency was greater than 89% for this analysis.

### TMT labeling and fractionation

Labeling was performed using TMTpro 16plex reagent set (Lot:WD314806, Thermo Scientific) according to the manufacturer’s instructions and utilized a singular reference channel (126) for each fecal sample set utilizing (two per tissue type). Pooled plexes were then fractionated utilizing high-pH RP-HPLC generating 36 fractions that were concatenated into 12 to ameliorate low complexity issues during acquisition. Additionally, a global pooled sample was fractionated into six fractions using the same method, specifically for generating a data-specific database. Each plex has been acquired individually by LC-MS/MS to maximize identifications. For each sample set, labeling efficiency was determined to be greater than 88%, utilizing data-dependent analysis of individually labeled samples.

### Liquid chromatography mass spectrometry protocol

Liquid chromatography-mass spectrometric (LC-MS) analysis was conducted using the Mass Spectrometer and Nano LC system (Dionex Ultimate 3000 RSLCnano). The samples were loaded in an Acclaim PepMap RSLC (75 μm x 50 cm, nanoViper, C18, 2 μm, 100Å; Thermo Scientific) analytical column. The peptides were separated by increasing concentrations of buffer B (80% acetonitrile/0.1% formic acid) and analyzed via 2 kV nano-electrospray ionization with an Orbitrap Eclipse Tribrid mass spectrometer (Thermo Scientific, Bremen, Germany) operated in data-dependent acquisition mode using in-house optimized parameters with 120 minutes of chromatographic separation used for each fraction. Briefly, the acquisition used three FAIMS compensation voltages (−40, −55, −70) operated under standard resolution with an ion transfer tube temperature of 300°C with a carrier gas flow rate of 4.6 L/min. Survey scans were performed at a resolution of 120,000 from 400–1,600 m/z, with a 250% AGC target and ion injection time set to auto. Fragmentation for peptide identification and reporter tag quantification were performed synchronously (10 per duty cycle per compensation voltage) with the fragmentation spectra generated in the ion trap using CID with turbo scan rate; MS3 reporter ion measurements were performed in the orbitrap with a resolution of 50,000. Dynamic exclusion was applied for 60 seconds across all compensation voltages with only one charge state per precursor selected for fragmentation.

### Assembly of a sample-specific database

The sample-specific database was generated using MetaLab software (HGM1.0).^22^ Initially, spectral clustering was applied to the MS/MS spectra from the pooled global fecal samples to reduce dataset redundancy by removing redundant or inferior spectra. This refined list was then searched against a gut microbial gene catalog database, tailored for human microbiomes by MetaLab, to create a candidate protein list for database construction.

### Mass spectrometric data analysis

The raw data files were analyzed using Proteome Discoverer (v2.5.0.400, Thermo Scientific) to obtain protein identifications and their respective reporter ion intensities using in-house standard parameters with sequest. The sample-specific database generated by MetaLab was used for protein identification at a 1% false discovery rate (FDR) alongside a common contaminants database. Reporter ion quantifiers used a unique plus razor with analysis centered on protein groups for shared peptide sequences using all peptides for abundances determination, value output and quantitative values were corrected against stable isotope label impurities according to the manufacturer’s values as per the lot number.

### Metaproteomic downstream analysis

The abundance of proteins was scaled to the sum of the total protein abundance and multiplied by 10^5^ to facilitate subsequent analysis. The abundance of all detected proteins under the corresponding taxonomy was summed and log-transformed before differential analysis to measure biomass of different microbial taxonomies. Only proteins present in > 50% of samples were kept for protein-level analysis. This resulted in 373 gut lumen human proteins and 7,519 high-quality microbial proteins detected in > 50% of samples that were considered as high-quality proteins and were used for the following analyses. Scaled abundance was then log-transformed, and missing values that remained were imputed by the K-nearest neighbor (KNN) method, widely used in current metaproteomic data analysis.^23,24^ K-mean unsupervised clustering was performed using R 4.3 with the default setting.

Limma (v3.56.2)^25^ was used to analyze the protein expression differences or the biomass of microbial taxonomies between different groups. Pathway overrepresentation analysis of human protein was performed using an online tool Enrichr.^26^ The protein-protein interaction network was constructed using the online STRING database.^27^ The co-expression network was constructed using WGCNA (v1.72.5),^28^ and edges with a weight > 0.05 were kept in an outputted network. A motif search on promoter regions was performed using Homer (v4.11).^29^

The random forest (RF) model was trained using the scikit-learn package (v1.1.3) run in a Python 3.10 environment with 100 estimators. Minimum samples per split and leaf were set to 3 to avoid model overfitting.

### UK biobank data processing

The UK Biobank follows the principles of the Declaration of Helsinki and received ethical approval from a human ethics committee (11/NW/0382), with access approved under application number 86,879. The UK Biobank is a comprehensive longitudinal cohort study, comprising over 500,000 participants recruited between 2006 and 2010.^30^ We assessed information from 53,014 participants who had plasma proteomics data available. To filter out low-quality samples and proteins, we excluded proteins detected in fewer than 50% of samples and samples with less than 20% proteins detected, resulting in a final dataset of 52,936 participants and 2,920 proteins. Missing values in the proteomics data were imputed using the KNN method, consistent with our approach for metaproteomic datasets.

We included 34,311 participants who were not taking BP-affecting medications in further analyses. Major adverse cardiovascular events (MACE) were defined following a method outlined by Zheng & Tavares et al. (2024).^31^ Briefly, MACE included hospital admissions and death registers (ICD code I20.0, I21.*,I24.8, I24.9, I50.*, I63.*, I64.*) as well as surgical procedures (K40.*, K41.*, K42.*, K43.*, K44.*, K45.*, K46.*,K49.*, K50.*,K75.*) associated with acute coronary syndrome (ACS), ischemic stroke, or heart failure (HF). The circulating protein scores were calculated using the following method. First, we extracted circulating proteins also detected in the metaproteomic datasets. Proteins with zero importance in the human protein-based random forest model were excluded. The metaproteomic circulating protein scores were subsequently calculated as a weighted sum based on the random forest model’s importance. Specifically, the score was computed as ∑ (random forest importance × upregulated protein level) − ∑ (random forest importance × downregulated protein level). High scores indicate a protein expression profile similar to the low-risk group we identified.

### Statistical analysis

Statistical tests and data visualization were performed using R 4.3. Normally distributed data was analyzed using a two-tailed Welch two-sample t-test. Non-normally distributed data was analyzed using a two-tailed Wilcoxon-Mann-Whitney test or permutation test for a small sample size (*n* < 30). Binomial data, such as sex and living area among patients, were analyzed using the chi-square (Χ^2^) test. In contrast, other binomial data were analyzed using Fisher’s exact test due to the presence of low frequencies. Time-to-event analyses were conducted using a Cox proportional hazards model with the survival (version 3.6.4),^32^ survminer (version 0.4.9), and adjustedCurves (version 0.11.2)^33^ packages in R. Permutational analysis of variance (PERMANOVA) test based on Euclidean distance was conducted using the R package vegan (https://CRAN.R-project.org/package=vegan) to assess the equivalence of centroids among different groups. For high-dimensional data analysis, p-values were adjusted using the Benjamini – Hochberg FDR method with a cutoff of q < 0.05. Significance was determined as P or q < 0.05.

## Results

### Traditional cardiovascular risk factors poorly capture expression profiles of gut lumen human protein and microbial proteins

Healthy participants were recruited from Melbourne and Shepparton in Victoria, Australia – their baseline characteristics are detailed in Table 1 and Figure 1a. Fecal samples from these participants were analyzed using mass spectrometry to determine the fecal metaproteome. A total of 22,709 proteins were identified in the metaproteome data from these participants (Figure 1b). Among these 21,594 were classified as microbial proteins originating from seven bacterial phyla (Figure 1b and S1A), with the majority originating from the Bacillota and Bacteroidota phyla, and a smaller proportion from Campylobacterota, Pseudomonadota, Thermodesulfobacteriota, and Verrucomicrobiota. This taxonomic distribution aligns with the well-defined structure of the human gut microbiota.^38^ Additionally, 1,072 were identified as human proteins, representing gut lumen human proteins, and 43 proteins originated from other eukaryotic organisms, potentially linked to food intake (Figure 1b).

**Table 1.** Characteristics of participants included in the healthy cohort and low- and high-risk groups identified.

Variable	Healthy participants (*n* = 63)	Cluster 1 (Low-risk) (*n* = 26)		Cluster 2 (High-risk) (*n* = 37)		P-value
Age	60^34,35^	60^34,36^		60^35,37^		0.695
Female	54.0% (34)	65.4% (17)		40.6% (17)		0.205
Regional patients	39.7% (25)	53.8% (14)		29.7% (11)		0.096
BMI (kg/cm^2^)	25.036 ± 2.871	24.801 ± 2.655		25.202 ± 3.038		0.581
WHR	0.866 ± 0.088	0.856 ± 0.078		0.874 ± 0.094		0.419
Daytime SBP (mmHg)	128.992 ± 14.974	126.789 ± 15.769		130.539 ± 14.405		0.34
Daytime PP (mmHg)	49.926 ± 8.795	48.710 ± 9.584		50.781 ± 8.223		0.3754
Nighttime SBP (mmHg)	113.680 ± 16.523	108.597 ± 14.637		117.252 ± 17.018		0.035*
Nighttime PP (mmHg)	46.473 ± 8.978	43.393 ± 8.870		48.637 ± 8.516		0.023*
Overall HR (mmHg)	73.680 ± 12.066	77.673 ± 11.334		70.873 ± 11.913		0.025*
Fiber intake (g/day)	23.856 ± 8.796	24.915 ± 8.992		23.092 ± 8.699		0.428
Sodium (g/day)	1.978[1.643,2.539]	2.164[1.811,2.766]		1.950[1.53,2.24]		0.046*
Potassium (g/day)	3.622[2.967,4.348]	3.987[3.318,4.507]		3.408[2.760,3.956]		0.061
Sodium/Potassium	0.582 ± 0.171	0.596 ± 0.204		0.571 ± 0.143		0.605
CVD risk-associated dietary factor analysis by sex						
	Male			Female		
	Cluster 1 (Low-risk)	Cluster 2 (High-risk)	P-value	Cluster 1 (Low-risk)	Cluster 2 (High-risk)	P-value
Fiber intake (g/day)	30.581 ± 8.254	23.242 ± 6.943	0.022*	21.915 ± 8.040	22.925 ± 10.548	0.756
Sodium (g/day)	2.080[1.811,2.766]	1.924[1.573,2.324]	0.143	2.301[1.867,2.786]	1.919[1.486,2.242]	0.121
Potassium (g/day)	3.987[3.508,4.507]	3.128[2.805,3.700]	0.021*	3.896[3.124,4.527]	3.642[2.744,4.221]	0.769
Sodium/Potassium	0.565 ± 0.206	0.584 ± 0.124	0.772	0.614 ± 0.207	0.558 ± 0.164	0.4

Binomial data are presented as percentage (number of individual). Normally distributed data are presented as mean ± standard deviation. Non-normally distributed data are presented as median [Q25, Q75]. BMI: Body mass index; HR: Heart rate; SBP: Systolic blood pressure; PP: Pulse pressure; WHR: Waist-to-hip ratio. *p < 0.05.

Based on previous findings linking gut microbiota to cardiovascular health,^39,40^ we initially investigated whether the expression of gut lumen human proteins or microbial proteins differed between individual traditional risk factors for CVD, including hypertension, age (>50 years versus ≤50 years), BMI (>25 kg/m^2^ or ≤25 kg/m^2^), and sex (Figure 1c and S1B). None of these were significantly different. The variation in expression levels of both human and microbial proteins within the gut lumen could only be minimally explained by these traditional factors (less than 10%) (Figures 1d-e and S1B). BP, for example, explained only 1.6% of the variance for gut lumen human proteins (*p* = 0.433) and 1.7% for microbial proteins (*p* = 0.397) (Figure 1d). Even an additive model incorporating all these factors could explain only 6.7% of the variance in gut lumen human proteins and 6.4% of the variance in microbial proteins (Figure 1e). Although dietary fiber intake has been previously associated with reduced CVD risk by promoting the production of short-chain fatty acids (SCFAs) by the gut microbiome,^11,41^ we found a significant difference only in the human proteins (*p* = 0.027), with seven muscle-related proteins underrepresented in participants with inadequate dietary fiber intake (Figure S2C-E). These could be potentially related to gut dysmotility observed in constipation, an emerging risk factor for CVD.^31,42,43^

### Unsupervised clustering identified distinct cardiovascular risk groups through metaproteome expression

**Figure 2.**
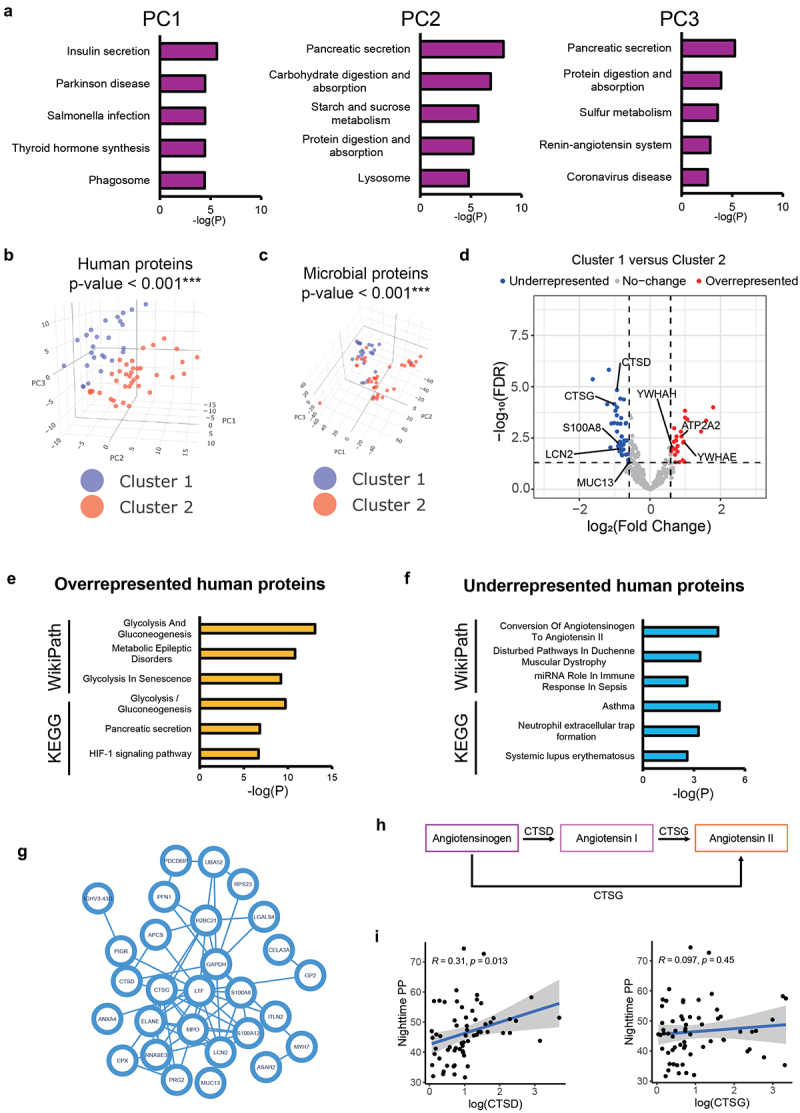
Differential expression of gut lumen human protein. A. Overrepresentation analysis of the top 50 proteins contributing to PC1-PC3 of human protein PCA using the KEGG database. The diverse functions observed in the top contributor proteins of PC1 suggest their association with individual differences, and thus its capacity to define groups with distinct cardiovascular disease risk is limited. In contrast, the top contributor proteins of PC2 and PC3 are primarily related to gut immune function and absorption, both of which are closely linked to human cardiovascular health. B. Principal component analysis (PCA) plot illustrating the outcome of unsupervised clustering. Two distinct clusters, cluster 1 and cluster 2, are clearly outlined by this supervised K-mean model (PERMANOVA p-value <0.001, based on Euclidean distances of all gut lumen human proteins). C. Two clusters based on gut lumen human protein expression profiles also effectively distinguishes microbial protein expression profiles (PERMANOVA p-value <0.001, based on Euclidean distances of all microbial proteins). D. Volcano plot showing differentially expressed gut lumen human proteins between the low-risk and high-risk groups. Fold changes were calculated as low-risk group versus high-risk group. E-F. Overrepresentation analysis using the KEGG and Wikipathway databases for E. overrepresented and F. underrepresented proteins. G. A PPI network of underrepresented gut lumen human proteins in low-risk group. H. Schematics depicting the angiotensin converting by CTSD and CTSG protein. I. Correlation analysis between gut lumen CTSD and CTSG protein levels and nighttime PP. Sample size *n* = 63.

We then used K-means, an unsupervised machine-learning algorithm renowned for its robustness, to cluster samples based on human and microbial protein expression. To select proper input PCs for cluster, we examined the top 50 human proteins contributing to different principal components. PC1 has a diverse function and may influence the individual differences. This included pathways related to insulin secretion and pathogenic bacterial infection (Figures 2A and S3A). Conversely, proteins contributing to PC2 included those involved in the activation of the immune system such as lipocalin-2 (LCN2) and several immunoglobulins (Figures 2a and S3B, Table S1). Proteins contributing to PC3 included angiotensin converting enzyme (ACE, key for BP regulation) and mucins involved in the gut epithelial barrier (MUC12, MUC13), and predominantly played a role in pathways related to pancreatic secretion, protein digestion and absorption, and the renin-angiotensin system (RAS)^44^ (Figures 2a and S3C, Table S1). Therefore, we did K-means analysis based on PC2 and PC3 for their relevance to CVD risk. This clustering method distinctly categorized samples into two clusters (Figure 2b-c), with a p-value <0.001 (under the detectable lower limit of PERMANOVA), indicating significant differences in human (Figure 2b) and microbial protein expression (Figure 2c) between the clusters.

A comparison of gut lumen human protein expression profiles between the two clusters revealed 25 overrepresented proteins in Cluster 1 (Figure 2d, Table S2). These proteins formed a protein-protein network (Figure S4A). Key overrepresented pathways included pancreatic secretion, protein digestion and absorption, glycolysis/gluconeogenesis, and proteins involved in insulin signaling-mediated glucose transport proteins including YWHAE and YWHAH (Figure 2e, Table S4). Among these, we identified ATP2A2, a protein associated with systolic BP in a genome-wide association study,^45^ which dysfunction leads to increased BP via oxidative stress;^46^ CELA3B, an elastase that may be involved in cholesterol metabolism and transport in the intestine;^47^ and MYO1D, a myosin that maintains gut epithelial integrity.^48^

Forty proteins were underrepresented in Cluster 1 (Figures 2d, Table S3). Relevant underrepresented pathways included neutrophil extracellular trap formation (a bacterial defense mechanism) and conversion of angiotensinogen to angiotensin II, and those associated with auto-immune regulation (including miRNA role in immune response in sepsis, asthma, systemic lupus erythematosus) (Figure 2f, Table S4). These proteins formed a tight protein-protein interaction network (Figure 2G). Central to this network were the intestinal inflammation marker calprotectin (S100A8)^49^ and an associated protein, S100A12.^50^ LCN2 could interact with another MUC13, a negative regulator of tight junction.^51^ Besides, another underrepresented protein, proteoglycan 2 (PRG2), also called eosinophil major basic protein (MBP), could suppress occludin expression in the gut epithelium and destabilize the gut epithelial barrier.^52^ These findings indicate that gut epithelium barrier integrity may be compromised in Cluster 2. We also found two angiotensin-converting enzymes, cathepsin D (CTSD)^53^ and cathepsin G (CTSG)^54^ (Figure 2h), which may drive an exaggerated RAS observed in hypertension. CTSD is associated with cardiovascular risk,^55^ worse CVD outcomes,^56^ and pro-inflammatory factors.^34,37^ Indeed, CTSD, but not CTSG, was significantly correlated to night pulse pressure in our dataset (Figure 2i). The small intestine and colon are among the tissues with the highest CTSD and CTSG expression levels (Figure S4B-C), suggesting a role of gut CTSD in BP regulation. LCN2 also serves as a neutrophil gelatinase-associated lipocalin previously shown to lead to cardiac hypertrophy and failure,^57^ which also acts as part of the innate immune system, limiting bacterial growth^58^ and preventing intestinal inflammation.^59^ Together with LCN2, other key neutrophil-relevant proteins, including ELANE and MPO, were also underrepresented.

### Fecal metaproteomics clusters are associated with cardiovascular risk factors

The combination of proteins and pathways related to CVD and inflammation prompted the hypothesis that these clusters may represent distinct CVD risk groups. To validate this hypothesis, we analyzed medical and demographic data of participants corresponding to the clusters identified in the metaproteome data (Table 1). Indeed, we found that participants in Cluster 1 had ~9 mmHg significantly lower night systolic BP and ~5 mmHg pulse pressure relative to Cluster 2, indicative of lower CVD risk.^60^ These were independent of sex, BMI, and age. Accordingly, we classified Cluster 1 as a low cardiovascular risk group (referred below as ‘low-risk group’) and Cluster 2 as a high cardiovascular risk group (‘high-risk group’). Notably, the dietary patterns of the two clusters differed by sex. Low-risk males had a significantly higher dietary fiber and potassium intake. The low-risk group had a higher overall sodium intake (Table 1). However, after adjusting for sex, there was no significant difference in sodium intake between the high-risk and low-risk groups (Table 1).

Due to the contribution of diet to both the gut microbiome and CVD, we next assessed the variance explained by common dietary factors considered CVD risk factors across the two clusters. Collectively, dietary factors explained ~ 20% of the total variance in differentially expressed human proteins between the two groups (Figure S5A), underscoring the importance of dietary factors in mitigating CVD risk, albeit not the sole determinant. Notably, high potassium intake was observed in the low-risk group and could significantly explain ~ 5% variance in an additive model (Figure S5A).

### Regulatory factors’ contribution to cardiovascular risk groups

To identify potential key regulators of differentially expressed gut lumen human proteins between the two groups, we extracted the promoter sequences, defined as 1 kilobase upstream to 0.1 kilobase downstream of the transcription start site (−1kb to + 0.1kb of TSS), of coding genes associated with the differentially expressed proteins (Figures S5B-C). Subsequently, we conducted a motif search on the extracted promoter regions. Notably, the sequence motif of hypoxia-inducible factor (HIF) was significantly enriched in the promoters of genes encoding overrepresented proteins (Figures S5B-C). A moderate level of HIF1α expression is essential for the normal function of insulin-producing β cells,^61,62^ which aligns with the observed association of upregulated proteins of HIF1 signaling, glycolysis/gluconeogenesis, and pancreatic secretion in overrepresented proteins. Conversely, the sequence motif of myocyte enhancer factor-2 (MEF2) plays a role in the pathology of hypertension-induced cardiac hypertrophy,^63^ was enriched in promoters of genes encoding underrepresented proteins.

### Differential gut bacteria proteome and cardiovascular risk factors

**Figure 3.**
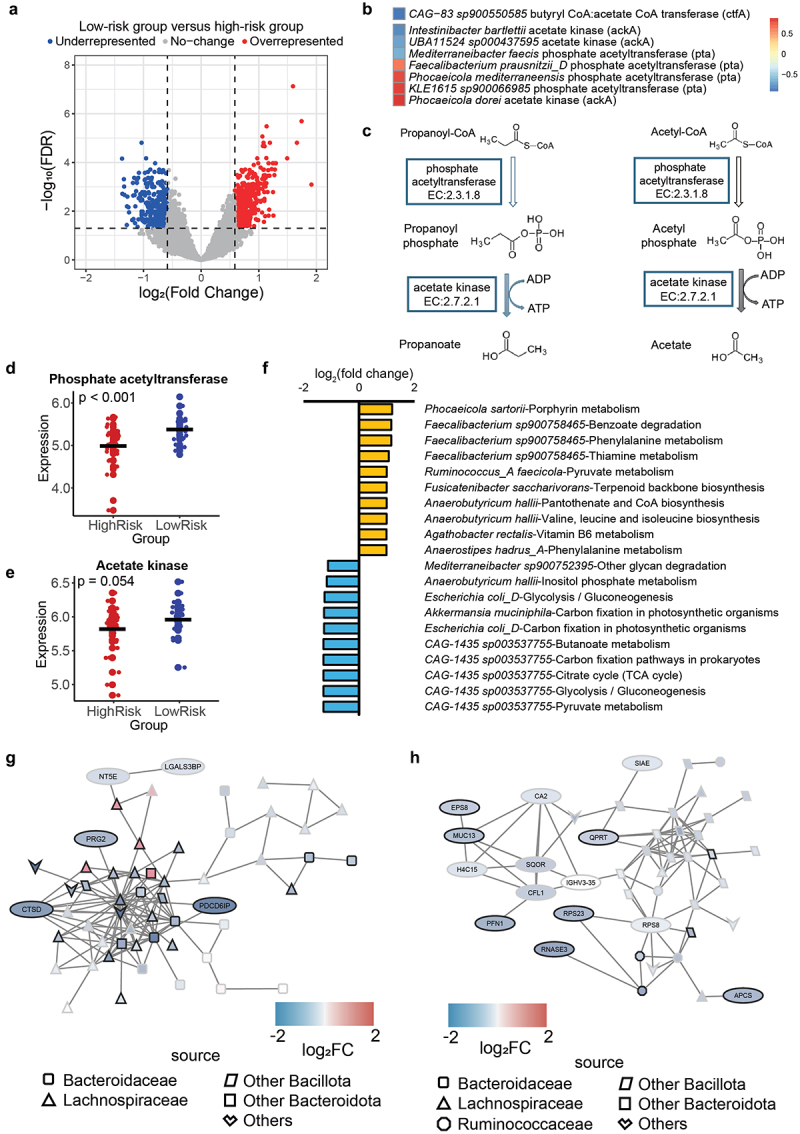
Differential expression of microbial proteins and pathways between two groups and crosstalk between gut lumen human proteins and microbial proteins. A. Volcano plot displaying differentially expressed gut microbial proteins between the low-risk and high-risk groups. Fold changes were calculated as the low-risk group versus the high-risk group. B. Identification of significantly differentially expressed microbial enzymes involved in directly producing short-chain fatty acids (SCFAs). C. Schematics depicting the catalytic process by which acetate kinase and phosphate acetyltransferase facilitates the chemical reaction leading to the production of acetate and propanoate. D-E. Comparison of the overall expression level of D. phosphate acetyltransferase and E. acetate kinase in gut microbiome between low-risk group and high-risk group. F. Bar plot showing the top ten significantly overrepresented and underrepresented KEGG pathways by species in the low-risk group. Fold changes were calculated as the low-risk group versus the high-risk group. G-H. Presentation of two co-expression networks of gut lumen human proteins and microbial proteins, both found to be significantly downregulated in the low-risk group. Sample size *n* = 63.

Within the microbial proteins, we identified 430 significantly higher and 276 lower expressed proteins in the low-risk group (Figure 3a, Tables S5 and S6). Analysis of the source of these differentially expressed gut microbial proteins revealed that most underrepresented proteins in the low-risk group originated from the Bacillota (Firmicute) phylum. Conversely, underrepresented proteins in the high-risk group exhibited a significantly higher proportion of Bacteroidota-sourced proteins (Figure S6A). Classification based on Clusters of Orthologous Groups (COG) categories demonstrated that the most enriched functions of differentially expressed gut microbial proteins were associated with translation and metabolism (COG categories J, G, and C), indicating alterations in the bioactivity of the source gut microbiome. Notably, proteins related to translation (COG category J) were significantly overrepresented among overrepresented gut microbial proteins in the low-risk group (Figure S6B).

Considering that increased microbial SCFA production and/or sensing has been linked to reduced BP and CVD risk,^11,15,57,64^ we hypothesized that SCFA production is elevated in the low-risk group. To test this hypothesis, we selected enzymes involved in the final production steps of SCFAs and examined their expression between the two groups. We observed a significant increase in the expression of acetate kinases (ackA) and phosphate acetyltransferase (pta) in several SCFA-producing bacteria, such as *Phocaeicola dorei* (*Bacteroides dorei*) and *Faecalibacterium prausnitzii* (Figure 3b-c), suggesting elevated levels of both acetate and propanoate production. Moreover, among four detected enzymes involved in the final production steps of SCFAs (Figure 3d-e; Figure S6C-D), the overall expression of pta in the gut microbiome population was significantly higher in the low-risk group (Figure 3d), while the overall expression of ackA was increased but did not reach statistical significance (*p* = 0.054, Figure 3e). There was no change in the levels of the other two enzymes, butyrate kinase and butyryl-CoA:acetate-CoA transferase (Figure S6C-D). Furthermore, analysis of metabolism-related KEGG pathways in each microbiota species revealed increased activity in SCFA-production pathways, such as pyruvate metabolism in *Ruminococcus_A faecicola*, and amino acid metabolism pathways contributing to SCFA production of species in *Faecalibacterium* and *Anaerobutyricum*, which are well-described SCFA-producing genera (Figure 3f).^36^ Thus, the evidence above supports that the low-risk group has a gut microbiome with enhanced SCFA production capacity, which may contribute to the lower BP observed in these participants.

A previous randomized clinical trial found vitamin B1 supplementary could significantly lower BP.^65^ KEGG pathways related to thiamine (vitamin B1) and pyridoxine (vitamin B6) metabolism are upregulated in the gut microbiome of the low-risk group. Vitamin B1,^35^ B2,^66^ B3^67^ and B6^68^ intake are all associated with lower BP. Moreover, vitamins B1, B2 and B3 were all significantly higher in the low-risk group, but not vitamin B6 (Table S7).

### Crosstalk between the gut microbiome and human proteins

We hypothesized that there were also crosstalks between differentially presented microbiome proteins and gut lumen human proteins that may explain some CVD risk. To investigate this hypothesis, we explored potential co-expression networks between human proteins and gut microbiome proteins, which are strong indicators of crosstalk between microbiome-to-host communication. Indeed, we identified two co-expression networks significantly underrepresented in the low-risk group, likely associated with CVD risk. In the first co-expression networks, we found one downregulated network in the low-risk group that included CTSD (involved in the RAS) and PRG2, which is a proinflammatory factor destabilizing the intestinal barrier^52^ (Figure 3g). In the second network (Figure 3h), several proteins known to play a relevant role in CVD development,^69^ including S100A8, MUC13 and profilin 1 (PFN1), were identified.

### Fecal metaproteome signature in heart failure

**Figure 4.**
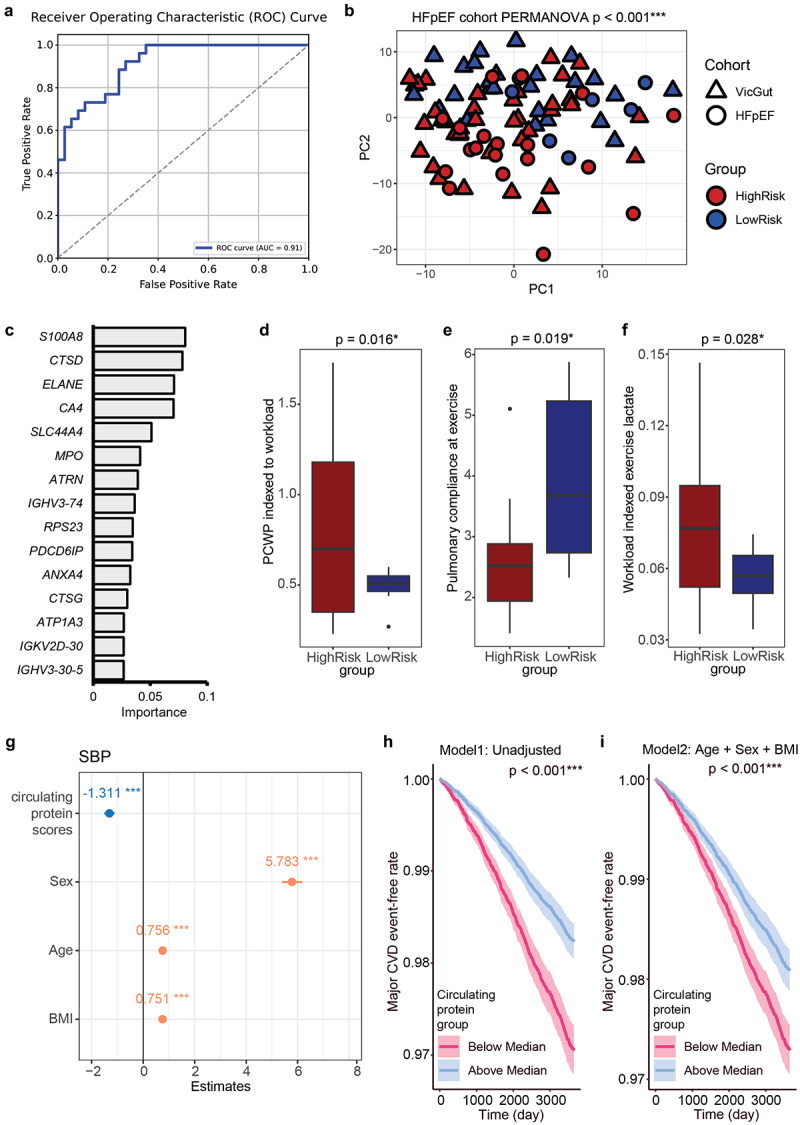
Machine learning model showing the relationship between human proteins and cardiovascular risk. A. Receiver operating characteristic (ROC) plot illustrating the results of 5-fold cross-validation of the constructed random forest model based on differentially expressed human proteins. B. PCA dimensionality reduction visualization of prediction results for heart failure with preserved ejection fraction (HFpEF) patients using the trained human protein-based random forest model. C. Presentation of the top 15 signature human proteins with the highest feature importance in the random forest model. D-F. Comparison of D. PCWP indexed to workload and E. Pulmonary compliance at exercise F. Workload indexed exercise lactate in HFpEF patients predicted as high-risk and low-risk. Sample size *n* = 26.G. Age, BMI, sex adjusted regression model between SBP and standardized circulating protein scores derived from differentially expressed gut lumen human protein between high-risk and low-risk group. H.-I. Two 10-year follow-up time-to-event analysis H. unadjusted (*p* < 0.001***) and I. age, sex BMI adjusted (*p* < 0.001***) showing the incident rate of major CVD events in two groups based on circulating protein scores calculated. Sample size *n* = 26 for the HFpEF cohort and *n* = 34,311 for the UK biobank dataset.

Because distinct expression patterns of gut lumen proteins were associated with clusters with varying risk for CVD (Figure 2c), we further investigated the differentially expressed microbial and human proteins in 26 patients diagnosed with HFpEF, an increasingly common form of heart failure in which high BP is a key risk factor. We hypothesized that the signature of protein expression in the gut lumen could serve not only as a predictor of CVD risk but also as an indicator of clinical phenotypes. HFpEF patients were, on average, 68 ± 7.5 years old, had 32.8 ± 5.8 kg/m^2^ BMI, 113.2 ± 96 ng/L brain natriuretic peptide (BNP), 143.7 ± 19.1 mmHg office systolic BP, and 77% were female, as described previously.^14^ These patients had a microbiome composition similar to healthy participants, with a higher abundance of the Bacillota and Bacteroideota phyla (Figure S7A). We trained two random forest models based on human proteins and microbial proteins, separately, to predict low- and high-risk individuals based on the healthy cohort data and compared their performance (Figures 4a-c, Figures S7B-D). Testing via 5-fold cross-validation yielded a high area under the curve (AUC) of 0.80 based on the microbial protein-based model (Figure S7C) and 0.91 for the human protein-based model (Figure 4a) on the Receiver Operating Characteristic plot, indicating both models were well-trained. We employed these two random forest models to classify HFpEF patients into low- and high-risk groups (Figure 4b, Figure S7D). In both models, the high-risk and low-risk groups within the HFpEf cohort did not show significant differences in age, BMI and sex (Table S8). In the human protein-based model, intestinal inflammation marker S100A8^50^ and angiotensin-converting enzymes CTSD and CTSG show high evidence (Figure 4c), suggesting their pivotal associations with cardiovascular risks. In the microbial protein-based model, proteins from Bacillota species emerged as pivotal in distinguishing between the low- and high-risk groups, as evidenced by their high feature importance (Figure S7B).

Subsequently, we compared PCWP indexed to workload,^70^ pulmonary compliance at exercise,^71^ and workload-indexed exercise lactate^72^ between high-risk and low-risk HFpEF patients identified by the two prediction models. These three indicators are well-established gold standards for evaluating clinical outcomes in HFpEF patients, associated with worse clinical outcomes.^70–72^ In the human protein-based model, the high-risk group showed significantly higher PCWP (Figure 4d) and lactate (Figure 4f) after exercise indexed to workload and lower pulmonary compliance at exercise (Figures 4e). Conversely, the microbial protein-based model identified a high-risk group with significantly lower pulmonary compliance during exercise (Figure S7F), but no significant differences for the other two indicators between the high- and low-risk groups. These findings demonstrate that both the human and microbial protein-based models could effectively predict clinical outcomes in HFpEF patients, with the human protein-based model showing higher performance.

### Population-level impact of differentially expressed human proteins on cardiovascular health detected in plasma

Given the higher performance of the human protein-based model, to further validate the impact of differentially expressed human proteins between the high-risk and low-risk groups on cardiovascular health at the population level, we extracted human proteins that were differentially expressed between the high-risk and low-risk groups and overlapped with circulating proteins detected in the UK Biobank plasma proteomics dataset (Figure S8A). We calculated random forest importance-weighted circulating protein scores (see methods and Figure S8B). Higher circulating protein scores indicate a greater similarity to the protein signature identified in the low-risk group. We found that one standard deviation (SD) increase in this circulating protein score was associated with a reduction of 2.9 mmHg in systolic BP in an unadjusted model (*p* < 0.001). After adjusting for age, BMI, and sex, a one SD increase in the circulating protein score was associated with a 1.3 mmHg reduction in SBP (*p* < 0.001, Figure 4G). We then categorized the 34,311 UK Biobank participants based on whether their scores were above or below the population median. Two time-to-event models were developed to evaluate the occurrence of major CVD events within 10 years after the assessment: Model 1 was unadjusted, while Model 2 was adjusted for age, BMI, and sex (Figures 4H-I, Table S9). The results showed that participants with circulating protein scores above the median had a significantly lower incidence of CVD events within the next 10 years.

## Discussion

Here, we report on fecal metaproteomics analysis in healthy and heart failure cohorts showing that human and gut microbial proteins can identify individuals at lower and higher risk of CVD. The low-risk group presented with lower night systolic BP (by 9 mmHg), angiotensin-converting enzymes and pro-inflammatory intestinal responses, and higher SCFA production capacity by the gut microbiome and sex-specific dietary potassium and fiber. We then used machine-learning analysis based on differential microbial and human protein signatures observed in the healthy low- and high-risk groups that identified HFpEF patients with worse clinical phenotypes (i.e., increased PCWP and lactate after exercise indexed to workload and reduced pulmonary compliance). Moreover, when evaluated in plasma samples, the low-risk protein signature was associated with lower systolic BP and long-term cardiovascular risk at the population level. This suggests fecal metaproteomics data may be able to predict long-term cardiovascular outcomes and that several mechanisms that lead to these outcomes may develop in the gut. These could include, for example, a decrease in SCFA production leading to the breakdown of the gut epithelial barrier, passage of microbial metabolites and/or components to the intestinal tissue and activation of inflammatory pathways that contribute to the development of CVD.^15,73^

Unsupervised learning based on gut lumen human protein expression identified two distinct groups of individuals. Proteins highly expressed in the low-risk group primarily relate to pancreatic secretion and glycolysis/gluconeogenesis, which may reflect better glucose control. While all participants from the VicGut were healthy and a previous diagnosis of type 1 or type 2 diabetes was an exclusion criterion, we did not have fasting glucose information available to add to our model. Moreover, the low-risk group had an underrepresentation of proteins associated with inflammation (e.g., calprotectin, LCN2 and other neutrophil extracellular trap formation proteins) and gut epithelial barrier degradation (e.g., MUC13). These differences in protein expression profiles could be influenced by dietary patterns such as fiber or potassium intake, suggesting a link between diet-related metabolic alterations and subsequent effects on the gut microenvironment and cardiovascular health. This may provide novel insights into the contribution of diet via the gut microbiome to the development of CVD.

Consistently with their lower night systolic BP, which is a risk factor for cardiovascular mortality,^74^ the low-risk group had an underrepresentation of proteins associated with the conversion of angiotensinogen to angiotensin I (CTSD, CTSG) and angiotensin I to angiotensin II (CTSG),^75^ as part of the traditional arm of the RAS. The significant positive correlation between CTSD and nighttime pulse pressure suggests intestinal CTSD could play a role in systemic BP regulation. It remains unclear, however, whether these proteins are involved in the systemic or only intestinal RAS.

Studies in recent years showed that the host’s dietary intake significantly influences the composition of the gut microbiome and their production of secondary metabolites.^10^ Indeed, we observed that the gut microbiome in the low-risk group exhibited a significantly higher capacity for producing SCFAs, which reduces BP and associated end-organ damage in animal models and untreated hypertensive patients by driving a gut-cardiorenal axis.^11,64^ This involved, in particular, higher levels of pta, a critical enzyme that converts the precursors of acetate and propionate into their final forms.

The metaproteomic data allowed us to predict the severity of phenotypes of patients with HFpEF. For instance, our prediction model based on both human and microbial proteins successfully categorized HFpEF patients into high- and low-risk groups, with significant differences in PCWP, lactate concentration and pulmonary compliance during exercise, which has implications for clinical outcomes associated with aerobic capacity and survival.^76,77^ The same proteins, when detected in the systemic circulation, were associated with significantly higher BP and long-term risk of MACE in a population-based cohort from European ancestry. Collectively, this suggests that the metaproteomic changes observed in healthy individuals may predict long-term cardiovascular outcomes. Some of the mechanisms we identified, such as SCFA production, angiotensin production and inflammation, may be involved.

We acknowledge this study had limitations. Firstly, we did not establish causality in this study. However, there are no *in vitro* or *in vivo* models where we could emulate the combination of human and microbial proteins and their systemic effect on the host, as observed here. Secondly, the mapping of microbial proteins identified remains poorly understood, highlighting the need for further investigations and potential reanalysis of this dataset once better tools are available. This challenge partly arises because many gut microbial species have only been characterized recently by non-culture-dependent methods such as metagenomics. Thirdly, the sample size of our metaproteomics dataset, while unique in a CVD setting and larger than previous studies using metaproteome techniques to study human gut environments, was still relatively small. While we validated the findings using plasma proteomics, larger metaproteomics cohorts will be needed to validate the current findings, and other CVD risk factors, such as fasting glucose and cholesterol, could be included.

In conclusion, traditional cardiovascular risk factors could not clearly distinguish human gut lumen proteins nor gut microbial protein expression profiles in our dataset. By introducing machine learning models, we successfully identified two distinct groups with significantly different cardiovascular risk factors based on their unique protein expression profiles. Besides uncovering molecular mechanisms associated with these low- and high-risk groups, our findings unveil some of the intricate interplay between diet, gut microenvironment, gut microbiome, and the cardiovascular system that result in differential CVD risk, including long-term risk. Our results underscore the complexity of these interactions, as metaproteomic changes could not be solely attributed to a single factor. This highlights the necessity for applying machine learning models capable of capturing the complicated relationships between risk factors and phenotypes compared to conventional statistical methods.

## Highlights

- Fecal metaproteomic analyses identified human intestinal and microbial proteins associated with low- and high-risk cardiovascular phenotypes in otherwise healthy individuals.
- Low-risk individuals had 9 mmHg lower night blood pressure and expression of angiotensin-converting enzymes (i.e. CTSD, CTSG) and inflammatory proteins (e.g. LCN2, S100A8/calprotectin), and higher intake of dietary potassium and fiber in males, expression of proteins associated with pancreatic secretion and glycolysis/gluconeogenesis, and a gut microbiome with a higher capacity for short-chain fatty acid production relative to high-risk individuals.
- Both gut microbial and human proteins within the high-risk healthy individuals could predict the severity of phenotypes in patients with heart failure with preserved ejection fraction (HFpEF) and long-term risk of a major cardiovascular event in a large population-based cohort.

## Supplementary Material

Online_Supplemental_metaproteomics_clean.docx

## Figures and Tables

**Table:** 

Body mass index	BMI
Blood pressure	BP
False discovery rate	FDR
Heart failure with preserved ejection fraction	HFpEF
Kilobase	KB
Principal component	PC
Principal component analysis	PCA
Receiver Operating Characteristic	ROC
Short-chain fatty acids	SCFAs
Sodium dodecyl sulphate	SDS
Transcription start site	TSS

## Data Availability

This research has been conducted using the UK Biobank Resource under Application Number 86,879. This work uses data provided by patients and collected by the NHS as part of their care and support. All original data used in this article is available upon request.
