# Supplementary material for: Faecal metaproteomics analysis reveals a high cardiovascular risk profile across healthy individuals and heart failure patients
Source: Gut Microbes. 2024 Dec 22;17(1):2441356. doi: 10.1080/19490976.2024.2441356 (PMC12931699; doi:10.1080/19490976.2024.2441356)
Supplement: Online_Supplemental_metaproteomics_clean.docx [file KGMI_A_2441356_SM9693.docx]

**Online Supplementary file to**

**Faecal metaproteomics analysis reveals a high cardiovascular risk profile across healthy individuals and heart failure patients**

**Running title:** Metaproteomics and cardiovascular risk

Chaoran Yang^1^, Leticia Camargo Tavares^1^, Han-Chung Lee^2^, Joel R. Steele^2^, Rosilene V. Ribeiro^3^, Anna L. Beale^4,6^, Stephanie Yiallourou^5^, Melinda J. Carrington^5^, David M. Kaye^4,6,7^, Geoffrey A. Head^8,9^, Ralf B. Schittenhelm^2^, Francine Z. Marques^1,4,10^*

1 Hypertension Research Laboratory, School of Biological Sciences, Faculty of Science, Monash, Clayton, Australia; 2 Monash Proteomics & Metabolomics Platform, Monash Biomedicine Discovery Institute & Department of Biochemistry and Molecular Biology, Monash University, Melbourne, Australia; 3 Charles Perkins Centre, University of Sydney, Sydney, Australia; 4 Heart Failure Research Laboratory, Baker Heart and Diabetes Institute, Melbourne, Australia; 5 Preclinical Disease and Prevention Unit, Baker Heart and Diabetes Institute, Melbourne, Australia; 6 Department of Cardiology, Alfred Hospital, Melbourne, Australia; 7 School of Translational Medicine, Faculty of Medicine Nursing and Health Sciences, Monash University, Melbourne, Australia; 8 Neuropharmacology Laboratory, Baker Heart and Diabetes Institute, Melbourne, Australia; 9 Department of Pharmacology, Faculty of Medicine Nursing and Health Sciences, Monash University, Melbourne, Australia; 10 Victorian Heart Institute, Monash University, Clayton, Australia.

***Corresponding author**: Prof Francine Marques, Hypertension Research Laboratory, School of Biological Sciences, Faculty of Science, Monash University, Melbourne, Australia, Phone: +61-03-9905 6958. E-mail: [francine.marques@monash.edu](mailto:francine.marques@monash.edu)

**Supplementary Figures**

**
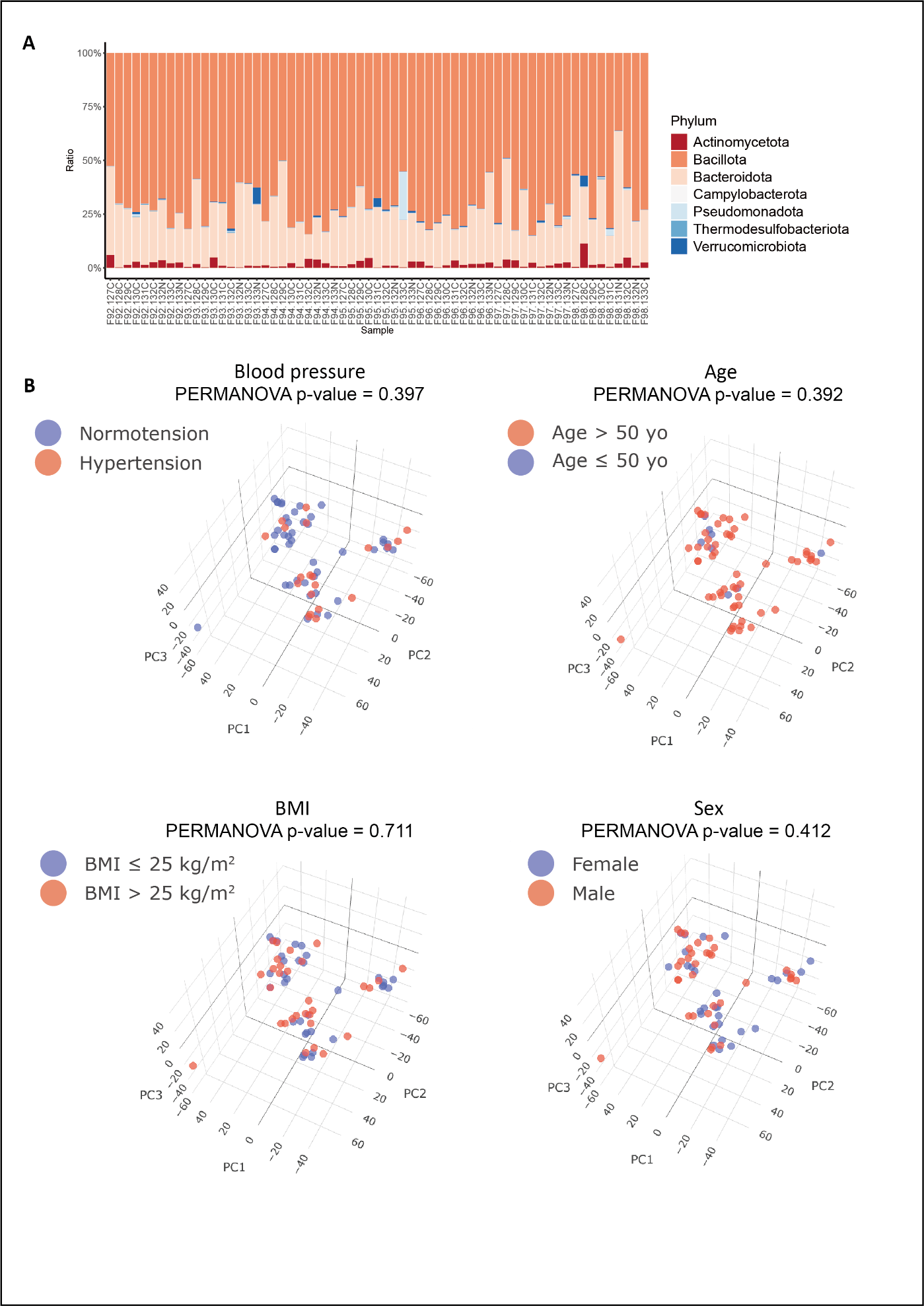
**

**Figure S1** | **Overview of microbial protein expression profiles in the VicGut cohort. A.** Distribution of microbial protein abundance at the phylum level. Proteins sourced from Bacillota and Bacteroidota constituted the majority of the total microbial protein abundance, with other phyla such as Campylobacteota detected in lower proportions, consistent with the typical composition of the human gut microbiome **B.** PCA plot illustrating the influence of traditional cardiovascular risk factors on microbial protein expression profiles. N=63.


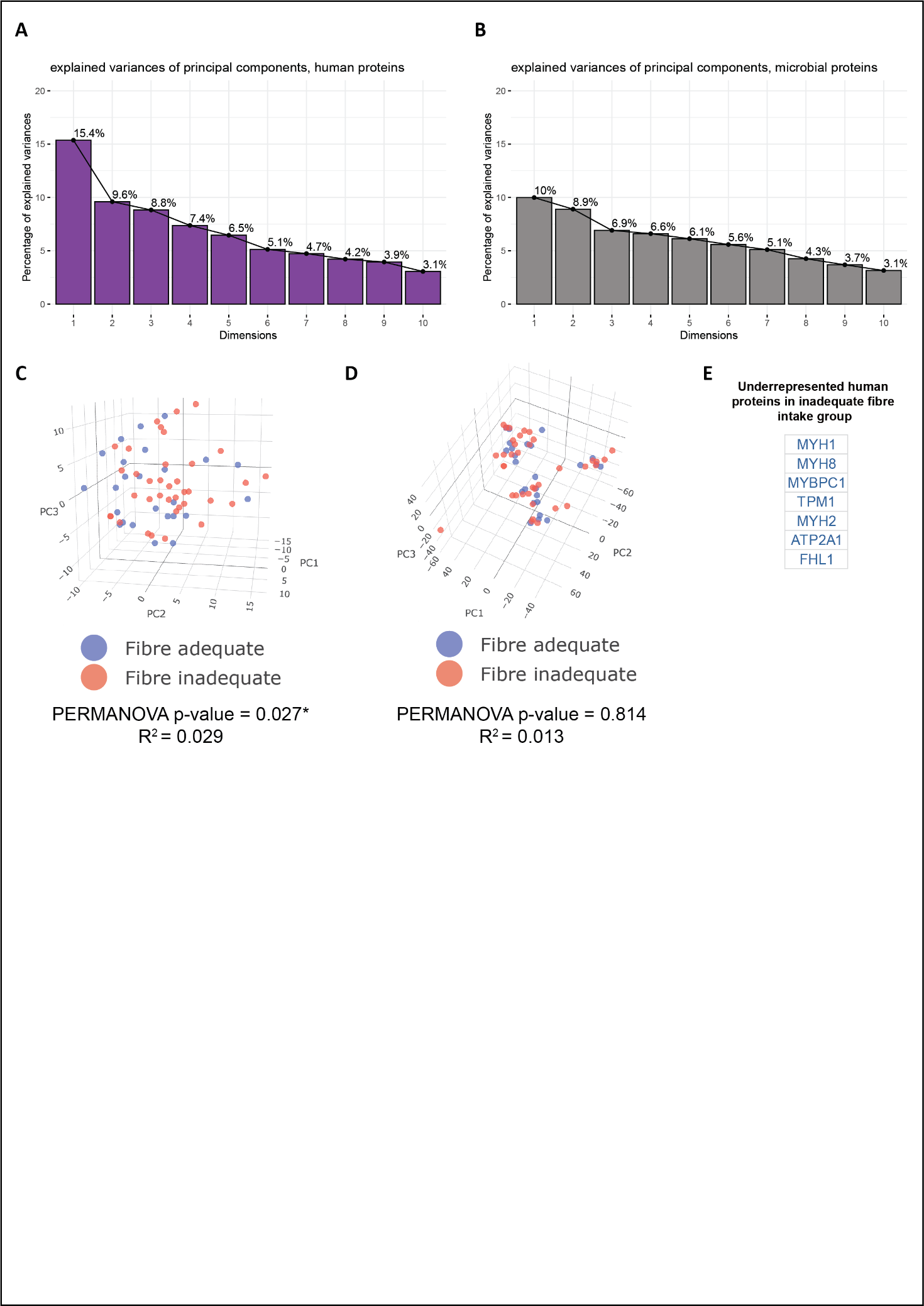


**Figure S2** | **Principal component analysis of gut lumen protein expression profiles.** Variance contribution of principal components for **A.** total human protein expression profile and **B.** total microbial protein expression profile. **C-D.** Principal component analysis of the influence of fiber intake on **C.** Human Gut Lumen Protein Expression Profile and **D.** Microbial Protein Expression Profile (one individual without fibre intake was removed). **E.** 7 underrepresented gut lumen human proteins in individuals without adequate fibre intake compared to individuals with adequate fibre intake. Total n=62 samples.


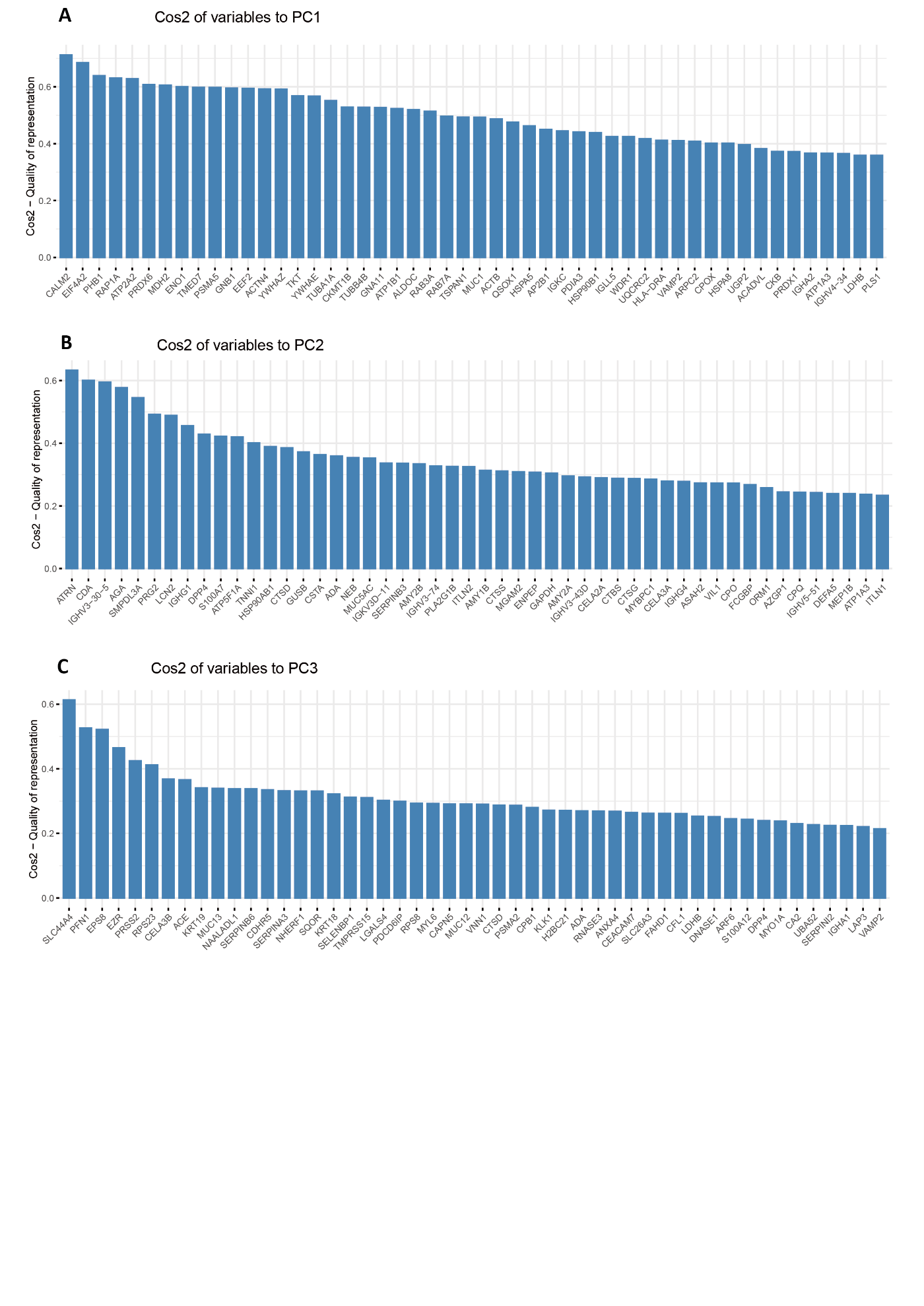


**Figure S3 | Top 50 contributing proteins for A.** Principal Component (PC) 1 **B.** PC2 **C.** PC3.


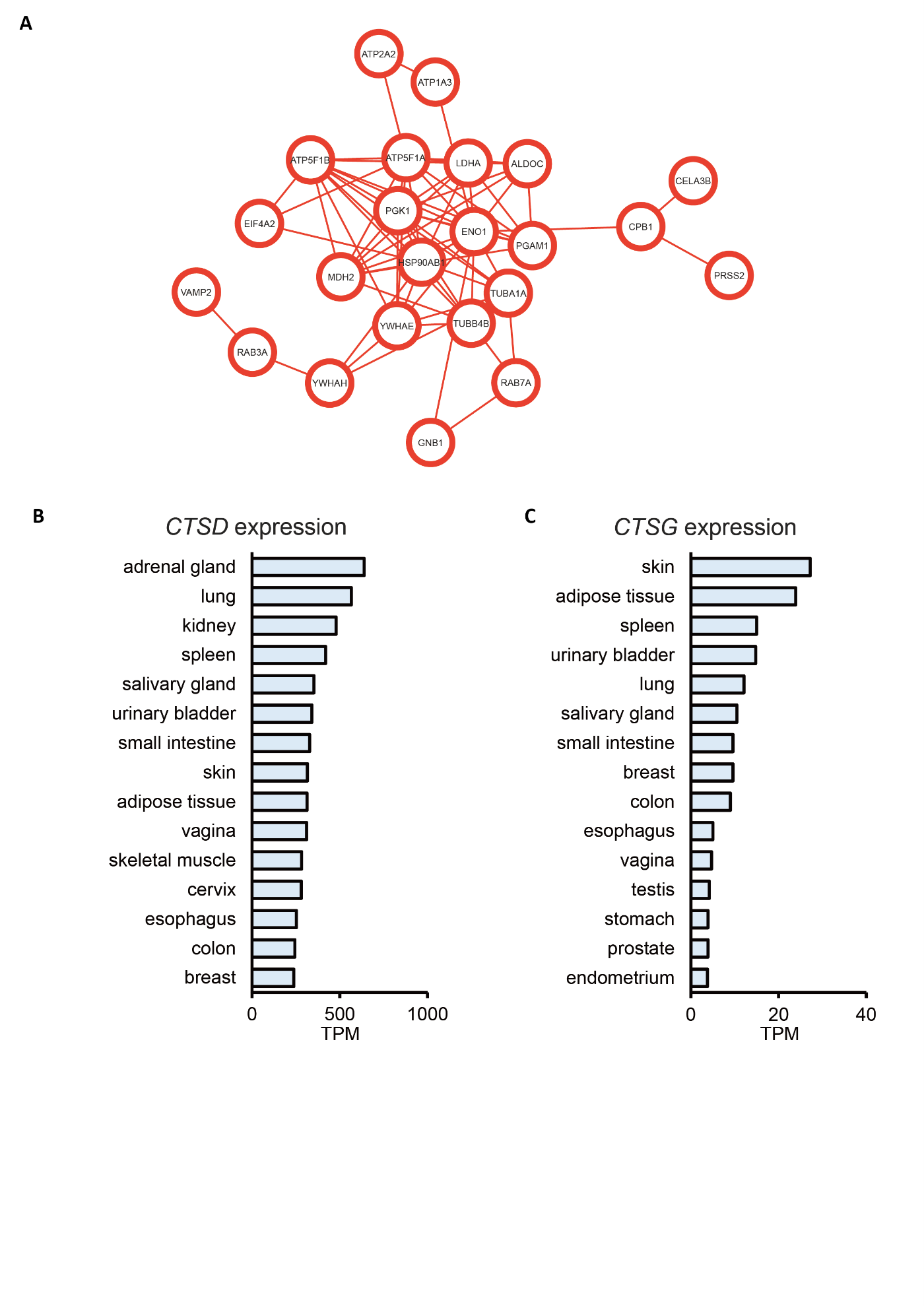


**Figure S4 | Protein-protein interaction (PPI) network of overrepresented gut lumen human proteins in the low-risk group and angiotensin converting enzymes expression profile in the human body. A.** A PPI network of overrepresented gut lumen human proteins in low-risk group.**B.-C.** Correlation analysis between gut lumen **B.** CTSD and **C.** CTSG protein levels and nighttime PP.


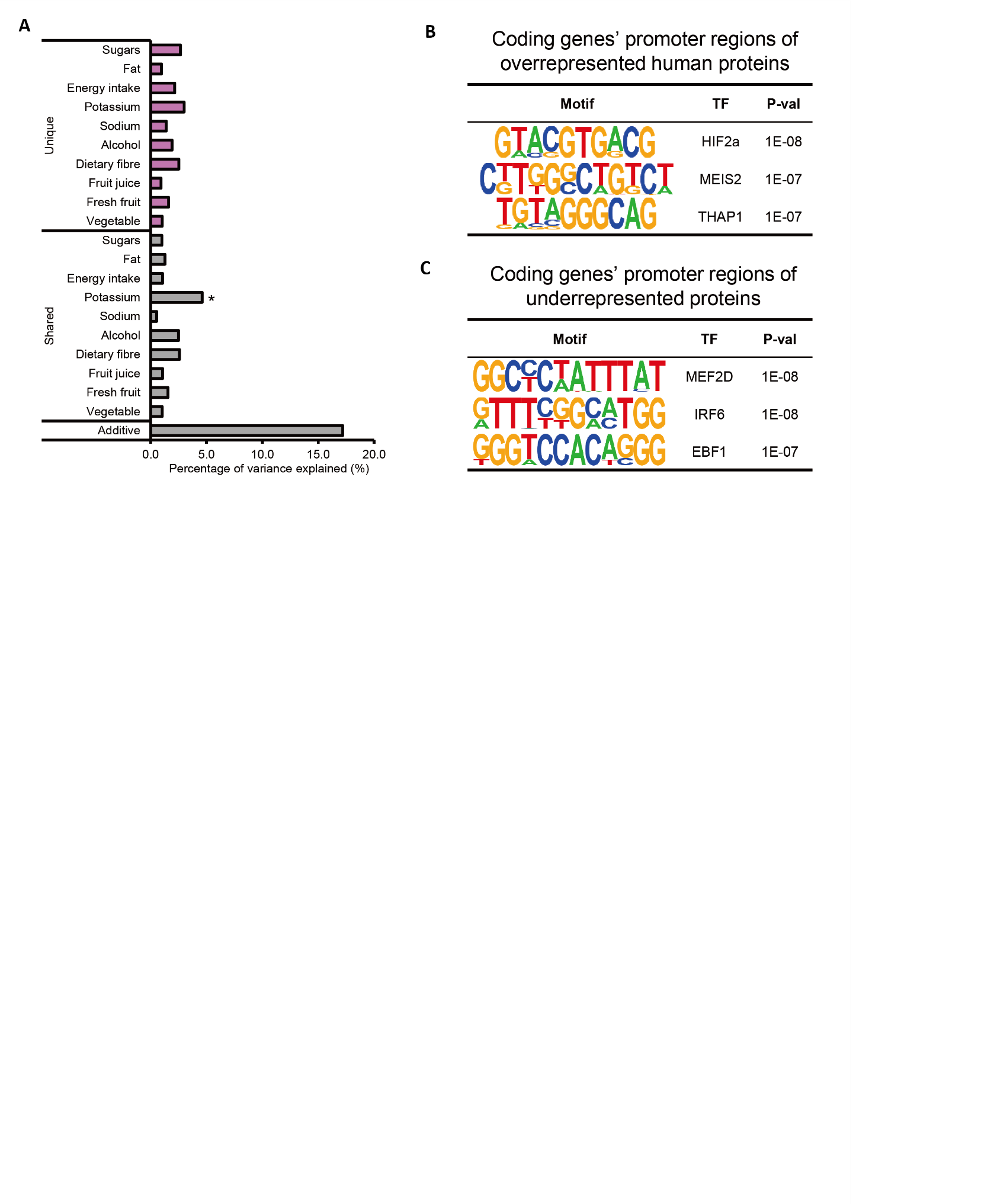


**Figure S5** | **Influence of regulators and dietary factors on gut lumen human protein expression. A.** Analysis depicting the extent to which various cardiovascular risk-related dietary factors account for variances in differentially expressed gut lumen human proteins. **B-C.** Motif search on the promoter regions of coding genes for **B.** overrepresented and **C.** underrepresented gut lumen human proteins in low-risk group. * stands for p < 0.05 by euclidean distance-based PERMANOVA test.


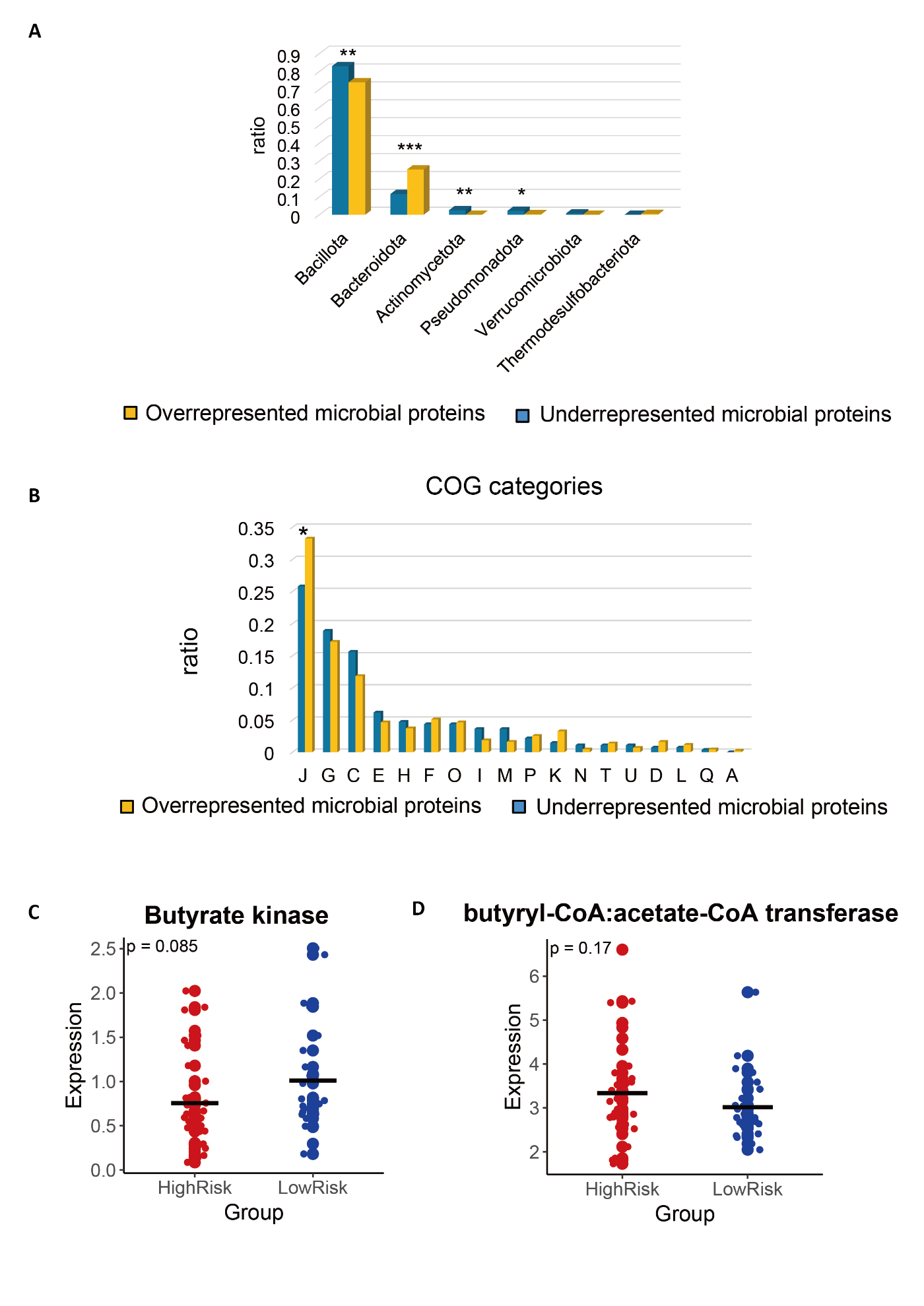


**Figure S6** | **Contrasts in the microbiome structure between low-risk and high-risk groups. A.** Distribution of differentially expressed microbial proteins at the phylum level between the two groups. *** denotes significance (p < 0.001), ** denotes significance (p < 0.01), and * denotes significance (p < 0.05) determined by Fisher’s exact test. **B.** Analysis of Clusters of Orthologous Groups (COG) categories for differentially expressed microbial proteins, revealing their broad biological functions, showing differences according to the low-risk group (i.e., overrepresentation means higher in low-risk group). COG categories listed are A: RNA processing and modification; C: Energy production and conversion; D: Cell cycle control and mitosis; E: Amino Acid metabolis and transport; F: Nucleotide metabolism and transport G: Carbohydrate metabolism and transport; H: Coenzyme metabolis; I: Lipid metabolism; J: Tranlsation; K: Transcription; L: Replication and repair; M: Cell wall/membrane/envelop biogenesis; N: Cell motility; O: Post-translational modification, protein turnover, chaperone functions; P: Inorganic ion transport and metabolism; Q: Secondary Structure; T: Signal Transduction; U: Intracellular trafficing and secretion. * denotes significance (p < 0.05) determined by Fisher's exact test. **C-D.** Comparison of the overall expression level of **C**. butyrate kinase and **D.** butyryl-CoA:acetate-CoA transferase in gut microbiota between low-risk group and high-risk group.


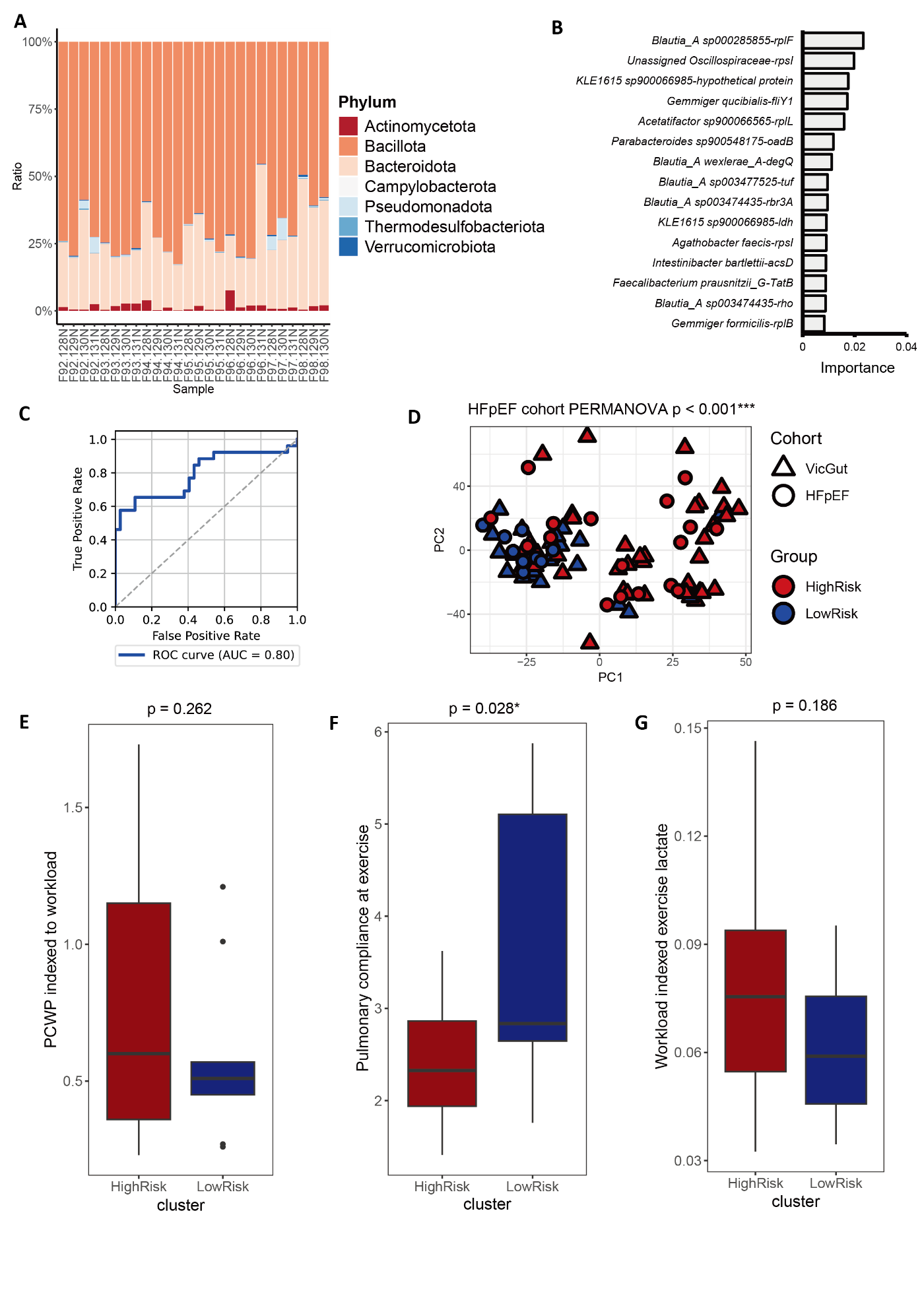


**Figure S7** | **Gut** **microbial biomass of HFpEF patients and machine learning model showing the relationship between microbial proteins and cardiovascular risk.** **A.** Phylum-level biomass abundance in HFpEF patients cohort. **B.** Presentation of the top 15 signature microbial proteins with the highest feature importance in the random forest model. **C.** Receiver Operating Characteristic (ROC) plot illustrating the results of 5-fold cross-validation of the constructed random forest model based on differentially expressed microbial proteins. **D.** PCA dimensionality reduction visualization of prediction results for Heart Failure with Preserved Ejection Fraction (HFpEF) patients using the trained microbial protein-based random forest model. **E-G.** Comparison of **E.** PCWP indexed to workload and **F.** Pulmonary compliance at exercise **G.** Workload indexed exercise lactate in HFpEF patients predicted as high-risk and low-risk. Sample size n=26.


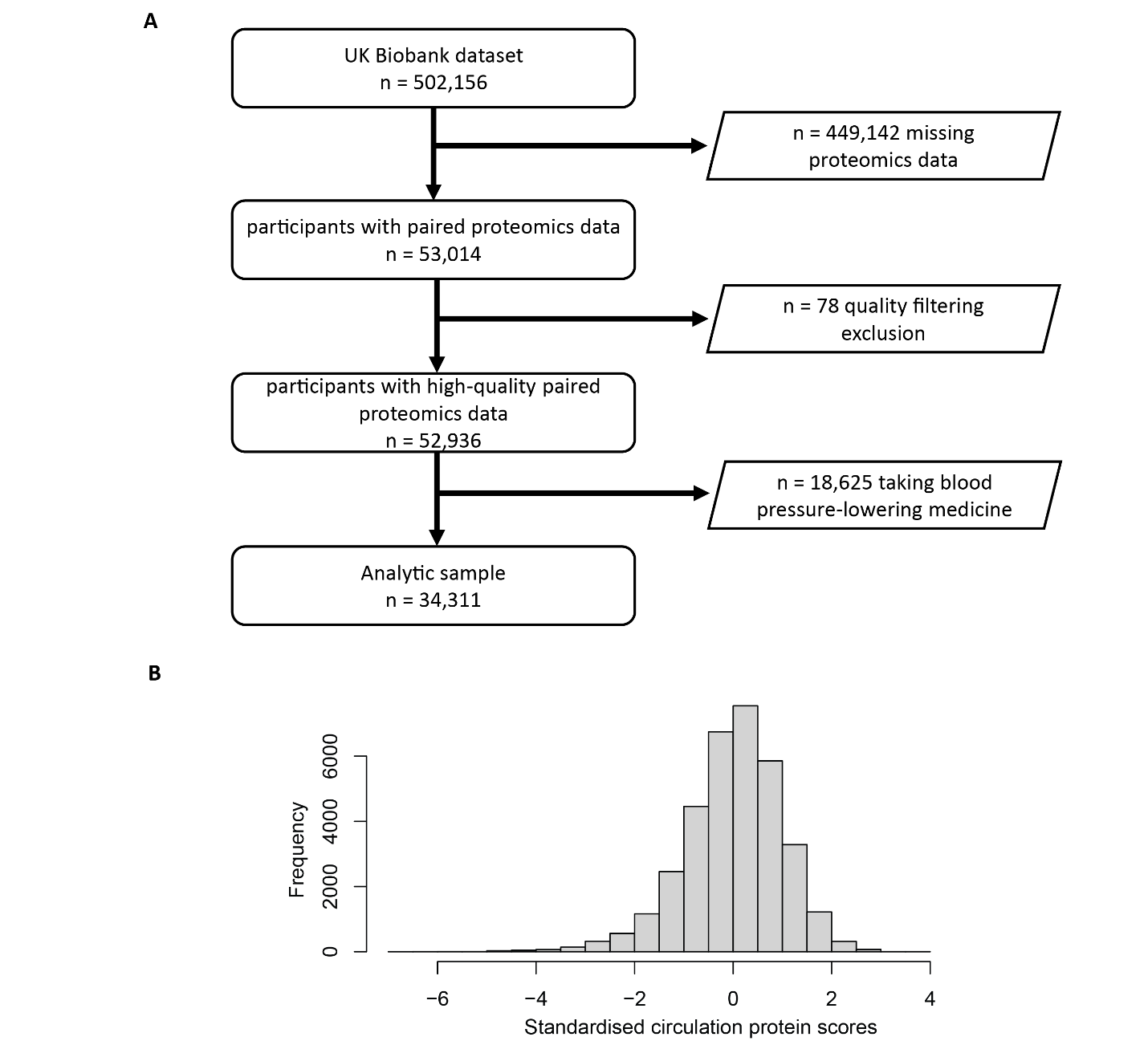


**Figure S8** | **Data processing for the UK Biobank dataset. A.** A flow chat showing the quality filtering process applied to the UK Biobank dataset. **B.** Distribution of standarised circulation protein scores among the analytic population.

**Supplementary Tables**

**Table S1** | **KEGG pathway overrepresentation analysis of the top 50 proteins contributing to PC1-PC3.**

|  | Term | Overlap | P-value | Adjusted P-value | Genes |
| --- | --- | --- | --- | --- | --- |
| PC1 | Insulin secretion | 5/86 | 2.38E-06 | 4.04E-04 | RAB3A;GNA11;ATP1A3;ATP1B1;VAMP2 |
|  | Salmonella infection | 6/249 | 3.52E-05 | 0.00102 | TUBA1A;ARPC2;TUBB4B;ACTB;RAB7A;HSP90B1 |
|  | Parkinson disease | 6/249 | 3.52E-05 | 0.00102 | PSMA5;TUBA1A;HSPA5;UQCRC2;TUBB4B;CALM2 |
|  | Thyroid hormone synthesis | 4/75 | 3.69E-05 | 0.00102 | HSPA5;ATP1A3;ATP1B1;HSP90B1 |
|  | Phagosome | 5/152 | 3.82E-05 | 0.00102 | TUBA1A;HLA-DRA;TUBB4B;ACTB;RAB7A |
| PC2 | Pancreatic secretion | 7/102 | 6.08E-09 | 4.80E-07 | CELA3A;CELA2A;AMY2A;AMY2B;PLA2G1B;AMY1B;ATP1A3 |
|  | Carbohydrate digestion and absorption | 5/47 | 1.13E-07 | 4.45E-06 | MGAM2;AMY2A;AMY2B;AMY1B;ATP1A3 |
|  | Starch and sucrose metabolism | 4/36 | 1.92E-06 | 5.05E-05 | MGAM2;AMY2A;AMY2B;AMY1B |
|  | Protein digestion and absorption | 5/103 | 5.79E-06 | 1.14E-04 | DPP4;CELA3A;CELA2A;MEP1B;ATP1A3 |
|  | Lysosome | 5/128 | 1.67E-05 | 2.64E-04 | CTSG;AGA;GUSB;CTSD;CTSS |
| PC3 | Pancreatic secretion | 5/102 | 5.52E-06 | 4.47E-04 | CELA3B;CPB1;CA2;PRSS2;SLC26A3 |
|  | Protein digestion and absorption | 4/103 | 1.27E-04 | 0.005158 | DPP4;CELA3B;CPB1;PRSS2 |
|  | Sulfur metabolism | 2/10 | 2.72E-04 | 0.007347 | SELENBP1;SQOR |
|  | Renin-angiotensin system | 2/23 | 0.001499 | 0.030345 | ACE;KLK1 |
|  | Coronavirus disease | 4/232 | 0.002675 | 0.043338 | ACE;RPS8;UBA52;RPS23 |

Legend: PC, principal component

**Table S2** | **Overrepresented gut lumen human proteins in Cluster 1 (low-risk group).**

| **Protein abbreviation** | **log_2_FC** | **FDR P-value** |
| --- | --- | --- |
| ATP1A3 | 1.79131 | 0.000101 |
| TUBA1A | 1.5918 | 0.000452 |
| TUBB4B | 1.447955 | 0.001527 |
| VAMP2 | 1.057992 | 0.000392 |
| HSP90AB1 | 0.99601 | 0.00032 |
| ATP5F1A | 0.99375 | 0.000149 |
| CPB1 | 0.975814 | 0.046371 |
| YWHAE | 0.955635 | 0.004743 |
| ALDOC | 0.941856 | 0.005294 |
| PRSS2 | 0.928581 | 0.038251 |
| ATP2A2 | 0.890075 | 0.002652 |
| LDHA | 0.87659 | 0.001547 |
| CELA3B | 0.829456 | 0.047283 |
| MDH2 | 0.783148 | 0.007788 |
| GNB1 | 0.780504 | 0.014401 |
| LDHB | 0.752448 | 0.002666 |
| RAB3A | 0.740326 | 0.004174 |
| ENO1 | 0.727981 | 0.011062 |
| ATP5F1B | 0.720595 | 0.047957 |
| PGAM1 | 0.710092 | 0.020673 |
| MYO1D | 0.68635 | 0.001055 |
| RAB7A | 0.677911 | 0.004828 |
| PGK1 | 0.658631 | 0.010552 |
| YWHAH | 0.631763 | 0.009324 |
| EIF4A2 | 0.599408 | 0.014401 |

Legend: FDR, false discovery rate; log_2_FC, log2 fold change

**Table S3** | **Underrepresented gut lumen human proteins in Cluster 1 (low-risk group).**

| **Protein abbreviation** | **log_2_FC** | **FDR P-value** |
| --- | --- | --- |
| IGKV2D-30 | -1.62129 | 4.32E-06 |
| IGHV3-30-5 | -1.21152 | 7.09E-05 |
| PDCD6IP | -1.16515 | 1.50E-06 |
| MYH7 | -1.12055 | 0.009185 |
| H2BC21 | -1.09034 | 0.00062 |
| MPO | -1.01709 | 0.000584 |
| CTSG | -1.00657 | 7.09E-05 |
| CA4 | -0.97885 | 0.000149 |
| ELANE | -0.93808 | 0.000247 |
| CTSD | -0.93557 | 1.45E-05 |
| ATRN | -0.93304 | 0.000101 |
| IGHA1 | -0.93275 | 0.001527 |
| LGALS4 | -0.91938 | 0.00062 |
| S100A12 | -0.90412 | 0.008079 |
| S100A8 | -0.88697 | 0.005832 |
| GP2 | -0.86442 | 0.013851 |
| LCN2 | -0.86012 | 0.010658 |
| EPX | -0.84861 | 0.006569 |
| ITLN2 | -0.8476 | 0.004769 |
| SLC44A4 | -0.83873 | 3.84E-05 |
| ANXA4 | -0.83604 | 0.00064 |
| RNASE3 | -0.81451 | 0.000335 |
| IGHV3-43D | -0.81286 | 0.00753 |
| IGHM | -0.80141 | 0.012847 |
| CELA3A | -0.80035 | 0.002652 |
| IGHG1 | -0.79769 | 0.020215 |
| FHL1 | -0.79023 | 0.022108 |
| LTF | -0.78926 | 0.006569 |
| UBA52 | -0.75199 | 0.011466 |
| PIGR | -0.75027 | 0.006569 |
| IGHV3-74 | -0.74041 | 4.19E-05 |
| IGKV3D-11 | -0.73047 | 0.004174 |
| PFN1 | -0.71158 | 0.004447 |
| PRG2 | -0.68587 | 0.00062 |
| APCS | -0.68078 | 0.020537 |
| RPS23 | -0.6779 | 0.000584 |
| ASAH2 | -0.65878 | 0.004174 |
| GAPDH | -0.61931 | 0.037249 |
| CEACAM7 | -0.61675 | 0.019375 |
| MUC13 | -0.59609 | 0.048447 |

Legend: FDR, false discovery rate; log2FC, log2 fold change

**Table S4** | **Pathway overrepresentation analysis of the overrepresented and underrepresented gut lumen human proteins.**

|  |  | **Term** | **Overlap** | **P-value** | **Adjusted P-value** | **Proteins** |
| --- | --- | --- | --- | --- | --- | --- |
| Overrepresented human proteins | KEGG | Glycolysis / Gluconeogenesis | 6/67 | 1.89E-10 | 1.89E-08 | LDHB;LDHA;PGAM1;PGK1;ALDOC;ENO1 |
|  |  | Pancreatic secretion | 5/102 | 1.53E-07 | 7.12E-06 | CELA3B;CPB1;ATP2A2;ATP1A3;PRSS2 |
|  |  | HIF-1 signaling pathway | 5/109 | 2.13E-07 | 7.12E-06 | LDHB;LDHA;PGK1;ALDOC;ENO1 |
|  | Wiki  Pathway | Glycolysis And Gluconeogenesis | 7/45 | 8.34E-14 | 5.42E-12 | LDHB;LDHA;MDH2;PGAM1;PGK1;ALDOC;ENO1 |
|  |  | Metabolic Epileptic Disorders | 7/92 | 1.55E-11 | 5.04E-10 | LDHB;LDHA;MDH2;PGAM1;PGK1;ALDOC;ENO1 |
|  |  | Glycolysis In Senescence | 4/11 | 6.23E-10 | 1.35E-08 | LDHA;PGK1;ALDOC;ENO1 |
| Underrepresented human proteins | KEGG | Asthma | 3/31 | 3.20E-05 | 0.001666 | PRG2;RNASE3;EPX |
|  |  | Neutrophil extracellular trap formation | 4/189 | 5.41E-04 | 0.014069 | H2BC21;CTSG;MPO;ELANE |
|  |  | Systemic lupus erythematosus | 3/135 | 0.002476 | 0.042911 | H2BC21;CTSG;ELANE |
|  | Wiki  Pathway | Conversion Of Angiotensinogen To Angiotensin II | 2/5 | 3.89E-05 | 0.001865 | CTSG;CTSD |
|  |  | Disturbed Pathways In Duchenne Muscular Dystrophy | 3/74 | 4.35E-04 | 0.010449 | GP2;MPO;ELANE |
|  |  | miRNA Role In Immune Response In Sepsis | 2/37 | 0.002485 | 0.03976 | LCN2;ELANE |

**Table S5** | **Overrepresented microbial proteins in low-risk group.**

| **Protein ID** | **Phylum** | **Class** | **Order** | **Family** | **Genus** | **Species** | **log_2_FC** | **FDR** |
| --- | --- | --- | --- | --- | --- | --- | --- | --- |
| MGYG000000213_01900 | Firmicutes_A | Clostridia | Lachnospirales | Lachnospiraceae | Blautia_A | Blautia_A sp003477525 | 1.913652 | 0.000809 |
| MGYG000002696_00008 | Firmicutes_A | Clostridia | Oscillospirales | Oscillospiraceae |  |  | 1.742544 | 2.02E-06 |
| MGYG000000002_01283 | Firmicutes_A | Clostridia | Lachnospirales | Lachnospiraceae | Blautia_A | Blautia_A faecis | 1.661855 | 1.55E-05 |
| MGYG000002312_02262 | Firmicutes_A | Clostridia | Lachnospirales | Lachnospiraceae | Blautia_A | Blautia_A sp000285855 | 1.59403 | 7.44E-08 |
| MGYG000001878_01907 | Bacteroidota | Bacteroidia | Bacteroidales | Tannerellaceae | Parabacteroides | Parabacteroides sp900548175 | 1.493158 | 6.77E-05 |
| MGYG000001189_01092 | Firmicutes_A | Clostridia | Lachnospirales | Lachnospiraceae | Blautia_A |  | 1.296744 | 0.000108 |
| MGYG000004797_01140 | Bacteroidota | Bacteroidia | Bacteroidales | Bacteroidaceae | Phocaeicola | Phocaeicola sartorii | 1.289169 | 0.000108 |
| MGYG000000028_01612 | Firmicutes_A | Clostridia | Lachnospirales | Lachnospiraceae | Anaerostipes | Anaerostipes hadrus_A | 1.279851 | 0.000195 |
| MGYG000000078_01673 | Firmicutes_A | Clostridia | Lachnospirales | Lachnospiraceae | Lachnospira | Lachnospira rogosae_A | 1.271242 | 0.000331 |
| MGYG000002478_00807 | Bacteroidota | Bacteroidia | Bacteroidales | Bacteroidaceae | Phocaeicola | Phocaeicola dorei | 1.253314 | 0.015198 |
| MGYG000002530_00144 | Firmicutes_A | Clostridia | Oscillospirales | Ruminococcaceae | Ruminiclostridium_E | Ruminiclostridium_E siraeum | 1.252808 | 0.000108 |
| MGYG000002478_03999 | Bacteroidota | Bacteroidia | Bacteroidales | Bacteroidaceae | Phocaeicola | Phocaeicola dorei | 1.247546 | 0.001648 |
| MGYG000002478_02059 | Bacteroidota | Bacteroidia | Bacteroidales | Bacteroidaceae | Phocaeicola | Phocaeicola dorei | 1.24224 | 0.000108 |
| MGYG000002478_01736 | Bacteroidota | Bacteroidia | Bacteroidales | Bacteroidaceae | Phocaeicola | Phocaeicola dorei | 1.224424 | 0.000187 |
| MGYG000004797_00999 | Bacteroidota | Bacteroidia | Bacteroidales | Bacteroidaceae | Phocaeicola | Phocaeicola sartorii | 1.192827 | 1.55E-05 |
| MGYG000003695_01274 | Firmicutes_A | Clostridia | Lachnospirales | Lachnospiraceae | Ruminococcus_A | Ruminococcus_A sp003011855 | 1.18628 | 0.00035 |
| MGYG000000136_02052 | Firmicutes_A | Clostridia | Lachnospirales | Lachnospiraceae | Agathobacter | Agathobacter sp000434275 | 1.180325 | 0.008681 |
| MGYG000000553_00676 | Bacteroidota | Bacteroidia | Bacteroidales | Bacteroidaceae | Prevotella | Prevotella sp900548535 | 1.171298 | 0.015324 |
| MGYG000002545_01138 | Firmicutes_A | Clostridia | Oscillospirales | Ruminococcaceae | Faecalibacterium | Faecalibacterium prausnitzii_G | 1.169657 | 0.000108 |
| MGYG000002040_01716 | Firmicutes_A | Clostridia | Oscillospirales | Ruminococcaceae | Faecalibacterium | Faecalibacterium sp900758465 | 1.163647 | 0.000207 |
| MGYG000002670_00617 | Firmicutes_A | Clostridia | Lachnospirales | Lachnospiraceae | Agathobacter | Agathobacter sp900546625 | 1.161948 | 0.000354 |
| MGYG000001189_00858 | Firmicutes_A | Clostridia | Lachnospirales | Lachnospiraceae | Blautia_A |  | 1.152992 | 0.001643 |
| MGYG000000193_03112 | Firmicutes_A | Clostridia | Lachnospirales | Lachnospiraceae | KLE1615 | KLE1615 sp900066985 | 1.136126 | 3.28E-06 |
| MGYG000000039_01710 | Firmicutes_A | Clostridia | Oscillospirales | Ruminococcaceae | Faecalibacterium | Faecalibacterium prausnitzii_H | 1.133631 | 0.000711 |
| MGYG000002619_00002 | Firmicutes_A | Clostridia | Oscillospirales | Ruminococcaceae | Faecalibacterium | Faecalibacterium prausnitzii_J | 1.131521 | 6.60E-05 |
| MGYG000000312_01484 | Firmicutes_A | Clostridia | Lachnospirales | Lachnospiraceae | Eisenbergiella | Eisenbergiella sp900539715 | 1.120595 | 0.000422 |
| MGYG000000002_01223 | Firmicutes_A | Clostridia | Lachnospirales | Lachnospiraceae | Blautia_A | Blautia_A faecis | 1.116412 | 0.000979 |
| MGYG000000486_01426 | Firmicutes_C | Negativicutes | Acidaminococcales | Acidaminococcaceae | Phascolarctobacterium_A | Phascolarctobacterium_A sp900770955 | 1.110548 | 0.016091 |
| MGYG000001346_01345 | Bacteroidota | Bacteroidia | Bacteroidales | Bacteroidaceae | Bacteroides | Bacteroides uniformis | 1.10932 | 0.000333 |
| MGYG000003694_02447 | Firmicutes_A | Clostridia | Lachnospirales | Lachnospiraceae | Agathobacter | Agathobacter faecis | 1.103106 | 0.000677 |
| MGYG000003702_00991 | Firmicutes_A | Clostridia | Lachnospirales | Lachnospiraceae | Eisenbergiella | Eisenbergiella sp900066775 | 1.102445 | 0.000462 |
| MGYG000000251_01802 | Firmicutes_A | Clostridia | Lachnospirales | Lachnospiraceae | Fusicatenibacter | Fusicatenibacter saccharivorans | 1.101988 | 0.000817 |
| MGYG000002478_01537 | Bacteroidota | Bacteroidia | Bacteroidales | Bacteroidaceae | Phocaeicola | Phocaeicola dorei | 1.101398 | 2.16E-05 |
| MGYG000004296_00104 | Firmicutes_A | Clostridia | Lachnospirales | Lachnospiraceae | Ruminococcus_A | Ruminococcus_A faecicola | 1.098829 | 0.003116 |
| MGYG000000142_01261 | Firmicutes_A | Clostridia | Lachnospirales | Lachnospiraceae | Blautia_A | Blautia_A massiliensis | 1.09764 | 0.003783 |
| MGYG000003702_00216 | Firmicutes_A | Clostridia | Lachnospirales | Lachnospiraceae | Eisenbergiella | Eisenbergiella sp900066775 | 1.096347 | 0.001653 |
| MGYG000000054_03350 | Bacteroidota | Bacteroidia | Bacteroidales | Bacteroidaceae | Bacteroides | Bacteroides acidifaciens | 1.091453 | 0.000909 |
| MGYG000002438_02028 | Bacteroidota | Bacteroidia | Bacteroidales | Tannerellaceae | Parabacteroides | Parabacteroides distasonis | 1.090459 | 0.027522 |
| MGYG000000184_02369 | Firmicutes_A | Clostridia | Lachnospirales | Lachnospiraceae | Blautia_A | Blautia_A luti | 1.087475 | 0.000195 |
| MGYG000000022_01083 | Firmicutes_A | Clostridia | Oscillospirales | Ruminococcaceae | Faecalibacterium | Faecalibacterium prausnitzii_C | 1.087091 | 0.000337 |
| MGYG000002545_01667 | Firmicutes_A | Clostridia | Oscillospirales | Ruminococcaceae | Faecalibacterium | Faecalibacterium prausnitzii_G | 1.083011 | 0.0002 |
| MGYG000002298_02301 | Firmicutes_A | Clostridia | Lachnospirales | Lachnospiraceae | Blautia_A | Blautia_A sp000436615 | 1.082579 | 1.55E-05 |
| MGYG000002560_00339 | Bacteroidota | Bacteroidia | Bacteroidales | Bacteroidaceae | Phocaeicola | Phocaeicola sp902388365 | 1.07761 | 0.000422 |
| MGYG000004763_00463 | Bacteroidota | Bacteroidia | Bacteroidales | Bacteroidaceae | CAG-462 | CAG-462 sp900291465 | 1.077275 | 0.000195 |
| MGYG000002040_01818 | Firmicutes_A | Clostridia | Oscillospirales | Ruminococcaceae | Faecalibacterium | Faecalibacterium sp900758465 | 1.071894 | 2.10E-05 |
| MGYG000001689_04950 | Firmicutes_A | Clostridia | Lachnospirales | Lachnospiraceae | Blautia | Blautia coccoides | 1.062772 | 8.58E-06 |
| MGYG000002545_01542 | Firmicutes_A | Clostridia | Oscillospirales | Ruminococcaceae | Faecalibacterium | Faecalibacterium prausnitzii_G | 1.062567 | 0.001217 |
| MGYG000002478_02020 | Bacteroidota | Bacteroidia | Bacteroidales | Bacteroidaceae | Phocaeicola | Phocaeicola dorei | 1.061321 | 0.000479 |
| MGYG000000002_00872 | Firmicutes_A | Clostridia | Lachnospirales | Lachnospiraceae | Blautia_A | Blautia_A faecis | 1.057335 | 0.011395 |
| MGYG000002272_00024 | Firmicutes_A | Clostridia | Oscillospirales | Ruminococcaceae | Faecalibacterium | Faecalibacterium prausnitzii_D | 1.05034 | 0.003698 |
| MGYG000003899_00250 | Firmicutes_A | Clostridia | Oscillospirales | Ruminococcaceae | Faecalibacterium | Faecalibacterium sp900539945 | 1.031711 | 0.000161 |
| MGYG000002271_02178 | Firmicutes_A | Clostridia | Lachnospirales | Lachnospiraceae | GCA-900066135 | GCA-900066135 sp900066135 | 1.030472 | 0.004114 |
| MGYG000000216_03214 | Firmicutes_A | Clostridia | Lachnospirales | Lachnospiraceae | Blautia_A | Blautia_A sp003474435 | 1.030407 | 0.000743 |
| MGYG000000142_02056 | Firmicutes_A | Clostridia | Lachnospirales | Lachnospiraceae | Blautia_A | Blautia_A massiliensis | 1.01813 | 0.001173 |
| MGYG000000078_01638 | Firmicutes_A | Clostridia | Lachnospirales | Lachnospiraceae | Lachnospira | Lachnospira rogosae_A | 1.008764 | 0.001217 |
| MGYG000001346_00697 | Bacteroidota | Bacteroidia | Bacteroidales | Bacteroidaceae | Bacteroides | Bacteroides uniformis | 0.997299 | 0.001943 |
| MGYG000000251_00729 | Firmicutes_A | Clostridia | Lachnospirales | Lachnospiraceae | Fusicatenibacter | Fusicatenibacter saccharivorans | 0.996008 | 0.000691 |
| MGYG000000262_00972 | Firmicutes_A | Clostridia | Lachnospirales | Lachnospiraceae | Anaerobutyricum | Anaerobutyricum hallii | 0.993279 | 0.001512 |
| MGYG000000262_01525 | Firmicutes_A | Clostridia | Lachnospirales | Lachnospiraceae | Anaerobutyricum | Anaerobutyricum hallii | 0.991395 | 0.001883 |
| MGYG000002492_01818 | Firmicutes_A | Clostridia | Lachnospirales | Lachnospiraceae | Agathobacter | Agathobacter rectalis | 0.990841 | 0.000108 |
| MGYG000000140_01327 | Firmicutes_A | Clostridia | Lachnospirales | Lachnospiraceae | UMGS1375 | UMGS1375 sp900066615 | 0.990626 | 0.008928 |
| MGYG000002478_01373 | Bacteroidota | Bacteroidia | Bacteroidales | Bacteroidaceae | Phocaeicola | Phocaeicola dorei | 0.99029 | 0.000691 |
| MGYG000000028_00942 | Firmicutes_A | Clostridia | Lachnospirales | Lachnospiraceae | Anaerostipes | Anaerostipes hadrus_A | 0.990281 | 0.003698 |
| MGYG000002478_01940 | Bacteroidota | Bacteroidia | Bacteroidales | Bacteroidaceae | Phocaeicola | Phocaeicola dorei | 0.990148 | 0.023455 |
| MGYG000000193_01436 | Firmicutes_A | Clostridia | Lachnospirales | Lachnospiraceae | KLE1615 | KLE1615 sp900066985 | 0.981824 | 0.000897 |
| MGYG000000193_02351 | Firmicutes_A | Clostridia | Lachnospirales | Lachnospiraceae | KLE1615 | KLE1615 sp900066985 | 0.975473 | 0.000747 |
| MGYG000000031_01564 | Firmicutes_A | Clostridia | Lachnospirales | Lachnospiraceae | Blautia_A | Blautia_A sp900066205 | 0.97367 | 6.77E-05 |
| MGYG000000142_01835 | Firmicutes_A | Clostridia | Lachnospirales | Lachnospiraceae | Blautia_A | Blautia_A massiliensis | 0.971613 | 0.022996 |
| MGYG000002478_00641 | Bacteroidota | Bacteroidia | Bacteroidales | Bacteroidaceae | Phocaeicola | Phocaeicola dorei | 0.969585 | 0.000529 |
| MGYG000000217_01479 | Firmicutes_A | Clostridia | Lachnospirales | Lachnospiraceae | Acetatifactor | Acetatifactor sp900066565 | 0.968525 | 0.000422 |
| MGYG000000250_00221 | Firmicutes_A | Clostridia | Lachnospirales | Lachnospiraceae | TF01-11 | TF01-11 sp001414325 | 0.966868 | 0.001883 |
| MGYG000000140_01891 | Firmicutes_A | Clostridia | Lachnospirales | Lachnospiraceae | UMGS1375 | UMGS1375 sp900066615 | 0.964908 | 0.003934 |
| MGYG000001346_02584 | Bacteroidota | Bacteroidia | Bacteroidales | Bacteroidaceae | Bacteroides | Bacteroides uniformis | 0.962215 | 0.000881 |
| MGYG000002528_00040 | Firmicutes_A | Clostridia | Lachnospirales | Lachnospiraceae | Anaerostipes | Anaerostipes hadrus | 0.962213 | 0.004156 |
| MGYG000001631_00039 | Desulfobacterota | Desulfovibrionia | Desulfovibrionales | Desulfovibrionaceae | Bilophila | Bilophila sp900553145 | 0.957739 | 0.001162 |
| MGYG000000078_01623 | Firmicutes_A | Clostridia | Lachnospirales | Lachnospiraceae | Lachnospira | Lachnospira rogosae_A | 0.953778 | 0.001883 |
| MGYG000002492_00336 | Firmicutes_A | Clostridia | Lachnospirales | Lachnospiraceae | Agathobacter | Agathobacter rectalis | 0.952296 | 0.004726 |
| MGYG000002478_00803 | Bacteroidota | Bacteroidia | Bacteroidales | Bacteroidaceae | Phocaeicola | Phocaeicola dorei | 0.95202 | 0.001125 |
| MGYG000001364_02728 | Bacteroidota | Bacteroidia | Bacteroidales | Bacteroidaceae | Phocaeicola | Phocaeicola plebeius | 0.950793 | 0.021399 |
| MGYG000002545_01181 | Firmicutes_A | Clostridia | Oscillospirales | Ruminococcaceae | Faecalibacterium | Faecalibacterium prausnitzii_G | 0.943863 | 0.002957 |
| MGYG000003899_00614 | Firmicutes_A | Clostridia | Oscillospirales | Ruminococcaceae | Faecalibacterium | Faecalibacterium sp900539945 | 0.941469 | 0.000354 |
| MGYG000000213_01134 | Firmicutes_A | Clostridia | Lachnospirales | Lachnospiraceae | Blautia_A | Blautia_A sp003477525 | 0.938261 | 0.002532 |
| MGYG000001255_00159 | Firmicutes_A | Clostridia | Oscillospirales | Ruminococcaceae | Faecalibacterium | Faecalibacterium prausnitzii_F | 0.936027 | 0.012022 |
| MGYG000001631_00051 | Desulfobacterota | Desulfovibrionia | Desulfovibrionales | Desulfovibrionaceae | Bilophila | Bilophila sp900553145 | 0.935247 | 0.000804 |
| MGYG000003899_00433 | Firmicutes_A | Clostridia | Oscillospirales | Ruminococcaceae | Faecalibacterium | Faecalibacterium sp900539945 | 0.934321 | 0.009072 |
| MGYG000001302.1_02054 | Bacteroidota | Bacteroidia | Bacteroidales | Rikenellaceae | Alistipes | Alistipes putredinis | 0.934203 | 0.007451 |
| MGYG000002478_01971 | Bacteroidota | Bacteroidia | Bacteroidales | Bacteroidaceae | Phocaeicola | Phocaeicola dorei | 0.933025 | 0.000298 |
| MGYG000002670_00541 | Firmicutes_A | Clostridia | Lachnospirales | Lachnospiraceae | Agathobacter | Agathobacter sp900546625 | 0.9306 | 0.001883 |
| MGYG000002492_00339 | Firmicutes_A | Clostridia | Lachnospirales | Lachnospiraceae | Agathobacter | Agathobacter rectalis | 0.926965 | 0.00456 |
| MGYG000000217_00569 | Firmicutes_A | Clostridia | Lachnospirales | Lachnospiraceae | Acetatifactor | Acetatifactor sp900066565 | 0.92564 | 0.001051 |
| MGYG000002478_02960 | Bacteroidota | Bacteroidia | Bacteroidales | Bacteroidaceae | Phocaeicola | Phocaeicola dorei | 0.923121 | 0.00546 |
| MGYG000000193_01005 | Firmicutes_A | Clostridia | Lachnospirales | Lachnospiraceae | KLE1615 | KLE1615 sp900066985 | 0.922659 | 0.009833 |
| MGYG000000022_00097 | Firmicutes_A | Clostridia | Oscillospirales | Ruminococcaceae | Faecalibacterium | Faecalibacterium prausnitzii_C | 0.919313 | 0.040999 |
| MGYG000002545_02469 | Firmicutes_A | Clostridia | Oscillospirales | Ruminococcaceae | Faecalibacterium | Faecalibacterium prausnitzii_G | 0.91689 | 0.001274 |
| MGYG000000078_01654 | Firmicutes_A | Clostridia | Lachnospirales | Lachnospiraceae | Lachnospira | Lachnospira rogosae_A | 0.913428 | 0.000126 |
| MGYG000002545_01920 | Firmicutes_A | Clostridia | Oscillospirales | Ruminococcaceae | Faecalibacterium | Faecalibacterium prausnitzii_G | 0.91248 | 0.001065 |
| MGYG000000209_01835 | Firmicutes_A | Clostridia | Lachnospirales | Lachnospiraceae | Eubacterium_F | Eubacterium_F sp003491505 | 0.905649 | 0.000296 |
| MGYG000000217_01255 | Firmicutes_A | Clostridia | Lachnospirales | Lachnospiraceae | Acetatifactor | Acetatifactor sp900066565 | 0.905495 | 0.010073 |
| MGYG000002492_00114 | Firmicutes_A | Clostridia | Lachnospirales | Lachnospiraceae | Agathobacter | Agathobacter rectalis | 0.904324 | 0.010436 |
| MGYG000001346_02120 | Bacteroidota | Bacteroidia | Bacteroidales | Bacteroidaceae | Bacteroides | Bacteroides uniformis | 0.903013 | 0.001274 |
| MGYG000002670_00405 | Firmicutes_A | Clostridia | Lachnospirales | Lachnospiraceae | Agathobacter | Agathobacter sp900546625 | 0.901015 | 0.00076 |
| MGYG000000078_00791 | Firmicutes_A | Clostridia | Lachnospirales | Lachnospiraceae | Lachnospira | Lachnospira rogosae_A | 0.898207 | 0.002282 |
| MGYG000001456.1_01341 | Firmicutes_A | Clostridia | Lachnospirales | Lachnospiraceae | Eubacterium_I | Eubacterium_I ramulus | 0.894043 | 0.012408 |
| MGYG000000142_00716 | Firmicutes_A | Clostridia | Lachnospirales | Lachnospiraceae | Blautia_A | Blautia_A massiliensis | 0.892973 | 0.008095 |
| MGYG000001689_04434 | Firmicutes_A | Clostridia | Lachnospirales | Lachnospiraceae | Blautia | Blautia coccoides | 0.89237 | 0.000418 |
| MGYG000000271_03296 | Firmicutes_A | Clostridia | Lachnospirales | Lachnospiraceae | Roseburia | Roseburia sp900552665 | 0.888381 | 0.000252 |
| MGYG000001346_02846 | Bacteroidota | Bacteroidia | Bacteroidales | Bacteroidaceae | Bacteroides | Bacteroides uniformis | 0.887501 | 0.002149 |
| MGYG000002561_02623 | Bacteroidota | Bacteroidia | Bacteroidales | Bacteroidaceae | Bacteroides | Bacteroides sp902388495 | 0.88727 | 0.001666 |
| MGYG000002492_01574 | Firmicutes_A | Clostridia | Lachnospirales | Lachnospiraceae | Agathobacter | Agathobacter rectalis | 0.881941 | 0.008674 |
| MGYG000002478_00548 | Bacteroidota | Bacteroidia | Bacteroidales | Bacteroidaceae | Phocaeicola | Phocaeicola dorei | 0.880548 | 0.001462 |
| MGYG000000002_02866 | Firmicutes_A | Clostridia | Lachnospirales | Lachnospiraceae | Blautia_A | Blautia_A faecis | 0.879907 | 0.010468 |
| MGYG000001346_03404 | Bacteroidota | Bacteroidia | Bacteroidales | Bacteroidaceae | Bacteroides | Bacteroides uniformis | 0.873692 | 0.001913 |
| MGYG000002492_00629 | Firmicutes_A | Clostridia | Lachnospirales | Lachnospiraceae | Agathobacter | Agathobacter rectalis | 0.872822 | 0.009022 |
| MGYG000004296_00911 | Firmicutes_A | Clostridia | Lachnospirales | Lachnospiraceae | Ruminococcus_A | Ruminococcus_A faecicola | 0.87021 | 0.000797 |
| MGYG000001346_01598 | Bacteroidota | Bacteroidia | Bacteroidales | Bacteroidaceae | Bacteroides | Bacteroides uniformis | 0.864281 | 0.011405 |
| MGYG000002641_01969 | Firmicutes_A | Clostridia | Oscillospirales | Ruminococcaceae | Faecalibacterium | Faecalibacterium sp003449675 | 0.864095 | 0.006878 |
| MGYG000000262_00556 | Firmicutes_A | Clostridia | Lachnospirales | Lachnospiraceae | Anaerobutyricum | Anaerobutyricum hallii | 0.862567 | 0.010212 |
| MGYG000001238_00450 | Firmicutes_A | Clostridia | Oscillospirales | Oscillospiraceae | Evtepia | Evtepia sp900758955 | 0.861978 | 0.004709 |
| MGYG000000217_01428 | Firmicutes_A | Clostridia | Lachnospirales | Lachnospiraceae | Acetatifactor | Acetatifactor sp900066565 | 0.861607 | 0.001195 |
| MGYG000002492_01777 | Firmicutes_A | Clostridia | Lachnospirales | Lachnospiraceae | Agathobacter | Agathobacter rectalis | 0.859538 | 0.003617 |
| MGYG000001682_02138 | Bacteroidota | Bacteroidia | Bacteroidales | Muribaculaceae | Paramuribaculum | Paramuribaculum sp900760855 | 0.859487 | 0.002413 |
| MGYG000002478_02043 | Bacteroidota | Bacteroidia | Bacteroidales | Bacteroidaceae | Phocaeicola | Phocaeicola dorei | 0.856545 | 0.017864 |
| MGYG000002478_01929 | Bacteroidota | Bacteroidia | Bacteroidales | Bacteroidaceae | Phocaeicola | Phocaeicola dorei | 0.85506 | 0.005811 |
| MGYG000002492_01434 | Firmicutes_A | Clostridia | Lachnospirales | Lachnospiraceae | Agathobacter | Agathobacter rectalis | 0.854491 | 0.006208 |
| MGYG000000045_00965 | Firmicutes | Bacilli | Erysipelotrichales | Erysipelatoclostridiaceae | Faecalibacillus | Faecalibacillus intestinalis | 0.854144 | 0.004983 |
| MGYG000002992_01382 | Firmicutes_A | Clostridia | Lachnospirales | Lachnospiraceae | Dorea_A | Dorea_A sp900550865 | 0.852926 | 0.002532 |
| MGYG000002545_01140 | Firmicutes_A | Clostridia | Oscillospirales | Ruminococcaceae | Faecalibacterium | Faecalibacterium prausnitzii_G | 0.851967 | 0.014971 |
| MGYG000000045_02136 | Firmicutes | Bacilli | Erysipelotrichales | Erysipelatoclostridiaceae | Faecalibacillus | Faecalibacillus intestinalis | 0.84923 | 0.00194 |
| MGYG000000251_01917 | Firmicutes_A | Clostridia | Lachnospirales | Lachnospiraceae | Fusicatenibacter | Fusicatenibacter saccharivorans | 0.848481 | 0.001051 |
| MGYG000002492_00376 | Firmicutes_A | Clostridia | Lachnospirales | Lachnospiraceae | Agathobacter | Agathobacter rectalis | 0.84792 | 0.020539 |
| MGYG000001306_02660 | Bacteroidota | Bacteroidia | Bacteroidales | Bacteroidaceae | Phocaeicola | Phocaeicola coprocola | 0.847425 | 0.005003 |
| MGYG000001346_02076 | Bacteroidota | Bacteroidia | Bacteroidales | Bacteroidaceae | Bacteroides | Bacteroides uniformis | 0.844804 | 0.002926 |
| MGYG000002293_00919 | Bacteroidota | Bacteroidia | Bacteroidales | Bacteroidaceae | Prevotella | Prevotella sp900557255 | 0.844386 | 0.022382 |
| MGYG000000002_01946 | Firmicutes_A | Clostridia | Lachnospirales | Lachnospiraceae | Blautia_A | Blautia_A faecis | 0.843424 | 0.001883 |
| MGYG000002545_00526 | Firmicutes_A | Clostridia | Oscillospirales | Ruminococcaceae | Faecalibacterium | Faecalibacterium prausnitzii_G | 0.843127 | 0.002713 |
| MGYG000004489_00389 | Firmicutes_A | Clostridia | Oscillospirales | Oscillospiraceae | ER4 | ER4 sp900552015 | 0.84187 | 0.016808 |
| MGYG000000213_00744 | Firmicutes_A | Clostridia | Lachnospirales | Lachnospiraceae | Blautia_A | Blautia_A sp003477525 | 0.841539 | 0.000204 |
| MGYG000002528_02153 | Firmicutes_A | Clostridia | Lachnospirales | Lachnospiraceae | Anaerostipes | Anaerostipes hadrus | 0.840042 | 0.03832 |
| MGYG000001626_02098 | Firmicutes_C | Negativicutes | Acidaminococcales | Acidaminococcaceae | Phascolarctobacterium | Phascolarctobacterium sp900544795 | 0.83738 | 0.028173 |
| MGYG000002293_00486 | Bacteroidota | Bacteroidia | Bacteroidales | Bacteroidaceae | Prevotella | Prevotella sp900557255 | 0.834211 | 0.032557 |
| MGYG000000142_02503 | Firmicutes_A | Clostridia | Lachnospirales | Lachnospiraceae | Blautia_A | Blautia_A massiliensis | 0.832448 | 0.018853 |
| MGYG000000271_03364 | Firmicutes_A | Clostridia | Lachnospirales | Lachnospiraceae | Roseburia | Roseburia sp900552665 | 0.832269 | 0.000298 |
| MGYG000001306_02359 | Bacteroidota | Bacteroidia | Bacteroidales | Bacteroidaceae | Phocaeicola | Phocaeicola coprocola | 0.830968 | 0.002375 |
| MGYG000000217_02796 | Firmicutes_A | Clostridia | Lachnospirales | Lachnospiraceae | Acetatifactor | Acetatifactor sp900066565 | 0.82872 | 0.021399 |
| MGYG000000142_01978 | Firmicutes_A | Clostridia | Lachnospirales | Lachnospiraceae | Blautia_A | Blautia_A massiliensis | 0.827379 | 0.003918 |
| MGYG000000193_02644 | Firmicutes_A | Clostridia | Lachnospirales | Lachnospiraceae | KLE1615 | KLE1615 sp900066985 | 0.827199 | 0.002128 |
| MGYG000000028_01611 | Firmicutes_A | Clostridia | Lachnospirales | Lachnospiraceae | Anaerostipes | Anaerostipes hadrus_A | 0.825894 | 0.015217 |
| MGYG000000262_00151 | Firmicutes_A | Clostridia | Lachnospirales | Lachnospiraceae | Anaerobutyricum | Anaerobutyricum hallii | 0.8241 | 0.001951 |
| MGYG000003702_01911 | Firmicutes_A | Clostridia | Lachnospirales | Lachnospiraceae | Eisenbergiella | Eisenbergiella sp900066775 | 0.822367 | 0.013222 |
| MGYG000000193_02113 | Firmicutes_A | Clostridia | Lachnospirales | Lachnospiraceae | KLE1615 | KLE1615 sp900066985 | 0.819789 | 0.000804 |
| MGYG000002670_00676 | Firmicutes_A | Clostridia | Lachnospirales | Lachnospiraceae | Agathobacter | Agathobacter sp900546625 | 0.819507 | 0.004071 |
| MGYG000004733_00968 | Firmicutes_A | Clostridia | Lachnospirales | Lachnospiraceae | Blautia_A | Blautia_A sp900548245 | 0.819262 | 0.003617 |
| MGYG000002298_00165 | Firmicutes_A | Clostridia | Lachnospirales | Lachnospiraceae | Blautia_A | Blautia_A sp000436615 | 0.818807 | 0.001673 |
| MGYG000002561_02267 | Bacteroidota | Bacteroidia | Bacteroidales | Bacteroidaceae | Bacteroides | Bacteroides sp902388495 | 0.818783 | 0.001787 |
| MGYG000002298_02427 | Firmicutes_A | Clostridia | Lachnospirales | Lachnospiraceae | Blautia_A | Blautia_A sp000436615 | 0.818656 | 0.021446 |
| MGYG000002445_00875 | Firmicutes_A | Clostridia | Lachnospirales | Lachnospiraceae | Clostridium_Q | Clostridium_Q sp003024715 | 0.818343 | 0.002926 |
| MGYG000000201_02093 | Firmicutes_A | Clostridia | Lachnospirales | Lachnospiraceae | Blautia_A | Blautia_A sp900066145 | 0.81777 | 0.011789 |
| MGYG000002492_01789 | Firmicutes_A | Clostridia | Lachnospirales | Lachnospiraceae | Agathobacter | Agathobacter rectalis | 0.817137 | 0.015963 |
| MGYG000000774_01752 | Firmicutes_A | Clostridia | Lachnospirales | Lachnospiraceae | Enterocloster | Enterocloster sp900543885 | 0.814203 | 0.001212 |
| MGYG000002438_03137 | Bacteroidota | Bacteroidia | Bacteroidales | Tannerellaceae | Parabacteroides | Parabacteroides distasonis | 0.813805 | 0.000427 |
| MGYG000002560_02568 | Bacteroidota | Bacteroidia | Bacteroidales | Bacteroidaceae | Phocaeicola | Phocaeicola sp902388365 | 0.813247 | 0.010146 |
| MGYG000001346_03375 | Bacteroidota | Bacteroidia | Bacteroidales | Bacteroidaceae | Bacteroides | Bacteroides uniformis | 0.812708 | 0.001066 |
| MGYG000003694_00529 | Firmicutes_A | Clostridia | Lachnospirales | Lachnospiraceae | Agathobacter | Agathobacter faecis | 0.81165 | 0.021248 |
| MGYG000002478_04156 | Bacteroidota | Bacteroidia | Bacteroidales | Bacteroidaceae | Phocaeicola | Phocaeicola dorei | 0.811215 | 0.011539 |
| MGYG000001346_02837 | Bacteroidota | Bacteroidia | Bacteroidales | Bacteroidaceae | Bacteroides | Bacteroides uniformis | 0.810234 | 0.002599 |
| MGYG000002478_04098 | Bacteroidota | Bacteroidia | Bacteroidales | Bacteroidaceae | Phocaeicola | Phocaeicola dorei | 0.810121 | 0.023927 |
| MGYG000000209_01190 | Firmicutes_A | Clostridia | Lachnospirales | Lachnospiraceae | Eubacterium_F | Eubacterium_F sp003491505 | 0.80852 | 0.039886 |
| MGYG000002478_00892 | Bacteroidota | Bacteroidia | Bacteroidales | Bacteroidaceae | Phocaeicola | Phocaeicola dorei | 0.807888 | 0.000415 |
| MGYG000002478_03896 | Bacteroidota | Bacteroidia | Bacteroidales | Bacteroidaceae | Phocaeicola | Phocaeicola dorei | 0.807832 | 0.003741 |
| MGYG000004250_02312 | Firmicutes_A | Clostridia | Lachnospirales | Lachnospiraceae | Acetatifactor | Acetatifactor sp003447295 | 0.807822 | 0.00359 |
| MGYG000004489_00637 | Firmicutes_A | Clostridia | Oscillospirales | Oscillospiraceae | ER4 | ER4 sp900552015 | 0.805588 | 0.017439 |
| MGYG000000200_03516 | Firmicutes_A | Clostridia | Lachnospirales | Lachnospiraceae | Blautia_A | Blautia_A sp003471165 | 0.804916 | 0.003116 |
| MGYG000002478_00983 | Bacteroidota | Bacteroidia | Bacteroidales | Bacteroidaceae | Phocaeicola | Phocaeicola dorei | 0.804727 | 0.00359 |
| MGYG000001302.1_01598 | Bacteroidota | Bacteroidia | Bacteroidales | Rikenellaceae | Alistipes | Alistipes putredinis | 0.804547 | 0.01336 |
| MGYG000004479_03004 | Bacteroidota | Bacteroidia | Bacteroidales | Bacteroidaceae | Phocaeicola | Phocaeicola mediterraneensis | 0.804483 | 0.015152 |
| MGYG000002837_02343 | Firmicutes_A | Clostridia | Lachnospirales | Lachnospiraceae | Blautia_A | Blautia_A sp900542045 | 0.802262 | 0.003256 |
| MGYG000002492_00425 | Firmicutes_A | Clostridia | Lachnospirales | Lachnospiraceae | Agathobacter | Agathobacter rectalis | 0.801574 | 0.021784 |
| MGYG000002492_02667 | Firmicutes_A | Clostridia | Lachnospirales | Lachnospiraceae | Agathobacter | Agathobacter rectalis | 0.798571 | 0.010432 |
| MGYG000002478_03016 | Bacteroidota | Bacteroidia | Bacteroidales | Bacteroidaceae | Phocaeicola | Phocaeicola dorei | 0.796201 | 0.025273 |
| MGYG000002033_02102 | Bacteroidota | Bacteroidia | Bacteroidales | Tannerellaceae | Parabacteroides | Parabacteroides massiliensis | 0.795511 | 0.006152 |
| MGYG000000271_03320 | Firmicutes_A | Clostridia | Lachnospirales | Lachnospiraceae | Roseburia | Roseburia sp900552665 | 0.79451 | 0.040645 |
| MGYG000002478_00984 | Bacteroidota | Bacteroidia | Bacteroidales | Bacteroidaceae | Phocaeicola | Phocaeicola dorei | 0.793846 | 0.00038 |
| MGYG000000031_00369 | Firmicutes_A | Clostridia | Lachnospirales | Lachnospiraceae | Blautia_A | Blautia_A sp900066205 | 0.790032 | 0.015138 |
| MGYG000000216_01951 | Firmicutes_A | Clostridia | Lachnospirales | Lachnospiraceae | Blautia_A | Blautia_A sp003474435 | 0.789627 | 0.002249 |
| MGYG000002478_01108 | Bacteroidota | Bacteroidia | Bacteroidales | Bacteroidaceae | Phocaeicola | Phocaeicola dorei | 0.789589 | 0.001754 |
| MGYG000002560_01465 | Bacteroidota | Bacteroidia | Bacteroidales | Bacteroidaceae | Phocaeicola | Phocaeicola sp902388365 | 0.788822 | 0.001162 |
| MGYG000002478_04392 | Bacteroidota | Bacteroidia | Bacteroidales | Bacteroidaceae | Phocaeicola | Phocaeicola dorei | 0.787577 | 0.002532 |
| MGYG000002478_02442 | Bacteroidota | Bacteroidia | Bacteroidales | Bacteroidaceae | Phocaeicola | Phocaeicola dorei | 0.787308 | 0.006755 |
| MGYG000001302.1_01170 | Bacteroidota | Bacteroidia | Bacteroidales | Rikenellaceae | Alistipes | Alistipes putredinis | 0.786898 | 0.004726 |
| MGYG000000251_01241 | Firmicutes_A | Clostridia | Lachnospirales | Lachnospiraceae | Fusicatenibacter | Fusicatenibacter saccharivorans | 0.784246 | 0.002249 |
| MGYG000002040_00479 | Firmicutes_A | Clostridia | Oscillospirales | Ruminococcaceae | Faecalibacterium | Faecalibacterium sp900758465 | 0.783839 | 0.017971 |
| MGYG000004276_01041 | Firmicutes_A | Clostridia | Oscillospirales | Oscillospiraceae | ER4 | ER4 sp900550165 | 0.781967 | 0.000851 |
| MGYG000000212_00998 | Firmicutes_A | Clostridia | Lachnospirales | Lachnospiraceae | Blautia_A | Blautia_A obeum | 0.780657 | 0.01197 |
| MGYG000000212_02552 | Firmicutes_A | Clostridia | Lachnospirales | Lachnospiraceae | Blautia_A | Blautia_A obeum | 0.779691 | 0.026901 |
| MGYG000002619_02100 | Firmicutes_A | Clostridia | Oscillospirales | Ruminococcaceae | Faecalibacterium | Faecalibacterium prausnitzii_J | 0.777116 | 0.021446 |
| MGYG000000136_01313 | Firmicutes_A | Clostridia | Lachnospirales | Lachnospiraceae | Agathobacter | Agathobacter sp000434275 | 0.775839 | 0.011405 |
| MGYG000000142_01711 | Firmicutes_A | Clostridia | Lachnospirales | Lachnospiraceae | Blautia_A | Blautia_A massiliensis | 0.775043 | 0.010898 |
| MGYG000001189_01215 | Firmicutes_A | Clostridia | Lachnospirales | Lachnospiraceae | Blautia_A |  | 0.774558 | 0.000897 |
| MGYG000000039_00553 | Firmicutes_A | Clostridia | Oscillospirales | Ruminococcaceae | Faecalibacterium | Faecalibacterium prausnitzii_H | 0.772923 | 0.013197 |
| MGYG000000193_00206 | Firmicutes_A | Clostridia | Lachnospirales | Lachnospiraceae | KLE1615 | KLE1615 sp900066985 | 0.770955 | 0.005434 |
| MGYG000002715_01094 | Firmicutes_A | Clostridia | Oscillospirales | Oscillospiraceae | ER4 | ER4 sp900317525 | 0.77086 | 0.003537 |
| MGYG000000054_00456 | Bacteroidota | Bacteroidia | Bacteroidales | Bacteroidaceae | Bacteroides | Bacteroides acidifaciens | 0.770726 | 0.026565 |
| MGYG000002528_00841 | Firmicutes_A | Clostridia | Lachnospirales | Lachnospiraceae | Anaerostipes | Anaerostipes hadrus | 0.770425 | 0.000881 |
| MGYG000000022_01320 | Firmicutes_A | Clostridia | Oscillospirales | Ruminococcaceae | Faecalibacterium | Faecalibacterium prausnitzii_C | 0.76991 | 0.029282 |
| MGYG000001338_03568 | Firmicutes_A | Clostridia | Lachnospirales | Lachnospiraceae | Blautia_A | Blautia_A wexlerae_A | 0.769835 | 0.016972 |
| MGYG000002438_00951 | Bacteroidota | Bacteroidia | Bacteroidales | Tannerellaceae | Parabacteroides | Parabacteroides distasonis | 0.769779 | 0.022148 |
| MGYG000003702_01249 | Firmicutes_A | Clostridia | Lachnospirales | Lachnospiraceae | Eisenbergiella | Eisenbergiella sp900066775 | 0.768293 | 0.017315 |
| MGYG000003937_00260 | Firmicutes_A | Clostridia | Oscillospirales | Ruminococcaceae | Gemmiger | Gemmiger qucibialis | 0.767369 | 0.001673 |
| MGYG000000028_00361 | Firmicutes_A | Clostridia | Lachnospirales | Lachnospiraceae | Anaerostipes | Anaerostipes hadrus_A | 0.765713 | 0.012408 |
| MGYG000000054_04096 | Bacteroidota | Bacteroidia | Bacteroidales | Bacteroidaceae | Bacteroides | Bacteroides acidifaciens | 0.765368 | 0.022862 |
| MGYG000000201_02278 | Firmicutes_A | Clostridia | Lachnospirales | Lachnospiraceae | Blautia_A | Blautia_A sp900066145 | 0.764906 | 0.000195 |
| MGYG000002528_01695 | Firmicutes_A | Clostridia | Lachnospirales | Lachnospiraceae | Anaerostipes | Anaerostipes hadrus | 0.764538 | 0.010436 |
| MGYG000002298_03813 | Firmicutes_A | Clostridia | Lachnospirales | Lachnospiraceae | Blautia_A | Blautia_A sp000436615 | 0.763579 | 0.005626 |
| MGYG000000262_01443 | Firmicutes_A | Clostridia | Lachnospirales | Lachnospiraceae | Anaerobutyricum | Anaerobutyricum hallii | 0.763091 | 0.010387 |
| MGYG000000193_02115 | Firmicutes_A | Clostridia | Lachnospirales | Lachnospiraceae | KLE1615 | KLE1615 sp900066985 | 0.761688 | 0.037406 |
| MGYG000000002_00716 | Firmicutes_A | Clostridia | Lachnospirales | Lachnospiraceae | Blautia_A | Blautia_A faecis | 0.761659 | 0.037083 |
| MGYG000000193_02096 | Firmicutes_A | Clostridia | Lachnospirales | Lachnospiraceae | KLE1615 | KLE1615 sp900066985 | 0.761642 | 0.011253 |
| MGYG000002040_00240 | Firmicutes_A | Clostridia | Oscillospirales | Ruminococcaceae | Faecalibacterium | Faecalibacterium sp900758465 | 0.75936 | 0.021399 |
| MGYG000000193_01110 | Firmicutes_A | Clostridia | Lachnospirales | Lachnospiraceae | KLE1615 | KLE1615 sp900066985 | 0.758493 | 0.002391 |
| MGYG000002478_00038 | Bacteroidota | Bacteroidia | Bacteroidales | Bacteroidaceae | Phocaeicola | Phocaeicola dorei | 0.758366 | 0.026486 |
| MGYG000000209_02187 | Firmicutes_A | Clostridia | Lachnospirales | Lachnospiraceae | Eubacterium_F | Eubacterium_F sp003491505 | 0.754591 | 0.039886 |
| MGYG000000078_01646 | Firmicutes_A | Clostridia | Lachnospirales | Lachnospiraceae | Lachnospira | Lachnospira rogosae_A | 0.754251 | 0.008921 |
| MGYG000000193_00231 | Firmicutes_A | Clostridia | Lachnospirales | Lachnospiraceae | KLE1615 | KLE1615 sp900066985 | 0.754082 | 0.008171 |
| MGYG000002517_00773 | Firmicutes_A | Clostridia | Lachnospirales | Lachnospiraceae | Roseburia | Roseburia hominis | 0.752237 | 0.004559 |
| MGYG000002300_02470 | Bacteroidota | Bacteroidia | Bacteroidales | Bacteroidaceae | Bacteroides | Bacteroides cutis | 0.75097 | 0.000897 |
| MGYG000002478_01528 | Bacteroidota | Bacteroidia | Bacteroidales | Bacteroidaceae | Phocaeicola | Phocaeicola dorei | 0.744469 | 0.013288 |
| MGYG000001346_03104 | Bacteroidota | Bacteroidia | Bacteroidales | Bacteroidaceae | Bacteroides | Bacteroides uniformis | 0.744169 | 0.000703 |
| MGYG000002545_02315 | Firmicutes_A | Clostridia | Oscillospirales | Ruminococcaceae | Faecalibacterium | Faecalibacterium prausnitzii_G | 0.743065 | 0.000856 |
| MGYG000000236_00268 | Bacteroidota | Bacteroidia | Bacteroidales | Bacteroidaceae | Bacteroides | Bacteroides fragilis_A | 0.740986 | 0.011253 |
| MGYG000000262_01289 | Firmicutes_A | Clostridia | Lachnospirales | Lachnospiraceae | Anaerobutyricum | Anaerobutyricum hallii | 0.740722 | 0.01654 |
| MGYG000003695_02209 | Firmicutes_A | Clostridia | Lachnospirales | Lachnospiraceae | Ruminococcus_A | Ruminococcus_A sp003011855 | 0.739141 | 0.003386 |
| MGYG000002619_02257 | Firmicutes_A | Clostridia | Oscillospirales | Ruminococcaceae | Faecalibacterium | Faecalibacterium prausnitzii_J | 0.737343 | 0.014508 |
| MGYG000000028_01835 | Firmicutes_A | Clostridia | Lachnospirales | Lachnospiraceae | Anaerostipes | Anaerostipes hadrus_A | 0.736718 | 0.040115 |
| MGYG000002670_01982 | Firmicutes_A | Clostridia | Lachnospirales | Lachnospiraceae | Agathobacter | Agathobacter sp900546625 | 0.734647 | 0.016152 |
| MGYG000000031_01448 | Firmicutes_A | Clostridia | Lachnospirales | Lachnospiraceae | Blautia_A | Blautia_A sp900066205 | 0.734113 | 0.001422 |
| MGYG000002478_00687 | Bacteroidota | Bacteroidia | Bacteroidales | Bacteroidaceae | Phocaeicola | Phocaeicola dorei | 0.733336 | 0.039752 |
| MGYG000002492_02660 | Firmicutes_A | Clostridia | Lachnospirales | Lachnospiraceae | Agathobacter | Agathobacter rectalis | 0.732434 | 0.02042 |
| MGYG000000212_03047 | Firmicutes_A | Clostridia | Lachnospirales | Lachnospiraceae | Blautia_A | Blautia_A obeum | 0.73217 | 0.002632 |
| MGYG000002478_00792 | Bacteroidota | Bacteroidia | Bacteroidales | Bacteroidaceae | Phocaeicola | Phocaeicola dorei | 0.732163 | 0.004866 |
| MGYG000004797_01301 | Bacteroidota | Bacteroidia | Bacteroidales | Bacteroidaceae | Phocaeicola | Phocaeicola sartorii | 0.731068 | 0.00668 |
| MGYG000001338_00486 | Firmicutes_A | Clostridia | Lachnospirales | Lachnospiraceae | Blautia_A | Blautia_A wexlerae_A | 0.729511 | 0.009984 |
| MGYG000000002_00525 | Firmicutes_A | Clostridia | Lachnospirales | Lachnospiraceae | Blautia_A | Blautia_A faecis | 0.728879 | 0.029028 |
| MGYG000000039_01781 | Firmicutes_A | Clostridia | Oscillospirales | Ruminococcaceae | Faecalibacterium | Faecalibacterium prausnitzii_H | 0.727245 | 0.000851 |
| MGYG000001338_02720 | Firmicutes_A | Clostridia | Lachnospirales | Lachnospiraceae | Blautia_A | Blautia_A wexlerae_A | 0.726799 | 0.000851 |
| MGYG000000258_00235 | Firmicutes_A | Clostridia | Oscillospirales | Acutalibacteraceae | Ruminococcus_E | Ruminococcus_E bromii_B | 0.72584 | 0.006181 |
| MGYG000000200_01831 | Firmicutes_A | Clostridia | Lachnospirales | Lachnospiraceae | Blautia_A | Blautia_A sp003471165 | 0.725641 | 0.00194 |
| MGYG000002438_02321 | Bacteroidota | Bacteroidia | Bacteroidales | Tannerellaceae | Parabacteroides | Parabacteroides distasonis | 0.725507 | 0.040025 |
| MGYG000002040_00906 | Firmicutes_A | Clostridia | Oscillospirales | Ruminococcaceae | Faecalibacterium | Faecalibacterium sp900758465 | 0.72295 | 0.004114 |
| MGYG000000140_00430 | Firmicutes_A | Clostridia | Lachnospirales | Lachnospiraceae | UMGS1375 | UMGS1375 sp900066615 | 0.719628 | 0.000244 |
| MGYG000001302.1_00235 | Bacteroidota | Bacteroidia | Bacteroidales | Rikenellaceae | Alistipes | Alistipes putredinis | 0.719518 | 0.04501 |
| MGYG000000002_01924 | Firmicutes_A | Clostridia | Lachnospirales | Lachnospiraceae | Blautia_A | Blautia_A faecis | 0.717876 | 0.020347 |
| MGYG000002040_01511 | Firmicutes_A | Clostridia | Oscillospirales | Ruminococcaceae | Faecalibacterium | Faecalibacterium sp900758465 | 0.716165 | 0.00072 |
| MGYG000000193_01000 | Firmicutes_A | Clostridia | Lachnospirales | Lachnospiraceae | KLE1615 | KLE1615 sp900066985 | 0.715303 | 0.03405 |
| MGYG000004185_01705 | Bacteroidota | Bacteroidia | Bacteroidales | Bacteroidaceae | Bacteroides | Bacteroides sp900553815 | 0.715142 | 0.001648 |
| MGYG000001255_01113 | Firmicutes_A | Clostridia | Oscillospirales | Ruminococcaceae | Faecalibacterium | Faecalibacterium prausnitzii_F | 0.714447 | 0.024866 |
| MGYG000002478_00707 | Bacteroidota | Bacteroidia | Bacteroidales | Bacteroidaceae | Phocaeicola | Phocaeicola dorei | 0.713585 | 0.014941 |
| MGYG000003695_01266 | Firmicutes_A | Clostridia | Lachnospirales | Lachnospiraceae | Ruminococcus_A | Ruminococcus_A sp003011855 | 0.712334 | 0.006906 |
| MGYG000002492_02065 | Firmicutes_A | Clostridia | Lachnospirales | Lachnospiraceae | Agathobacter | Agathobacter rectalis | 0.711047 | 0.004711 |
| MGYG000000039_01704 | Firmicutes_A | Clostridia | Oscillospirales | Ruminococcaceae | Faecalibacterium | Faecalibacterium prausnitzii_H | 0.711006 | 0.001526 |
| MGYG000000217_01571 | Firmicutes_A | Clostridia | Lachnospirales | Lachnospiraceae | Acetatifactor | Acetatifactor sp900066565 | 0.710733 | 0.003061 |
| MGYG000002545_01914 | Firmicutes_A | Clostridia | Oscillospirales | Ruminococcaceae | Faecalibacterium | Faecalibacterium prausnitzii_G | 0.710666 | 0.012226 |
| MGYG000000028_01595 | Firmicutes_A | Clostridia | Lachnospirales | Lachnospiraceae | Anaerostipes | Anaerostipes hadrus_A | 0.706625 | 0.020057 |
| MGYG000002492_01112 | Firmicutes_A | Clostridia | Lachnospirales | Lachnospiraceae | Agathobacter | Agathobacter rectalis | 0.705304 | 0.005212 |
| MGYG000000245_02146 | Firmicutes_A | Clostridia | Lachnospirales | Lachnospiraceae | Roseburia | Roseburia sp003470905 | 0.703695 | 0.021989 |
| MGYG000000258_00287 | Firmicutes_A | Clostridia | Oscillospirales | Acutalibacteraceae | Ruminococcus_E | Ruminococcus_E bromii_B | 0.702704 | 0.032033 |
| MGYG000002272_00314 | Firmicutes_A | Clostridia | Oscillospirales | Ruminococcaceae | Faecalibacterium | Faecalibacterium prausnitzii_D | 0.702558 | 0.003578 |
| MGYG000000078_02434 | Firmicutes_A | Clostridia | Lachnospirales | Lachnospiraceae | Lachnospira | Lachnospira rogosae_A | 0.702244 | 0.001952 |
| MGYG000002478_00894 | Bacteroidota | Bacteroidia | Bacteroidales | Bacteroidaceae | Phocaeicola | Phocaeicola dorei | 0.701939 | 0.007315 |
| MGYG000000251_00650 | Firmicutes_A | Clostridia | Lachnospirales | Lachnospiraceae | Fusicatenibacter | Fusicatenibacter saccharivorans | 0.701843 | 0.048438 |
| MGYG000002478_01472 | Bacteroidota | Bacteroidia | Bacteroidales | Bacteroidaceae | Phocaeicola | Phocaeicola dorei | 0.701137 | 0.020956 |
| MGYG000000022_02872 | Firmicutes_A | Clostridia | Oscillospirales | Ruminococcaceae | Faecalibacterium | Faecalibacterium prausnitzii_C | 0.700469 | 0.001051 |
| MGYG000002478_01954 | Bacteroidota | Bacteroidia | Bacteroidales | Bacteroidaceae | Phocaeicola | Phocaeicola dorei | 0.699978 | 0.009464 |
| MGYG000000251_01829 | Firmicutes_A | Clostridia | Lachnospirales | Lachnospiraceae | Fusicatenibacter | Fusicatenibacter saccharivorans | 0.69992 | 0.000204 |
| MGYG000001306_00798 | Bacteroidota | Bacteroidia | Bacteroidales | Bacteroidaceae | Phocaeicola | Phocaeicola coprocola | 0.697555 | 0.021009 |
| MGYG000001255_00450 | Firmicutes_A | Clostridia | Oscillospirales | Ruminococcaceae | Faecalibacterium | Faecalibacterium prausnitzii_F | 0.696396 | 0.002486 |
| MGYG000000212_02591 | Firmicutes_A | Clostridia | Lachnospirales | Lachnospiraceae | Blautia_A | Blautia_A obeum | 0.695465 | 0.038746 |
| MGYG000000201_01176 | Firmicutes_A | Clostridia | Lachnospirales | Lachnospiraceae | Blautia_A | Blautia_A sp900066145 | 0.695433 | 0.001653 |
| MGYG000000251_01869 | Firmicutes_A | Clostridia | Lachnospirales | Lachnospiraceae | Fusicatenibacter | Fusicatenibacter saccharivorans | 0.693765 | 0.008523 |
| MGYG000002478_01912 | Bacteroidota | Bacteroidia | Bacteroidales | Bacteroidaceae | Phocaeicola | Phocaeicola dorei | 0.693593 | 0.001883 |
| MGYG000000251_00651 | Firmicutes_A | Clostridia | Lachnospirales | Lachnospiraceae | Fusicatenibacter | Fusicatenibacter saccharivorans | 0.69302 | 0.018853 |
| MGYG000001346_03049 | Bacteroidota | Bacteroidia | Bacteroidales | Bacteroidaceae | Bacteroides | Bacteroides uniformis | 0.692829 | 0.02482 |
| MGYG000000262_01352 | Firmicutes_A | Clostridia | Lachnospirales | Lachnospiraceae | Anaerobutyricum | Anaerobutyricum hallii | 0.690525 | 0.037469 |
| MGYG000003702_02510 | Firmicutes_A | Clostridia | Lachnospirales | Lachnospiraceae | Eisenbergiella | Eisenbergiella sp900066775 | 0.689763 | 0.003528 |
| MGYG000002298_01760 | Firmicutes_A | Clostridia | Lachnospirales | Lachnospiraceae | Blautia_A | Blautia_A sp000436615 | 0.689236 | 0.01424 |
| MGYG000001346_01526 | Bacteroidota | Bacteroidia | Bacteroidales | Bacteroidaceae | Bacteroides | Bacteroides uniformis | 0.688901 | 0.022676 |
| MGYG000000209_01925 | Firmicutes_A | Clostridia | Lachnospirales | Lachnospiraceae | Eubacterium_F | Eubacterium_F sp003491505 | 0.688835 | 0.004289 |
| MGYG000001346_01378 | Bacteroidota | Bacteroidia | Bacteroidales | Bacteroidaceae | Bacteroides | Bacteroides uniformis | 0.688587 | 0.024322 |
| MGYG000000251_01815 | Firmicutes_A | Clostridia | Lachnospirales | Lachnospiraceae | Fusicatenibacter | Fusicatenibacter saccharivorans | 0.687769 | 0.035331 |
| MGYG000000060_01662 | Firmicutes_A | Clostridia | Lachnospirales | Lachnospiraceae | Lachnospira | Lachnospira eligens_A | 0.687675 | 0.033498 |
| MGYG000002492_01612 | Firmicutes_A | Clostridia | Lachnospirales | Lachnospiraceae | Agathobacter | Agathobacter rectalis | 0.686691 | 0.047149 |
| MGYG000000002_03096 | Firmicutes_A | Clostridia | Lachnospirales | Lachnospiraceae | Blautia_A | Blautia_A faecis | 0.686574 | 0.02168 |
| MGYG000000078_01005 | Firmicutes_A | Clostridia | Lachnospirales | Lachnospiraceae | Lachnospira | Lachnospira rogosae_A | 0.686454 | 0.031183 |
| MGYG000002298_00428 | Firmicutes_A | Clostridia | Lachnospirales | Lachnospiraceae | Blautia_A | Blautia_A sp000436615 | 0.686091 | 0.001922 |
| MGYG000000251_00075 | Firmicutes_A | Clostridia | Lachnospirales | Lachnospiraceae | Fusicatenibacter | Fusicatenibacter saccharivorans | 0.685892 | 0.035462 |
| MGYG000000251_01807 | Firmicutes_A | Clostridia | Lachnospirales | Lachnospiraceae | Fusicatenibacter | Fusicatenibacter saccharivorans | 0.684757 | 0.001666 |
| MGYG000002545_02429 | Firmicutes_A | Clostridia | Oscillospirales | Ruminococcaceae | Faecalibacterium | Faecalibacterium prausnitzii_G | 0.683435 | 0.00413 |
| MGYG000000217_01273 | Firmicutes_A | Clostridia | Lachnospirales | Lachnospiraceae | Acetatifactor | Acetatifactor sp900066565 | 0.683104 | 0.002926 |
| MGYG000000142_02317 | Firmicutes_A | Clostridia | Lachnospirales | Lachnospiraceae | Blautia_A | Blautia_A massiliensis | 0.683088 | 0.016152 |
| MGYG000000262_02775 | Firmicutes_A | Clostridia | Lachnospirales | Lachnospiraceae | Anaerobutyricum | Anaerobutyricum hallii | 0.682963 | 0.035462 |
| MGYG000000271_01063 | Firmicutes_A | Clostridia | Lachnospirales | Lachnospiraceae | Roseburia | Roseburia sp900552665 | 0.682544 | 0.002632 |
| MGYG000002545_01702 | Firmicutes_A | Clostridia | Oscillospirales | Ruminococcaceae | Faecalibacterium | Faecalibacterium prausnitzii_G | 0.682 | 0.02183 |
| MGYG000000209_01916 | Firmicutes_A | Clostridia | Lachnospirales | Lachnospiraceae | Eubacterium_F | Eubacterium_F sp003491505 | 0.681875 | 0.013221 |
| MGYG000000031_00567 | Firmicutes_A | Clostridia | Lachnospirales | Lachnospiraceae | Blautia_A | Blautia_A sp900066205 | 0.681521 | 0.012562 |
| MGYG000002492_02536 | Firmicutes_A | Clostridia | Lachnospirales | Lachnospiraceae | Agathobacter | Agathobacter rectalis | 0.678392 | 0.013476 |
| MGYG000002478_04434 | Bacteroidota | Bacteroidia | Bacteroidales | Bacteroidaceae | Phocaeicola | Phocaeicola dorei | 0.677791 | 0.001215 |
| MGYG000000002_02254 | Firmicutes_A | Clostridia | Lachnospirales | Lachnospiraceae | Blautia_A | Blautia_A faecis | 0.677781 | 0.003056 |
| MGYG000002530_00143 | Firmicutes_A | Clostridia | Oscillospirales | Ruminococcaceae | Ruminiclostridium_E | Ruminiclostridium_E siraeum | 0.675737 | 0.021248 |
| MGYG000001338_00615 | Firmicutes_A | Clostridia | Lachnospirales | Lachnospiraceae | Blautia_A | Blautia_A wexlerae_A | 0.675535 | 0.019499 |
| MGYG000001338_03224 | Firmicutes_A | Clostridia | Lachnospirales | Lachnospiraceae | Blautia_A | Blautia_A wexlerae_A | 0.67542 | 0.000354 |
| MGYG000002478_01454 | Bacteroidota | Bacteroidia | Bacteroidales | Bacteroidaceae | Phocaeicola | Phocaeicola dorei | 0.674167 | 0.001107 |
| MGYG000002445_00092 | Firmicutes_A | Clostridia | Lachnospirales | Lachnospiraceae | Clostridium_Q | Clostridium_Q sp003024715 | 0.672272 | 0.001913 |
| MGYG000002478_04560 | Bacteroidota | Bacteroidia | Bacteroidales | Bacteroidaceae | Phocaeicola | Phocaeicola dorei | 0.672083 | 0.015399 |
| MGYG000000193_02101 | Firmicutes_A | Clostridia | Lachnospirales | Lachnospiraceae | KLE1615 | KLE1615 sp900066985 | 0.672065 | 0.022676 |
| MGYG000001346_03273 | Bacteroidota | Bacteroidia | Bacteroidales | Bacteroidaceae | Bacteroides | Bacteroides uniformis | 0.671515 | 0.005379 |
| MGYG000001346_00032 | Bacteroidota | Bacteroidia | Bacteroidales | Bacteroidaceae | Bacteroides | Bacteroides uniformis | 0.669817 | 0.010436 |
| MGYG000000060_01819 | Firmicutes_A | Clostridia | Lachnospirales | Lachnospiraceae | Lachnospira | Lachnospira eligens_A | 0.668716 | 0.042364 |
| MGYG000002274_00511 | Firmicutes_A | Clostridia | Oscillospirales | Ruminococcaceae | Faecalibacterium | Faecalibacterium prausnitzii_I | 0.664687 | 0.006143 |
| MGYG000002478_01883 | Bacteroidota | Bacteroidia | Bacteroidales | Bacteroidaceae | Phocaeicola | Phocaeicola dorei | 0.664418 | 0.006597 |
| MGYG000004276_00122 | Firmicutes_A | Clostridia | Oscillospirales | Oscillospiraceae | ER4 | ER4 sp900550165 | 0.662376 | 0.014971 |
| MGYG000004296_01631 | Firmicutes_A | Clostridia | Lachnospirales | Lachnospiraceae | Ruminococcus_A | Ruminococcus_A faecicola | 0.661588 | 0.005564 |
| MGYG000000054_04463 | Bacteroidota | Bacteroidia | Bacteroidales | Bacteroidaceae | Bacteroides | Bacteroides acidifaciens | 0.659014 | 0.006365 |
| MGYG000002641_00053 | Firmicutes_A | Clostridia | Oscillospirales | Ruminococcaceae | Faecalibacterium | Faecalibacterium sp003449675 | 0.658759 | 0.010611 |
| MGYG000000142_02251 | Firmicutes_A | Clostridia | Lachnospirales | Lachnospiraceae | Blautia_A | Blautia_A massiliensis | 0.658093 | 0.002704 |
| MGYG000002528_02669 | Firmicutes_A | Clostridia | Lachnospirales | Lachnospiraceae | Anaerostipes | Anaerostipes hadrus | 0.657872 | 0.021446 |
| MGYG000000404_00624 | Firmicutes_A | Clostridia | Lachnospirales | Lachnospiraceae | UBA11774 | UBA11774 sp003507655 | 0.65783 | 0.001754 |
| MGYG000000262_00161 | Firmicutes_A | Clostridia | Lachnospirales | Lachnospiraceae | Anaerobutyricum | Anaerobutyricum hallii | 0.657318 | 0.038352 |
| MGYG000002492_01248 | Firmicutes_A | Clostridia | Lachnospirales | Lachnospiraceae | Agathobacter | Agathobacter rectalis | 0.657222 | 0.003537 |
| MGYG000002670_01951 | Firmicutes_A | Clostridia | Lachnospirales | Lachnospiraceae | Agathobacter | Agathobacter sp900546625 | 0.657091 | 0.021114 |
| MGYG000000146_00570 | Firmicutes_A | Clostridia | Lachnospirales | Lachnospiraceae | Dorea | Dorea formicigenerans | 0.6567 | 0.003116 |
| MGYG000000245_02653 | Firmicutes_A | Clostridia | Lachnospirales | Lachnospiraceae | Roseburia | Roseburia sp003470905 | 0.656132 | 0.02129 |
| MGYG000000039_02397 | Firmicutes_A | Clostridia | Oscillospirales | Ruminococcaceae | Faecalibacterium | Faecalibacterium prausnitzii_H | 0.655763 | 0.02272 |
| MGYG000002478_02463 | Bacteroidota | Bacteroidia | Bacteroidales | Bacteroidaceae | Phocaeicola | Phocaeicola dorei | 0.655716 | 0.048438 |
| MGYG000001456.1_00939 | Firmicutes_A | Clostridia | Lachnospirales | Lachnospiraceae | Eubacterium_I | Eubacterium_I ramulus | 0.654577 | 0.042059 |
| MGYG000002835_00316 | Firmicutes_A | Clostridia | Lachnospirales | Lachnospiraceae | Fusicatenibacter | Fusicatenibacter sp900543115 | 0.653254 | 0.005952 |
| MGYG000002492_01453 | Firmicutes_A | Clostridia | Lachnospirales | Lachnospiraceae | Agathobacter | Agathobacter rectalis | 0.653108 | 0.005138 |
| MGYG000002478_01278 | Bacteroidota | Bacteroidia | Bacteroidales | Bacteroidaceae | Phocaeicola | Phocaeicola dorei | 0.651787 | 0.034008 |
| MGYG000001689_01982 | Firmicutes_A | Clostridia | Lachnospirales | Lachnospiraceae | Blautia | Blautia coccoides | 0.651463 | 0.001217 |
| MGYG000000039_01701 | Firmicutes_A | Clostridia | Oscillospirales | Ruminococcaceae | Faecalibacterium | Faecalibacterium prausnitzii_H | 0.650382 | 0.001051 |
| MGYG000000990_02212 | Firmicutes_A | Clostridia | Lachnospirales | Lachnospiraceae | Blautia | Blautia sp900539145 | 0.648377 | 0.001913 |
| MGYG000002478_00270 | Bacteroidota | Bacteroidia | Bacteroidales | Bacteroidaceae | Phocaeicola | Phocaeicola dorei | 0.647472 | 0.01906 |
| MGYG000000201_02121 | Firmicutes_A | Clostridia | Lachnospirales | Lachnospiraceae | Blautia_A | Blautia_A sp900066145 | 0.647184 | 0.000415 |
| MGYG000000263_02521 | Firmicutes_A | Clostridia | Lachnospirales | Lachnospiraceae | Blautia_A | Blautia_A sp900066335 | 0.647146 | 0.032599 |
| MGYG000002478_02451 | Bacteroidota | Bacteroidia | Bacteroidales | Bacteroidaceae | Phocaeicola | Phocaeicola dorei | 0.646587 | 0.003901 |
| MGYG000001338_01579 | Firmicutes_A | Clostridia | Lachnospirales | Lachnospiraceae | Blautia_A | Blautia_A wexlerae_A | 0.64644 | 0.005087 |
| MGYG000002438_00750 | Bacteroidota | Bacteroidia | Bacteroidales | Tannerellaceae | Parabacteroides | Parabacteroides distasonis | 0.646231 | 0.001787 |
| MGYG000002478_03963 | Bacteroidota | Bacteroidia | Bacteroidales | Bacteroidaceae | Phocaeicola | Phocaeicola dorei | 0.643943 | 0.003617 |
| MGYG000002478_01959 | Bacteroidota | Bacteroidia | Bacteroidales | Bacteroidaceae | Phocaeicola | Phocaeicola dorei | 0.643468 | 0.005003 |
| MGYG000003695_01379 | Firmicutes_A | Clostridia | Lachnospirales | Lachnospiraceae | Ruminococcus_A | Ruminococcus_A sp003011855 | 0.643202 | 0.017971 |
| MGYG000003352_00184 | Proteobacteria | Gammaproteobacteria | Burkholderiales | Burkholderiaceae | Parasutterella | Parasutterella sp900766055 | 0.641945 | 0.01008 |
| MGYG000000140_02843 | Firmicutes_A | Clostridia | Lachnospirales | Lachnospiraceae | UMGS1375 | UMGS1375 sp900066615 | 0.641692 | 0.049542 |
| MGYG000000251_01718 | Firmicutes_A | Clostridia | Lachnospirales | Lachnospiraceae | Fusicatenibacter | Fusicatenibacter saccharivorans | 0.640804 | 0.012394 |
| MGYG000002478_04001 | Bacteroidota | Bacteroidia | Bacteroidales | Bacteroidaceae | Phocaeicola | Phocaeicola dorei | 0.640686 | 0.003056 |
| MGYG000000028_00551 | Firmicutes_A | Clostridia | Lachnospirales | Lachnospiraceae | Anaerostipes | Anaerostipes hadrus_A | 0.639919 | 0.004381 |
| MGYG000002271_02490 | Firmicutes_A | Clostridia | Lachnospirales | Lachnospiraceae | GCA-900066135 | GCA-900066135 sp900066135 | 0.639356 | 0.009638 |
| MGYG000001338_01448 | Firmicutes_A | Clostridia | Lachnospirales | Lachnospiraceae | Blautia_A | Blautia_A wexlerae_A | 0.638469 | 0.015525 |
| MGYG000000036_00610 | Firmicutes_A | Clostridia | Oscillospirales | Acutalibacteraceae | Pseudoruminococcus | Pseudoruminococcus massiliensis | 0.638068 | 0.013179 |
| MGYG000002492_01019 | Firmicutes_A | Clostridia | Lachnospirales | Lachnospiraceae | Agathobacter | Agathobacter rectalis | 0.636462 | 0.022938 |
| MGYG000002272_01016 | Firmicutes_A | Clostridia | Oscillospirales | Ruminococcaceae | Faecalibacterium | Faecalibacterium prausnitzii_D | 0.634846 | 0.0128 |
| MGYG000000195_01983 | Firmicutes_A | Clostridia | Oscillospirales | Ruminococcaceae | Faecalibacterium | Faecalibacterium prausnitzii_E | 0.634444 | 0.01042 |
| MGYG000000774_01754 | Firmicutes_A | Clostridia | Lachnospirales | Lachnospiraceae | Enterocloster | Enterocloster sp900543885 | 0.63425 | 0.035883 |
| MGYG000001346_00804 | Bacteroidota | Bacteroidia | Bacteroidales | Bacteroidaceae | Bacteroides | Bacteroides uniformis | 0.633541 | 0.009252 |
| MGYG000000022_01644 | Firmicutes_A | Clostridia | Oscillospirales | Ruminococcaceae | Faecalibacterium | Faecalibacterium prausnitzii_C | 0.633513 | 0.000851 |
| MGYG000002478_00910 | Bacteroidota | Bacteroidia | Bacteroidales | Bacteroidaceae | Phocaeicola | Phocaeicola dorei | 0.633011 | 0.021655 |
| MGYG000003702_02335 | Firmicutes_A | Clostridia | Lachnospirales | Lachnospiraceae | Eisenbergiella | Eisenbergiella sp900066775 | 0.632198 | 0.011094 |
| MGYG000000002_03535 | Firmicutes_A | Clostridia | Lachnospirales | Lachnospiraceae | Blautia_A | Blautia_A faecis | 0.631003 | 0.008171 |
| MGYG000001189_00145 | Firmicutes_A | Clostridia | Lachnospirales | Lachnospiraceae | Blautia_A |  | 0.629179 | 0.049632 |
| MGYG000000002_00140 | Firmicutes_A | Clostridia | Lachnospirales | Lachnospiraceae | Blautia_A | Blautia_A faecis | 0.628376 | 0.006906 |
| MGYG000000193_01499 | Firmicutes_A | Clostridia | Lachnospirales | Lachnospiraceae | KLE1615 | KLE1615 sp900066985 | 0.626498 | 0.000331 |
| MGYG000000078_02346 | Firmicutes_A | Clostridia | Lachnospirales | Lachnospiraceae | Lachnospira | Lachnospira rogosae_A | 0.626269 | 0.00224 |
| MGYG000000258_00284 | Firmicutes_A | Clostridia | Oscillospirales | Acutalibacteraceae | Ruminococcus_E | Ruminococcus_E bromii_B | 0.625899 | 0.037596 |
| MGYG000000039_01129 | Firmicutes_A | Clostridia | Oscillospirales | Ruminococcaceae | Faecalibacterium | Faecalibacterium prausnitzii_H | 0.624517 | 0.022938 |
| MGYG000002492_02768 | Firmicutes_A | Clostridia | Lachnospirales | Lachnospiraceae | Agathobacter | Agathobacter rectalis | 0.62352 | 0.010432 |
| MGYG000002545_00279 | Firmicutes_A | Clostridia | Oscillospirales | Ruminococcaceae | Faecalibacterium | Faecalibacterium prausnitzii_G | 0.622206 | 0.00359 |
| MGYG000004797_01443 | Bacteroidota | Bacteroidia | Bacteroidales | Bacteroidaceae | Phocaeicola | Phocaeicola sartorii | 0.621393 | 0.012428 |
| MGYG000002040_01480 | Firmicutes_A | Clostridia | Oscillospirales | Ruminococcaceae | Faecalibacterium | Faecalibacterium sp900758465 | 0.62102 | 0.001787 |
| MGYG000000002_02249 | Firmicutes_A | Clostridia | Lachnospirales | Lachnospiraceae | Blautia_A | Blautia_A faecis | 0.620138 | 0.034016 |
| MGYG000001346_03587 | Bacteroidota | Bacteroidia | Bacteroidales | Bacteroidaceae | Bacteroides | Bacteroides uniformis | 0.619777 | 0.008071 |
| MGYG000001346_00156 | Bacteroidota | Bacteroidia | Bacteroidales | Bacteroidaceae | Bacteroides | Bacteroides uniformis | 0.619524 | 0.030942 |
| MGYG000000251_01820 | Firmicutes_A | Clostridia | Lachnospirales | Lachnospiraceae | Fusicatenibacter | Fusicatenibacter saccharivorans | 0.618303 | 0.032199 |
| MGYG000000213_01795 | Firmicutes_A | Clostridia | Lachnospirales | Lachnospiraceae | Blautia_A | Blautia_A sp003477525 | 0.617507 | 0.043104 |
| MGYG000000251_00723 | Firmicutes_A | Clostridia | Lachnospirales | Lachnospiraceae | Fusicatenibacter | Fusicatenibacter saccharivorans | 0.616965 | 0.003551 |
| MGYG000003695_00856 | Firmicutes_A | Clostridia | Lachnospirales | Lachnospiraceae | Ruminococcus_A | Ruminococcus_A sp003011855 | 0.616959 | 0.035462 |
| MGYG000000251_02008 | Firmicutes_A | Clostridia | Lachnospirales | Lachnospiraceae | Fusicatenibacter | Fusicatenibacter saccharivorans | 0.616464 | 0.024045 |
| MGYG000000142_00171 | Firmicutes_A | Clostridia | Lachnospirales | Lachnospiraceae | Blautia_A | Blautia_A massiliensis | 0.615922 | 0.038935 |
| MGYG000003427_01335 | Firmicutes_A | Clostridia | Lachnospirales | Lachnospiraceae | HGM12587 | HGM12587 sp900766915 | 0.615224 | 0.04501 |
| MGYG000000140_02984 | Firmicutes_A | Clostridia | Lachnospirales | Lachnospiraceae | UMGS1375 | UMGS1375 sp900066615 | 0.614935 | 0.018483 |
| MGYG000000262_01262 | Firmicutes_A | Clostridia | Lachnospirales | Lachnospiraceae | Anaerobutyricum | Anaerobutyricum hallii | 0.614919 | 0.013888 |
| MGYG000003695_01343 | Firmicutes_A | Clostridia | Lachnospirales | Lachnospiraceae | Ruminococcus_A | Ruminococcus_A sp003011855 | 0.61467 | 0.004984 |
| MGYG000002545_01433 | Firmicutes_A | Clostridia | Oscillospirales | Ruminococcaceae | Faecalibacterium | Faecalibacterium prausnitzii_G | 0.613657 | 0.044215 |
| MGYG000002040_01212 | Firmicutes_A | Clostridia | Oscillospirales | Ruminococcaceae | Faecalibacterium | Faecalibacterium sp900758465 | 0.612522 | 0.022676 |
| MGYG000000193_03301 | Firmicutes_A | Clostridia | Lachnospirales | Lachnospiraceae | KLE1615 | KLE1615 sp900066985 | 0.612035 | 0.005764 |
| MGYG000002670_00394 | Firmicutes_A | Clostridia | Lachnospirales | Lachnospiraceae | Agathobacter | Agathobacter sp900546625 | 0.611859 | 0.034937 |
| MGYG000000140_01913 | Firmicutes_A | Clostridia | Lachnospirales | Lachnospiraceae | UMGS1375 | UMGS1375 sp900066615 | 0.611085 | 0.017864 |
| MGYG000000039_01690 | Firmicutes_A | Clostridia | Oscillospirales | Ruminococcaceae | Faecalibacterium | Faecalibacterium prausnitzii_H | 0.61063 | 0.031876 |
| MGYG000000039_00841 | Firmicutes_A | Clostridia | Oscillospirales | Ruminococcaceae | Faecalibacterium | Faecalibacterium prausnitzii_H | 0.61052 | 0.002619 |
| MGYG000001433_03929 | Bacteroidota | Bacteroidia | Bacteroidales | Bacteroidaceae | Bacteroides | Bacteroides salyersiae | 0.609941 | 0.021518 |
| MGYG000002478_01003 | Bacteroidota | Bacteroidia | Bacteroidales | Bacteroidaceae | Phocaeicola | Phocaeicola dorei | 0.609634 | 0.002756 |
| MGYG000000195_02324 | Firmicutes_A | Clostridia | Oscillospirales | Ruminococcaceae | Faecalibacterium | Faecalibacterium prausnitzii_E | 0.608996 | 0.00507 |
| MGYG000000193_00230 | Firmicutes_A | Clostridia | Lachnospirales | Lachnospiraceae | KLE1615 | KLE1615 sp900066985 | 0.608771 | 0.004194 |
| MGYG000001707_00962 | Firmicutes_A | Clostridia | Lachnospirales | Lachnospiraceae | Coprococcus | Coprococcus sp000154245 | 0.608383 | 0.003578 |
| MGYG000000251_01810 | Firmicutes_A | Clostridia | Lachnospirales | Lachnospiraceae | Fusicatenibacter | Fusicatenibacter saccharivorans | 0.607669 | 0.003822 |
| MGYG000000201_00499 | Firmicutes_A | Clostridia | Lachnospirales | Lachnospiraceae | Blautia_A | Blautia_A sp900066145 | 0.607031 | 0.003116 |
| MGYG000002478_01522 | Bacteroidota | Bacteroidia | Bacteroidales | Bacteroidaceae | Phocaeicola | Phocaeicola dorei | 0.606907 | 0.018924 |
| MGYG000001338_00552 | Firmicutes_A | Clostridia | Lachnospirales | Lachnospiraceae | Blautia_A | Blautia_A wexlerae_A | 0.606387 | 0.028263 |
| MGYG000000193_02120 | Firmicutes_A | Clostridia | Lachnospirales | Lachnospiraceae | KLE1615 | KLE1615 sp900066985 | 0.605967 | 0.02183 |
| MGYG000000142_02506 | Firmicutes_A | Clostridia | Lachnospirales | Lachnospiraceae | Blautia_A | Blautia_A massiliensis | 0.604936 | 0.035462 |
| MGYG000000217_00563 | Firmicutes_A | Clostridia | Lachnospirales | Lachnospiraceae | Acetatifactor | Acetatifactor sp900066565 | 0.6032 | 0.045116 |
| MGYG000002312_03039 | Firmicutes_A | Clostridia | Lachnospirales | Lachnospiraceae | Blautia_A | Blautia_A sp000285855 | 0.602751 | 0.021248 |
| MGYG000001346_01528 | Bacteroidota | Bacteroidia | Bacteroidales | Bacteroidaceae | Bacteroides | Bacteroides uniformis | 0.599931 | 0.024322 |
| MGYG000000060_02830 | Firmicutes_A | Clostridia | Lachnospirales | Lachnospiraceae | Lachnospira | Lachnospira eligens_A | 0.599745 | 0.04142 |
| MGYG000000154_02756 | Firmicutes_A | Clostridia | Lachnospirales | Lachnospiraceae | RUG115 | RUG115 sp900066395 | 0.599138 | 0.002486 |
| MGYG000000212_03103 | Firmicutes_A | Clostridia | Lachnospirales | Lachnospiraceae | Blautia_A | Blautia_A obeum | 0.599131 | 0.020595 |
| MGYG000000251_01412 | Firmicutes_A | Clostridia | Lachnospirales | Lachnospiraceae | Fusicatenibacter | Fusicatenibacter saccharivorans | 0.599029 | 0.001505 |
| MGYG000001707_01370 | Firmicutes_A | Clostridia | Lachnospirales | Lachnospiraceae | Coprococcus | Coprococcus sp000154245 | 0.598495 | 0.022676 |
| MGYG000002492_00017 | Firmicutes_A | Clostridia | Lachnospirales | Lachnospiraceae | Agathobacter | Agathobacter rectalis | 0.598081 | 0.011463 |
| MGYG000000200_01983 | Firmicutes_A | Clostridia | Lachnospirales | Lachnospiraceae | Blautia_A | Blautia_A sp003471165 | 0.59757 | 0.023344 |
| MGYG000000054_02348 | Bacteroidota | Bacteroidia | Bacteroidales | Bacteroidaceae | Bacteroides | Bacteroides acidifaciens | 0.596558 | 0.017586 |
| MGYG000000251_02500 | Firmicutes_A | Clostridia | Lachnospirales | Lachnospiraceae | Fusicatenibacter | Fusicatenibacter saccharivorans | 0.596473 | 0.002386 |
| MGYG000002478_03241 | Bacteroidota | Bacteroidia | Bacteroidales | Bacteroidaceae | Phocaeicola | Phocaeicola dorei | 0.594802 | 0.011465 |
| MGYG000000193_02122 | Firmicutes_A | Clostridia | Lachnospirales | Lachnospiraceae | KLE1615 | KLE1615 sp900066985 | 0.593785 | 0.004984 |
| MGYG000002040_00859 | Firmicutes_A | Clostridia | Oscillospirales | Ruminococcaceae | Faecalibacterium | Faecalibacterium sp900758465 | 0.593679 | 0.027093 |
| MGYG000002478_00015 | Bacteroidota | Bacteroidia | Bacteroidales | Bacteroidaceae | Phocaeicola | Phocaeicola dorei | 0.593231 | 0.008201 |
| MGYG000000251_00623 | Firmicutes_A | Clostridia | Lachnospirales | Lachnospiraceae | Fusicatenibacter | Fusicatenibacter saccharivorans | 0.591424 | 0.008496 |
| MGYG000000002_00285 | Firmicutes_A | Clostridia | Lachnospirales | Lachnospiraceae | Blautia_A | Blautia_A faecis | 0.588455 | 0.025054 |
| MGYG000000251_00324 | Firmicutes_A | Clostridia | Lachnospirales | Lachnospiraceae | Fusicatenibacter | Fusicatenibacter saccharivorans | 0.58786 | 0.030862 |
| MGYG000000002_01955 | Firmicutes_A | Clostridia | Lachnospirales | Lachnospiraceae | Blautia_A | Blautia_A faecis | 0.587674 | 0.013448 |
| MGYG000000193_01428 | Firmicutes_A | Clostridia | Lachnospirales | Lachnospiraceae | KLE1615 | KLE1615 sp900066985 | 0.587004 | 0.035086 |
| MGYG000000193_01326 | Firmicutes_A | Clostridia | Lachnospirales | Lachnospiraceae | KLE1615 | KLE1615 sp900066985 | 0.586545 | 0.011524 |
| MGYG000002272_00718 | Firmicutes_A | Clostridia | Oscillospirales | Ruminococcaceae | Faecalibacterium | Faecalibacterium prausnitzii_D | 0.58585 | 0.002373 |
| MGYG000003937_01263 | Firmicutes_A | Clostridia | Oscillospirales | Ruminococcaceae | Gemmiger | Gemmiger qucibialis | 0.585768 | 0.001406 |

**Table S6** | **Underrepresented microbial proteins in the low-risk group.**

| **Protein ID** | **Phylum** | **Class** | **Order** | **Family** | **Genus** | **Species** | **log_2_FC** | **FDR** |
| --- | --- | --- | --- | --- | --- | --- | --- | --- |
| MGYG000003694_02431 | Firmicutes_A | Clostridia | Lachnospirales | Lachnospiraceae | Agathobacter | Agathobacter faecis | -1.37267 | 1.39E-07 |
| MGYG000003694_00799 | Firmicutes_A | Clostridia | Lachnospirales | Lachnospiraceae | Agathobacter | Agathobacter faecis | -1.36642 | 5.11E-05 |
| MGYG000003499_00032 | Verrucomicrobiota | Kiritimatiellae | RFP12 | UBA1067 | W1P29-020 |  | -1.34052 | 4.73E-06 |
| MGYG000000187_02028 | Firmicutes_A | Clostridia | Lachnospirales | Lachnospiraceae | Lachnospira | Lachnospira sp003537285 | -1.33634 | 1.56E-05 |
| MGYG000002506_01169 | Proteobacteria | Gammaproteobacteria | Enterobacterales | Enterobacteriaceae | Escherichia | Escherichia coli_D | -1.331 | 6.03E-05 |
| MGYG000002284_00667 | Firmicutes_C | Negativicutes | Selenomonadales | Selenomonadaceae | Megamonas | Megamonas funiformis | -1.29018 | 0.00472 |
| MGYG000002454_00606 | Verrucomicrobiota | Verrucomicrobiae | Verrucomicrobiales | Akkermansiaceae | Akkermansia | Akkermansia muciniphila | -1.28654 | 0.000324 |
| MGYG000003504_00073 | Firmicutes_A | Clostridia_A | Christensenellales | CAG-314 | CAG-1435 | CAG-1435 sp003537755 | -1.28006 | 7.91E-05 |
| MGYG000002272_00118 | Firmicutes_A | Clostridia | Oscillospirales | Ruminococcaceae | Faecalibacterium | Faecalibacterium prausnitzii_D | -1.26115 | 0.000367 |
| MGYG000001632_02095 | Firmicutes_A | Clostridia | Oscillospirales | Oscillospiraceae | CAG-170 |  | -1.25144 | 0.000738 |
| MGYG000000213_02770 | Firmicutes_A | Clostridia | Lachnospirales | Lachnospiraceae | Blautia_A | Blautia_A sp003477525 | -1.19636 | 0.002402 |
| MGYG000004479_01801 | Bacteroidota | Bacteroidia | Bacteroidales | Bacteroidaceae | Phocaeicola | Phocaeicola mediterraneensis | -1.18404 | 7.84E-06 |
| MGYG000002506_02347 | Proteobacteria | Gammaproteobacteria | Enterobacterales | Enterobacteriaceae | Escherichia | Escherichia coli_D | -1.17865 | 6.71E-05 |
| MGYG000000062_02475 | Firmicutes_A | Clostridia | Peptostreptococcales | Peptostreptococcaceae | Intestinibacter | Intestinibacter bartlettii | -1.17447 | 0.001927 |
| MGYG000000262_01197 | Firmicutes_A | Clostridia | Lachnospirales | Lachnospiraceae | Anaerobutyricum | Anaerobutyricum hallii | -1.16196 | 3.88E-06 |
| MGYG000002445_01920 | Firmicutes_A | Clostridia | Lachnospirales | Lachnospiraceae | Clostridium_Q | Clostridium_Q sp003024715 | -1.15321 | 6.13E-05 |
| MGYG000003694_03279 | Firmicutes_A | Clostridia | Lachnospirales | Lachnospiraceae | Agathobacter | Agathobacter faecis | -1.1469 | 0.003662 |
| MGYG000002080_00530 | Bacteroidota | Bacteroidia | Bacteroidales | Bacteroidaceae | Prevotella | Prevotella sp900544825 | -1.14197 | 0.000323 |
| MGYG000004717_01612 | Firmicutes_C | Negativicutes | Selenomonadales | Selenomonadaceae | Megamonas | Megamonas hypermegale | -1.12808 | 3.02E-05 |
| MGYG000000062_02187 | Firmicutes_A | Clostridia | Peptostreptococcales | Peptostreptococcaceae | Intestinibacter | Intestinibacter bartlettii | -1.11663 | 5.44E-06 |
| MGYG000003984_00726 | Firmicutes_A | Clostridia | Lachnospirales | Lachnospiraceae | Mediterraneibacter | Mediterraneibacter sp900752395 | -1.11495 | 8.14E-06 |
| MGYG000002295_00987 | Firmicutes_A | Clostridia | Oscillospirales | Ruminococcaceae | Ruminococcus_C | Ruminococcus_C callidus | -1.10367 | 0.00606 |
| MGYG000001338_00134 | Firmicutes_A | Clostridia | Lachnospirales | Lachnospiraceae | Blautia_A | Blautia_A wexlerae_A | -1.0927 | 3.84E-06 |
| MGYG000002753_00835 | Firmicutes_A | Clostridia | Oscillospirales | Oscillospiraceae | CAG-103 |  | -1.08844 | 0.002103 |
| MGYG000002143_00904 | Firmicutes_A | Clostridia | Oscillospirales | Oscillospiraceae | CAG-83 | CAG-83 sp900545585 | -1.08278 | 9.86E-05 |
| MGYG000000216_00964 | Firmicutes_A | Clostridia | Lachnospirales | Lachnospiraceae | Blautia_A | Blautia_A sp003474435 | -1.07985 | 1.60E-06 |
| MGYG000000102_00936 | Firmicutes_A | Clostridia | Peptostreptococcales | Peptostreptococcaceae | Terrisporobacter | Terrisporobacter sp902363255 | -1.07499 | 9.77E-05 |
| MGYG000002478_03718 | Bacteroidota | Bacteroidia | Bacteroidales | Bacteroidaceae | Phocaeicola | Phocaeicola dorei | -1.05735 | 8.10E-05 |
| MGYG000000696_01938 | Bacteroidota | Bacteroidia | Bacteroidales | Tannerellaceae | Parabacteroides | Parabacteroides sp900549585 | -1.05581 | 5.39E-05 |
| MGYG000000039_01612 | Firmicutes_A | Clostridia | Oscillospirales | Ruminococcaceae | Faecalibacterium | Faecalibacterium prausnitzii_H | -1.0482 | 0.000144 |
| MGYG000003891_01058 | Firmicutes_A | Clostridia_A | Christensenellales | CAG-74 | UBA11524 | UBA11524 sp000437595 | -1.03717 | 0.001731 |
| MGYG000000102_03145 | Firmicutes_A | Clostridia | Peptostreptococcales | Peptostreptococcaceae | Terrisporobacter | Terrisporobacter sp902363255 | -1.03481 | 0.000645 |
| MGYG000000142_00842 | Firmicutes_A | Clostridia | Lachnospirales | Lachnospiraceae | Blautia_A | Blautia_A massiliensis | -1.03416 | 1.50E-08 |
| MGYG000003891_00627 | Firmicutes_A | Clostridia_A | Christensenellales | CAG-74 | UBA11524 | UBA11524 sp000437595 | -1.03398 | 0.000952 |
| MGYG000002506_01701 | Proteobacteria | Gammaproteobacteria | Enterobacterales | Enterobacteriaceae | Escherichia | Escherichia coli_D | -1.03302 | 0.001133 |
| MGYG000002136_00898 | Firmicutes_A | Clostridia | Lachnospirales | Lachnospiraceae | UC5-1-2E3 | UC5-1-2E3 sp001304875 | -1.02426 | 0.000118 |
| MGYG000001202_01239 | Firmicutes_A | Clostridia | Oscillospirales | Ruminococcaceae | Ruminococcus_D | Ruminococcus_D sp900539095 | -1.02158 | 0.000134 |
| MGYG000001306_01102 | Bacteroidota | Bacteroidia | Bacteroidales | Bacteroidaceae | Phocaeicola | Phocaeicola coprocola | -1.02078 | 3.53E-05 |
| MGYG000001202_01670 | Firmicutes_A | Clostridia | Oscillospirales | Ruminococcaceae | Ruminococcus_D | Ruminococcus_D sp900539095 | -1.0165 | 4.91E-05 |
| MGYG000001202_00652 | Firmicutes_A | Clostridia | Oscillospirales | Ruminococcaceae | Ruminococcus_D | Ruminococcus_D sp900539095 | -1.01595 | 0.00259 |
| MGYG000002272_00198 | Firmicutes_A | Clostridia | Oscillospirales | Ruminococcaceae | Faecalibacterium | Faecalibacterium prausnitzii_D | -1.01472 | 0.000656 |
| MGYG000003891_01710 | Firmicutes_A | Clostridia_A | Christensenellales | CAG-74 | UBA11524 | UBA11524 sp000437595 | -1.00392 | 0.002001 |
| MGYG000000271_01499 | Firmicutes_A | Clostridia | Lachnospirales | Lachnospiraceae | Roseburia | Roseburia sp900552665 | -1.00118 | 0.002851 |
| MGYG000000084_01085 | Firmicutes_A | Clostridia | Oscillospirales | Ruminococcaceae | Gemmiger | Gemmiger formicilis | -0.98249 | 3.71E-05 |
| MGYG000002545_01744 | Firmicutes_A | Clostridia | Oscillospirales | Ruminococcaceae | Faecalibacterium | Faecalibacterium prausnitzii_G | -0.97945 | 5.33E-06 |
| MGYG000002993_01040 | Firmicutes_A | Clostridia | Oscillospirales | Oscillospiraceae | CAG-170 | CAG-170 sp000432135 | -0.97698 | 0.003349 |
| MGYG000000062_02843 | Firmicutes_A | Clostridia | Peptostreptococcales | Peptostreptococcaceae | Intestinibacter | Intestinibacter bartlettii | -0.97569 | 1.58E-06 |
| MGYG000003937_00035 | Firmicutes_A | Clostridia | Oscillospirales | Ruminococcaceae | Gemmiger | Gemmiger qucibialis | -0.97513 | 6.54E-05 |
| MGYG000002715_01848 | Firmicutes_A | Clostridia | Oscillospirales | Oscillospiraceae | ER4 | ER4 sp900317525 | -0.97206 | 0.002404 |
| MGYG000001632_01474 | Firmicutes_A | Clostridia | Oscillospirales | Oscillospiraceae | CAG-170 |  | -0.97049 | 4.85E-06 |
| MGYG000000231_02144 | Firmicutes_A | Clostridia | Lachnospirales | Lachnospiraceae | Coprococcus | Coprococcus eutactus_A | -0.96582 | 0.001056 |
| MGYG000000140_00955 | Firmicutes_A | Clostridia | Lachnospirales | Lachnospiraceae | UMGS1375 | UMGS1375 sp900066615 | -0.95832 | 0.000202 |
| MGYG000004276_00787 | Firmicutes_A | Clostridia | Oscillospirales | Oscillospiraceae | ER4 | ER4 sp900550165 | -0.9583 | 9.53E-06 |
| MGYG000000062_02395 | Firmicutes_A | Clostridia | Peptostreptococcales | Peptostreptococcaceae | Intestinibacter | Intestinibacter bartlettii | -0.95736 | 0.002299 |
| MGYG000001627_02240 | Firmicutes_A | Clostridia | Oscillospirales | Ruminococcaceae | Gemmiger | Gemmiger sp900539695 | -0.95592 | 1.22E-07 |
| MGYG000000176_01919 | Firmicutes_A | Clostridia | Lachnospirales | Lachnospiraceae | Muricomes | Muricomes oroticus | -0.94668 | 0.002084 |
| MGYG000002992_01053 | Firmicutes_A | Clostridia | Lachnospirales | Lachnospiraceae | Dorea_A | Dorea_A sp900550865 | -0.9398 | 1.68E-05 |
| MGYG000003899_00266 | Firmicutes_A | Clostridia | Oscillospirales | Ruminococcaceae | Faecalibacterium | Faecalibacterium sp900539945 | -0.93825 | 0.00019 |
| MGYG000002272_00222 | Firmicutes_A | Clostridia | Oscillospirales | Ruminococcaceae | Faecalibacterium | Faecalibacterium prausnitzii_D | -0.93457 | 0.000189 |
| MGYG000002478_02240 | Bacteroidota | Bacteroidia | Bacteroidales | Bacteroidaceae | Phocaeicola | Phocaeicola dorei | -0.93322 | 1.32E-05 |
| MGYG000000553_01111 | Bacteroidota | Bacteroidia | Bacteroidales | Bacteroidaceae | Prevotella | Prevotella sp900548535 | -0.9313 | 0.000284 |
| MGYG000000233_01880 | Firmicutes_A | Clostridia | Lachnospirales | Lachnospiraceae | CAG-81 | CAG-81 sp900066785 | -0.92812 | 0.00218 |
| MGYG000002993_01959 | Firmicutes_A | Clostridia | Oscillospirales | Oscillospiraceae | CAG-170 | CAG-170 sp000432135 | -0.92654 | 0.00067 |
| MGYG000001202_02008 | Firmicutes_A | Clostridia | Oscillospirales | Ruminococcaceae | Ruminococcus_D | Ruminococcus_D sp900539095 | -0.92602 | 0.000209 |
| MGYG000002271_01479 | Firmicutes_A | Clostridia | Lachnospirales | Lachnospiraceae | GCA-900066135 | GCA-900066135 sp900066135 | -0.92501 | 4.17E-05 |
| MGYG000000245_03069 | Firmicutes_A | Clostridia | Lachnospirales | Lachnospiraceae | Roseburia | Roseburia sp003470905 | -0.92489 | 0.005529 |
| MGYG000000242_02251 | Firmicutes_A | Clostridia | Lachnospirales | Lachnospiraceae | Enterocloster | Enterocloster sp000431375 | -0.92308 | 1.28E-06 |
| MGYG000002506_00283 | Proteobacteria | Gammaproteobacteria | Enterobacterales | Enterobacteriaceae | Escherichia | Escherichia coli_D | -0.91974 | 0.000708 |
| MGYG000003694_02428 | Firmicutes_A | Clostridia | Lachnospirales | Lachnospiraceae | Agathobacter | Agathobacter faecis | -0.91895 | 0.000362 |
| MGYG000002271_01058 | Firmicutes_A | Clostridia | Lachnospirales | Lachnospiraceae | GCA-900066135 | GCA-900066135 sp900066135 | -0.91837 | 3.92E-06 |
| MGYG000002156_01162 | Firmicutes_A | Clostridia | Oscillospirales | Oscillospiraceae | CAG-83 | CAG-83 sp900550585 | -0.91414 | 8.18E-06 |
| MGYG000000745_01455 | Firmicutes_A | Clostridia_A | Christensenellales | CAG-74 |  |  | -0.91305 | 3.83E-05 |
| MGYG000000271_02750 | Firmicutes_A | Clostridia | Lachnospirales | Lachnospiraceae | Roseburia | Roseburia sp900552665 | -0.91119 | 0.000347 |
| MGYG000004852_00241 | Firmicutes_A | Clostridia | Clostridiales | Clostridiaceae | Clostridium |  | -0.90686 | 0.000196 |
| MGYG000004804_01388 | Firmicutes_C | Negativicutes | Veillonellales | Dialisteraceae | Dialister | Dialister sp900759415 | -0.90386 | 0.003322 |
| MGYG000000334_02085 | Firmicutes_A | Clostridia | Oscillospirales | Oscillospiraceae | UBA5446 |  | -0.90289 | 0.000715 |
| MGYG000000062_00872 | Firmicutes_A | Clostridia | Peptostreptococcales | Peptostreptococcaceae | Intestinibacter | Intestinibacter bartlettii | -0.90257 | 4.47E-05 |
| MGYG000002641_00844 | Firmicutes_A | Clostridia | Oscillospirales | Ruminococcaceae | Faecalibacterium | Faecalibacterium sp003449675 | -0.90088 | 0.000205 |
| MGYG000003891_02516 | Firmicutes_A | Clostridia_A | Christensenellales | CAG-74 | UBA11524 | UBA11524 sp000437595 | -0.89843 | 0.000738 |
| MGYG000003891_00992 | Firmicutes_A | Clostridia_A | Christensenellales | CAG-74 | UBA11524 | UBA11524 sp000437595 | -0.89713 | 9.91E-05 |
| MGYG000000250_02940 | Firmicutes_A | Clostridia | Lachnospirales | Lachnospiraceae | TF01-11 | TF01-11 sp001414325 | -0.89569 | 0.002111 |
| MGYG000003891_00316 | Firmicutes_A | Clostridia_A | Christensenellales | CAG-74 | UBA11524 | UBA11524 sp000437595 | -0.88208 | 0.002326 |
| MGYG000001878_01040 | Bacteroidota | Bacteroidia | Bacteroidales | Tannerellaceae | Parabacteroides | Parabacteroides sp900548175 | -0.88194 | 8.00E-07 |
| MGYG000003937_00266 | Firmicutes_A | Clostridia | Oscillospirales | Ruminococcaceae | Gemmiger | Gemmiger qucibialis | -0.88149 | 0.001208 |
| MGYG000001338_02538 | Firmicutes_A | Clostridia | Lachnospirales | Lachnospiraceae | Blautia_A | Blautia_A wexlerae_A | -0.87671 | 0.000426 |
| MGYG000000233_00343 | Firmicutes_A | Clostridia | Lachnospirales | Lachnospiraceae | CAG-81 | CAG-81 sp900066785 | -0.87446 | 0.000341 |
| MGYG000000142_01260 | Firmicutes_A | Clostridia | Lachnospirales | Lachnospiraceae | Blautia_A | Blautia_A massiliensis | -0.87399 | 0.004359 |
| MGYG000000084_01005 | Firmicutes_A | Clostridia | Oscillospirales | Ruminococcaceae | Gemmiger | Gemmiger formicilis | -0.86741 | 5.16E-06 |
| MGYG000003937_01120 | Firmicutes_A | Clostridia | Oscillospirales | Ruminococcaceae | Gemmiger | Gemmiger qucibialis | -0.86605 | 0.000259 |
| MGYG000002272_01917 | Firmicutes_A | Clostridia | Oscillospirales | Ruminococcaceae | Faecalibacterium | Faecalibacterium prausnitzii_D | -0.86542 | 0.000621 |
| MGYG000004296_00587 | Firmicutes_A | Clostridia | Lachnospirales | Lachnospiraceae | Ruminococcus_A | Ruminococcus_A faecicola | -0.8617 | 0.000514 |
| MGYG000000251_02776 | Firmicutes_A | Clostridia | Lachnospirales | Lachnospiraceae | Fusicatenibacter | Fusicatenibacter saccharivorans | -0.85834 | 0.00269 |
| MGYG000000195_03023 | Firmicutes_A | Clostridia | Oscillospirales | Ruminococcaceae | Faecalibacterium | Faecalibacterium prausnitzii_E | -0.85604 | 3.65E-06 |
| MGYG000003694_02446 | Firmicutes_A | Clostridia | Lachnospirales | Lachnospiraceae | Agathobacter | Agathobacter faecis | -0.85459 | 0.000771 |
| MGYG000002040_01760 | Firmicutes_A | Clostridia | Oscillospirales | Ruminococcaceae | Faecalibacterium | Faecalibacterium sp900758465 | -0.85427 | 9.44E-06 |
| MGYG000002258_00535 | Firmicutes_A | Clostridia | Oscillospirales | Acutalibacteraceae | UBA1081 | UBA1081 sp900543395 | -0.85101 | 1.24E-05 |
| MGYG000004185_01144 | Bacteroidota | Bacteroidia | Bacteroidales | Bacteroidaceae | Bacteroides | Bacteroides sp900553815 | -0.85037 | 1.55E-05 |
| MGYG000002561_00872 | Bacteroidota | Bacteroidia | Bacteroidales | Bacteroidaceae | Bacteroides | Bacteroides sp902388495 | -0.84633 | 0.000131 |
| MGYG000002295_00130 | Firmicutes_A | Clostridia | Oscillospirales | Ruminococcaceae | Ruminococcus_C | Ruminococcus_C callidus | -0.84361 | 0.002005 |
| MGYG000003891_01438 | Firmicutes_A | Clostridia_A | Christensenellales | CAG-74 | UBA11524 | UBA11524 sp000437595 | -0.84346 | 1.69E-05 |
| MGYG000003452_00168 | Actinobacteriota | Actinomycetia | Actinomycetales | Bifidobacteriaceae | Bifidobacterium | Bifidobacterium ruminantium | -0.82946 | 0.007254 |
| MGYG000000231_02468 | Firmicutes_A | Clostridia | Lachnospirales | Lachnospiraceae | Coprococcus | Coprococcus eutactus_A | -0.82296 | 0.000196 |
| MGYG000002272_02661 | Firmicutes_A | Clostridia | Oscillospirales | Ruminococcaceae | Faecalibacterium | Faecalibacterium prausnitzii_D | -0.82259 | 5.80E-05 |
| MGYG000002993_00635 | Firmicutes_A | Clostridia | Oscillospirales | Oscillospiraceae | CAG-170 | CAG-170 sp000432135 | -0.82247 | 0.000818 |
| MGYG000001315_01699 | Firmicutes_A | Clostridia | Lachnospirales | Lachnospiraceae | Bariatricus | Bariatricus comes | -0.8215 | 0.000332 |
| MGYG000002321_02794 | Firmicutes_A | Clostridia | Oscillospirales | Ruminococcaceae | Ruminococcus_D | Ruminococcus_D bicirculans | -0.82141 | 0.001995 |
| MGYG000002545_00121 | Firmicutes_A | Clostridia | Oscillospirales | Ruminococcaceae | Faecalibacterium | Faecalibacterium prausnitzii_G | -0.81949 | 0.000663 |
| MGYG000000977_01740 | Firmicutes_A | Clostridia | Clostridiales | Clostridiaceae | Clostridium | Clostridium sp900539375 | -0.81794 | 0.003912 |
| MGYG000002670_01510 | Firmicutes_A | Clostridia | Lachnospirales | Lachnospiraceae | Agathobacter | Agathobacter sp900546625 | -0.81782 | 0.000668 |
| MGYG000001202_01671 | Firmicutes_A | Clostridia | Oscillospirales | Ruminococcaceae | Ruminococcus_D | Ruminococcus_D sp900539095 | -0.81435 | 5.32E-05 |
| MGYG000000404_00339 | Firmicutes_A | Clostridia | Lachnospirales | Lachnospiraceae | UBA11774 | UBA11774 sp003507655 | -0.81305 | 1.64E-05 |
| MGYG000004789_00661 | Firmicutes | Bacilli | Lactobacillales | Streptococcaceae | Streptococcus | Streptococcus sp001556435 | -0.81279 | 0.000255 |
| MGYG000002293_00071 | Bacteroidota | Bacteroidia | Bacteroidales | Bacteroidaceae | Prevotella | Prevotella sp900557255 | -0.81227 | 0.004806 |
| MGYG000001292_01122 | Actinobacteriota | Actinomycetia | Actinomycetales | Bifidobacteriaceae | Bifidobacterium | Bifidobacterium infantis | -0.81149 | 0.004191 |
| MGYG000001490_00332 | Actinobacteriota | Actinomycetia | Actinomycetales | Bifidobacteriaceae | Bifidobacterium | Bifidobacterium catenulatum | -0.81129 | 0.002329 |
| MGYG000003937_00168 | Firmicutes_A | Clostridia | Oscillospirales | Ruminococcaceae | Gemmiger | Gemmiger qucibialis | -0.81071 | 0.002432 |
| MGYG000000062_02185 | Firmicutes_A | Clostridia | Peptostreptococcales | Peptostreptococcaceae | Intestinibacter | Intestinibacter bartlettii | -0.80954 | 0.004083 |
| MGYG000003937_00809 | Firmicutes_A | Clostridia | Oscillospirales | Ruminococcaceae | Gemmiger | Gemmiger qucibialis | -0.80924 | 1.30E-06 |
| MGYG000000062_00278 | Firmicutes_A | Clostridia | Peptostreptococcales | Peptostreptococcaceae | Intestinibacter | Intestinibacter bartlettii | -0.80664 | 0.006756 |
| MGYG000000200_02411 | Firmicutes_A | Clostridia | Lachnospirales | Lachnospiraceae | Blautia_A | Blautia_A sp003471165 | -0.80022 | 0.002353 |
| MGYG000002033_00995 | Bacteroidota | Bacteroidia | Bacteroidales | Tannerellaceae | Parabacteroides | Parabacteroides massiliensis | -0.79862 | 3.08E-07 |
| MGYG000002321_00090 | Firmicutes_A | Clostridia | Oscillospirales | Ruminococcaceae | Ruminococcus_D | Ruminococcus_D bicirculans | -0.79817 | 0.002345 |
| MGYG000002926_00638 | Firmicutes_A | Clostridia | Oscillospirales | Oscillospiraceae | CAG-83 | CAG-83 sp000431575 | -0.79801 | 0.000645 |
| MGYG000001202_01087 | Firmicutes_A | Clostridia | Oscillospirales | Ruminococcaceae | Ruminococcus_D | Ruminococcus_D sp900539095 | -0.79683 | 0.002564 |
| MGYG000000022_02023 | Firmicutes_A | Clostridia | Oscillospirales | Ruminococcaceae | Faecalibacterium | Faecalibacterium prausnitzii_C | -0.79619 | 0.002036 |
| MGYG000002592_00384 | Bacteroidota | Bacteroidia | Bacteroidales | Rikenellaceae | Alistipes | Alistipes sp902388705 | -0.79226 | 0.001254 |
| MGYG000003891_01657 | Firmicutes_A | Clostridia_A | Christensenellales | CAG-74 | UBA11524 | UBA11524 sp000437595 | -0.78609 | 0.001771 |
| MGYG000000553_00858 | Bacteroidota | Bacteroidia | Bacteroidales | Bacteroidaceae | Prevotella | Prevotella sp900548535 | -0.78561 | 0.00398 |
| MGYG000000250_02321 | Firmicutes_A | Clostridia | Lachnospirales | Lachnospiraceae | TF01-11 | TF01-11 sp001414325 | -0.77565 | 0.00035 |
| MGYG000002993_00656 | Firmicutes_A | Clostridia | Oscillospirales | Oscillospiraceae | CAG-170 | CAG-170 sp000432135 | -0.77485 | 0.000137 |
| MGYG000002926_00765 | Firmicutes_A | Clostridia | Oscillospirales | Oscillospiraceae | CAG-83 | CAG-83 sp000431575 | -0.77379 | 0.001072 |
| MGYG000004133_00128 | Firmicutes_A | Clostridia | HGM11514 | HGM11514 |  |  | -0.77141 | 0.005427 |
| MGYG000003694_02997 | Firmicutes_A | Clostridia | Lachnospirales | Lachnospiraceae | Agathobacter | Agathobacter faecis | -0.76752 | 0.001573 |
| MGYG000002992_00972 | Firmicutes_A | Clostridia | Lachnospirales | Lachnospiraceae | Dorea_A | Dorea_A sp900550865 | -0.76578 | 0.000918 |
| MGYG000002478_01737 | Bacteroidota | Bacteroidia | Bacteroidales | Bacteroidaceae | Phocaeicola | Phocaeicola dorei | -0.76555 | 0.002302 |
| MGYG000000084_00473 | Firmicutes_A | Clostridia | Oscillospirales | Ruminococcaceae | Gemmiger | Gemmiger formicilis | -0.75862 | 0.0011 |
| MGYG000000084_01030 | Firmicutes_A | Clostridia | Oscillospirales | Ruminococcaceae | Gemmiger | Gemmiger formicilis | -0.75686 | 0.000187 |
| MGYG000001756_00595 | Firmicutes_A | Clostridia | Oscillospirales | Ruminococcaceae | UBA1394 | UBA1394 sp900538575 | -0.75677 | 0.006391 |
| MGYG000003694_00087 | Firmicutes_A | Clostridia | Lachnospirales | Lachnospiraceae | Agathobacter | Agathobacter faecis | -0.75468 | 0.00019 |
| MGYG000002992_01430 | Firmicutes_A | Clostridia | Lachnospirales | Lachnospiraceae | Dorea_A | Dorea_A sp900550865 | -0.75302 | 0.000607 |
| MGYG000002274_01133 | Firmicutes_A | Clostridia | Oscillospirales | Ruminococcaceae | Faecalibacterium | Faecalibacterium prausnitzii_I | -0.75187 | 0.002736 |
| MGYG000002321_00325 | Firmicutes_A | Clostridia | Oscillospirales | Ruminococcaceae | Ruminococcus_D | Ruminococcus_D bicirculans | -0.75112 | 0.004496 |
| MGYG000001238_00443 | Firmicutes_A | Clostridia | Oscillospirales | Oscillospiraceae | Evtepia | Evtepia sp900758955 | -0.74913 | 8.10E-06 |
| MGYG000000054_03875 | Bacteroidota | Bacteroidia | Bacteroidales | Bacteroidaceae | Bacteroides | Bacteroides acidifaciens | -0.74872 | 0.002879 |
| MGYG000002438_02892 | Bacteroidota | Bacteroidia | Bacteroidales | Tannerellaceae | Parabacteroides | Parabacteroides distasonis | -0.74344 | 3.72E-05 |
| MGYG000003937_00291 | Firmicutes_A | Clostridia | Oscillospirales | Ruminococcaceae | Gemmiger | Gemmiger qucibialis | -0.74307 | 0.000138 |
| MGYG000000084_02177 | Firmicutes_A | Clostridia | Oscillospirales | Ruminococcaceae | Gemmiger | Gemmiger formicilis | -0.74208 | 0.000263 |
| MGYG000002506_03866 | Proteobacteria | Gammaproteobacteria | Enterobacterales | Enterobacteriaceae | Escherichia | Escherichia coli_D | -0.74148 | 0.003016 |
| MGYG000000193_00831 | Firmicutes_A | Clostridia | Lachnospirales | Lachnospiraceae | KLE1615 | KLE1615 sp900066985 | -0.74124 | 0.000278 |
| MGYG000001793_01003 | Firmicutes_A | Clostridia | Lachnospirales | Lachnospiraceae | CAG-632 | CAG-632 sp002406015 | -0.74015 | 0.002433 |
| MGYG000004471_01860 | Firmicutes_A | Clostridia | Oscillospirales | Ruminococcaceae | UBA1394 | UBA1394 sp900554975 | -0.73932 | 8.00E-05 |
| MGYG000000022_01262 | Firmicutes_A | Clostridia | Oscillospirales | Ruminococcaceae | Faecalibacterium | Faecalibacterium prausnitzii_C | -0.73768 | 0.004954 |
| MGYG000003891_01937 | Firmicutes_A | Clostridia_A | Christensenellales | CAG-74 | UBA11524 | UBA11524 sp000437595 | -0.73616 | 0.005419 |
| MGYG000000062_00764 | Firmicutes_A | Clostridia | Peptostreptococcales | Peptostreptococcaceae | Intestinibacter | Intestinibacter bartlettii | -0.7357 | 0.000566 |
| MGYG000002926_00666 | Firmicutes_A | Clostridia | Oscillospirales | Oscillospiraceae | CAG-83 | CAG-83 sp000431575 | -0.73525 | 0.001038 |
| MGYG000001576_01013 | Firmicutes_A | Clostridia | Oscillospirales | Oscillospiraceae | Dysosmobacter | Dysosmobacter sp014297375 | -0.73107 | 6.91E-06 |
| MGYG000001202_01987 | Firmicutes_A | Clostridia | Oscillospirales | Ruminococcaceae | Ruminococcus_D | Ruminococcus_D sp900539095 | -0.73023 | 1.34E-06 |
| MGYG000002478_01478 | Bacteroidota | Bacteroidia | Bacteroidales | Bacteroidaceae | Phocaeicola | Phocaeicola dorei | -0.72996 | 0.002093 |
| MGYG000003891_02248 | Firmicutes_A | Clostridia_A | Christensenellales | CAG-74 | UBA11524 | UBA11524 sp000437595 | -0.72774 | 0.000556 |
| MGYG000000062_02498 | Firmicutes_A | Clostridia | Peptostreptococcales | Peptostreptococcaceae | Intestinibacter | Intestinibacter bartlettii | -0.72681 | 7.57E-07 |
| MGYG000002993_00844 | Firmicutes_A | Clostridia | Oscillospirales | Oscillospiraceae | CAG-170 | CAG-170 sp000432135 | -0.72615 | 0.000773 |
| MGYG000000078_01451 | Firmicutes_A | Clostridia | Lachnospirales | Lachnospiraceae | Lachnospira | Lachnospira rogosae_A | -0.72212 | 0.000427 |
| MGYG000002138_00394 | Firmicutes_A | Clostridia | Oscillospirales | Butyricicoccaceae | Agathobaculum | Agathobaculum sp900555465 | -0.71778 | 0.000459 |
| MGYG000000530_00568 | Firmicutes_A | Clostridia_A | Christensenellales | CAG-138 | PeH17 | PeH17 sp000435055 | -0.71308 | 0.002008 |
| MGYG000003694_01192 | Firmicutes_A | Clostridia | Lachnospirales | Lachnospiraceae | Agathobacter | Agathobacter faecis | -0.7128 | 5.25E-05 |
| MGYG000001292_01866 | Actinobacteriota | Actinomycetia | Actinomycetales | Bifidobacteriaceae | Bifidobacterium | Bifidobacterium infantis | -0.71229 | 7.64E-05 |
| MGYG000002506_02967 | Proteobacteria | Gammaproteobacteria | Enterobacterales | Enterobacteriaceae | Escherichia | Escherichia coli_D | -0.7109 | 0.004646 |
| MGYG000000062_00763 | Firmicutes_A | Clostridia | Peptostreptococcales | Peptostreptococcaceae | Intestinibacter | Intestinibacter bartlettii | -0.70987 | 0.002543 |
| MGYG000001490_00323 | Actinobacteriota | Actinomycetia | Actinomycetales | Bifidobacteriaceae | Bifidobacterium | Bifidobacterium catenulatum | -0.70793 | 0.003826 |
| MGYG000001189_01141 | Firmicutes_A | Clostridia | Lachnospirales | Lachnospiraceae | Blautia_A |  | -0.70712 | 0.002169 |
| MGYG000001302.1_00066 | Bacteroidota | Bacteroidia | Bacteroidales | Rikenellaceae | Alistipes | Alistipes putredinis | -0.70587 | 0.002989 |
| MGYG000000176_02073 | Firmicutes_A | Clostridia | Lachnospirales | Lachnospiraceae | Muricomes | Muricomes oroticus | -0.70408 | 0.00012 |
| MGYG000003891_01569 | Firmicutes_A | Clostridia_A | Christensenellales | CAG-74 | UBA11524 | UBA11524 sp000437595 | -0.70374 | 0.00281 |
| MGYG000000193_03392 | Firmicutes_A | Clostridia | Lachnospirales | Lachnospiraceae | KLE1615 | KLE1615 sp900066985 | -0.70249 | 0.002044 |
| MGYG000000213_01105 | Firmicutes_A | Clostridia | Lachnospirales | Lachnospiraceae | Blautia_A | Blautia_A sp003477525 | -0.70238 | 0.001107 |
| MGYG000003694_00616 | Firmicutes_A | Clostridia | Lachnospirales | Lachnospiraceae | Agathobacter | Agathobacter faecis | -0.70162 | 0.001399 |
| MGYG000001456.1_00428 | Firmicutes_A | Clostridia | Lachnospirales | Lachnospiraceae | Eubacterium_I | Eubacterium_I ramulus | -0.70138 | 0.001402 |
| MGYG000000217_01833 | Firmicutes_A | Clostridia | Lachnospirales | Lachnospiraceae | Acetatifactor | Acetatifactor sp900066565 | -0.70109 | 0.004011 |
| MGYG000004276_01056 | Firmicutes_A | Clostridia | Oscillospirales | Oscillospiraceae | ER4 | ER4 sp900550165 | -0.69998 | 3.80E-05 |
| MGYG000004317_00848 | Firmicutes_A | Clostridia | Lachnospirales | Lachnospiraceae | Lachnospira | Lachnospira sp900316325 | -0.69978 | 1.18E-05 |
| MGYG000004317_00484 | Firmicutes_A | Clostridia | Lachnospirales | Lachnospiraceae | Lachnospira | Lachnospira sp900316325 | -0.69945 | 0.00241 |
| MGYG000001346_02333 | Bacteroidota | Bacteroidia | Bacteroidales | Bacteroidaceae | Bacteroides | Bacteroides uniformis | -0.69519 | 0.001198 |
| MGYG000003899_02233 | Firmicutes_A | Clostridia | Oscillospirales | Ruminococcaceae | Faecalibacterium | Faecalibacterium sp900539945 | -0.69327 | 6.64E-05 |
| MGYG000002143_00199 | Firmicutes_A | Clostridia | Oscillospirales | Oscillospiraceae | CAG-83 | CAG-83 sp900545585 | -0.68999 | 0.002883 |
| MGYG000001632_00765 | Firmicutes_A | Clostridia | Oscillospirales | Oscillospiraceae | CAG-170 |  | -0.68991 | 0.000279 |
| MGYG000001632_00974 | Firmicutes_A | Clostridia | Oscillospirales | Oscillospiraceae | CAG-170 |  | -0.68985 | 0.005482 |
| MGYG000001202_00675 | Firmicutes_A | Clostridia | Oscillospirales | Ruminococcaceae | Ruminococcus_D | Ruminococcus_D sp900539095 | -0.68913 | 0.001792 |
| MGYG000000233_01278 | Firmicutes_A | Clostridia | Lachnospirales | Lachnospiraceae | CAG-81 | CAG-81 sp900066785 | -0.68762 | 0.00492 |
| MGYG000002926_00691 | Firmicutes_A | Clostridia | Oscillospirales | Oscillospiraceae | CAG-83 | CAG-83 sp000431575 | -0.68753 | 0.00322 |
| MGYG000000250_00274 | Firmicutes_A | Clostridia | Lachnospirales | Lachnospiraceae | TF01-11 | TF01-11 sp001414325 | -0.68742 | 0.002324 |
| MGYG000000251_02767 | Firmicutes_A | Clostridia | Lachnospirales | Lachnospiraceae | Fusicatenibacter | Fusicatenibacter saccharivorans | -0.68539 | 0.003329 |
| MGYG000002994_01417 | Firmicutes_C | Negativicutes | Veillonellales | Megasphaeraceae | Megasphaera | Megasphaera sp002319965 | -0.68456 | 0.00715 |
| MGYG000000002_02323 | Firmicutes_A | Clostridia | Lachnospirales | Lachnospiraceae | Blautia_A | Blautia_A faecis | -0.68347 | 1.21E-05 |
| MGYG000000251_01069 | Firmicutes_A | Clostridia | Lachnospirales | Lachnospiraceae | Fusicatenibacter | Fusicatenibacter saccharivorans | -0.68263 | 0.001184 |
| MGYG000002545_01168 | Firmicutes_A | Clostridia | Oscillospirales | Ruminococcaceae | Faecalibacterium | Faecalibacterium prausnitzii_G | -0.68144 | 1.44E-06 |
| MGYG000001433_00188 | Bacteroidota | Bacteroidia | Bacteroidales | Bacteroidaceae | Bacteroides | Bacteroides salyersiae | -0.68133 | 4.59E-05 |
| MGYG000002517_00490 | Firmicutes_A | Clostridia | Lachnospirales | Lachnospiraceae | Roseburia | Roseburia hominis | -0.68001 | 0.003774 |
| MGYG000002993_01282 | Firmicutes_A | Clostridia | Oscillospirales | Oscillospiraceae | CAG-170 | CAG-170 sp000432135 | -0.67932 | 0.0005 |
| MGYG000002753_00606 | Firmicutes_A | Clostridia | Oscillospirales | Oscillospiraceae | CAG-103 |  | -0.67919 | 0.004005 |
| MGYG000000249_00045 | Firmicutes_A | Clostridia | Lachnospirales | Lachnospiraceae | Mediterraneibacter | Mediterraneibacter faecis | -0.67891 | 0.003002 |
| MGYG000004055_01908 | Firmicutes_A | Clostridia | Lachnospirales | Lachnospiraceae | Eubacterium_G | Eubacterium_G sp900548465 | -0.67888 | 3.61E-05 |
| MGYG000003937_01089 | Firmicutes_A | Clostridia | Oscillospirales | Ruminococcaceae | Gemmiger | Gemmiger qucibialis | -0.67687 | 6.19E-06 |
| MGYG000002478_00339 | Bacteroidota | Bacteroidia | Bacteroidales | Bacteroidaceae | Phocaeicola | Phocaeicola dorei | -0.67681 | 0.004792 |
| MGYG000000258_00735 | Firmicutes_A | Clostridia | Oscillospirales | Acutalibacteraceae | Ruminococcus_E | Ruminococcus_E bromii_B | -0.67518 | 0.000805 |
| MGYG000000721_00220 | Firmicutes_A | Clostridia | Lachnospirales | Lachnospiraceae | NC2004 |  | -0.66862 | 0.004076 |
| MGYG000001559_01396 | Firmicutes_C | Negativicutes | Veillonellales | Megasphaeraceae | Anaeroglobus | Anaeroglobus massiliensis | -0.66828 | 0.001512 |
| MGYG000000062_00406 | Firmicutes_A | Clostridia | Peptostreptococcales | Peptostreptococcaceae | Intestinibacter | Intestinibacter bartlettii | -0.66718 | 0.002447 |
| MGYG000003984_00025 | Firmicutes_A | Clostridia | Lachnospirales | Lachnospiraceae | Mediterraneibacter | Mediterraneibacter sp900752395 | -0.66593 | 0.001216 |
| MGYG000001338_02192 | Firmicutes_A | Clostridia | Lachnospirales | Lachnospiraceae | Blautia_A | Blautia_A wexlerae_A | -0.66563 | 0.002605 |
| MGYG000001302.1_00941 | Bacteroidota | Bacteroidia | Bacteroidales | Rikenellaceae | Alistipes | Alistipes putredinis | -0.66555 | 0.002718 |
| MGYG000003937_00298 | Firmicutes_A | Clostridia | Oscillospirales | Ruminococcaceae | Gemmiger | Gemmiger qucibialis | -0.66544 | 6.67E-05 |
| MGYG000001255_01367 | Firmicutes_A | Clostridia | Oscillospirales | Ruminococcaceae | Faecalibacterium | Faecalibacterium prausnitzii_F | -0.66383 | 0.000239 |
| MGYG000000249_02001 | Firmicutes_A | Clostridia | Lachnospirales | Lachnospiraceae | Mediterraneibacter | Mediterraneibacter faecis | -0.66377 | 6.43E-05 |
| MGYG000002528_02635 | Firmicutes_A | Clostridia | Lachnospirales | Lachnospiraceae | Anaerostipes | Anaerostipes hadrus | -0.66362 | 1.61E-05 |
| MGYG000004223_01140 | Firmicutes_A | Clostridia | UMGS1883 | UMGS1883 | UMGS1883 | UMGS1883 sp900763305 | -0.65951 | 0.000977 |
| MGYG000000249_01376 | Firmicutes_A | Clostridia | Lachnospirales | Lachnospiraceae | Mediterraneibacter | Mediterraneibacter faecis | -0.65783 | 1.02E-06 |
| MGYG000000030_00435 | Firmicutes_A | Clostridia | Lachnospirales | Lachnospiraceae | CAG-603 | CAG-603 sp900066105 | -0.65715 | 0.003884 |
| MGYG000000022_00179 | Firmicutes_A | Clostridia | Oscillospirales | Ruminococcaceae | Faecalibacterium | Faecalibacterium prausnitzii_C | -0.65397 | 0.00016 |
| MGYG000000002_01350 | Firmicutes_A | Clostridia | Lachnospirales | Lachnospiraceae | Blautia_A | Blautia_A faecis | -0.65376 | 0.001501 |
| MGYG000002136_00310 | Firmicutes_A | Clostridia | Lachnospirales | Lachnospiraceae | UC5-1-2E3 | UC5-1-2E3 sp001304875 | -0.6534 | 8.42E-05 |
| MGYG000001315_01367 | Firmicutes_A | Clostridia | Lachnospirales | Lachnospiraceae | Bariatricus | Bariatricus comes | -0.64885 | 8.02E-05 |
| MGYG000000249_01610 | Firmicutes_A | Clostridia | Lachnospirales | Lachnospiraceae | Mediterraneibacter | Mediterraneibacter faecis | -0.64759 | 0.005506 |
| MGYG000001255_00146 | Firmicutes_A | Clostridia | Oscillospirales | Ruminococcaceae | Faecalibacterium | Faecalibacterium prausnitzii_F | -0.64749 | 0.000238 |
| MGYG000002992_00002 | Firmicutes_A | Clostridia | Lachnospirales | Lachnospiraceae | Dorea_A | Dorea_A sp900550865 | -0.64585 | 0.001064 |
| MGYG000003869_01397 | Firmicutes_A | Clostridia | Oscillospirales | Oscillospiraceae | Flavonifractor |  | -0.64539 | 2.31E-05 |
| MGYG000002545_01708 | Firmicutes_A | Clostridia | Oscillospirales | Ruminococcaceae | Faecalibacterium | Faecalibacterium prausnitzii_G | -0.64462 | 0.003546 |
| MGYG000003375_01821 | Actinobacteriota | Actinomycetia | Mycobacteriales | Mycobacteriaceae | Corynebacterium | Corynebacterium striatum | -0.64429 | 0.004029 |
| MGYG000002992_01787 | Firmicutes_A | Clostridia | Lachnospirales | Lachnospiraceae | Dorea_A | Dorea_A sp900550865 | -0.6422 | 0.000106 |
| MGYG000000028_01296 | Firmicutes_A | Clostridia | Lachnospirales | Lachnospiraceae | Anaerostipes | Anaerostipes hadrus_A | -0.6403 | 0.00186 |
| MGYG000000084_01055 | Firmicutes_A | Clostridia | Oscillospirales | Ruminococcaceae | Gemmiger | Gemmiger formicilis | -0.63954 | 0.003169 |
| MGYG000000078_01667 | Firmicutes_A | Clostridia | Lachnospirales | Lachnospiraceae | Lachnospira | Lachnospira rogosae_A | -0.6393 | 0.000373 |
| MGYG000004317_01188 | Firmicutes_A | Clostridia | Lachnospirales | Lachnospiraceae | Lachnospira | Lachnospira sp900316325 | -0.63856 | 0.001675 |
| MGYG000002395_01541 | Actinobacteriota | Actinomycetia | Actinomycetales | Bifidobacteriaceae | Bifidobacterium | Bifidobacterium adolescentis | -0.63697 | 0.005194 |
| MGYG000000184_01541 | Firmicutes_A | Clostridia | Lachnospirales | Lachnospiraceae | Blautia_A | Blautia_A luti | -0.63637 | 0.005462 |
| MGYG000000271_00008 | Firmicutes_A | Clostridia | Lachnospirales | Lachnospiraceae | Roseburia | Roseburia sp900552665 | -0.6351 | 4.47E-05 |
| MGYG000004471_00425 | Firmicutes_A | Clostridia | Oscillospirales | Ruminococcaceae | UBA1394 | UBA1394 sp900554975 | -0.63084 | 0.00017 |
| MGYG000003984_00331 | Firmicutes_A | Clostridia | Lachnospirales | Lachnospiraceae | Mediterraneibacter | Mediterraneibacter sp900752395 | -0.6301 | 0.005254 |
| MGYG000003891_01008 | Firmicutes_A | Clostridia_A | Christensenellales | CAG-74 | UBA11524 | UBA11524 sp000437595 | -0.62683 | 0.002611 |
| MGYG000000404_01891 | Firmicutes_A | Clostridia | Lachnospirales | Lachnospiraceae | UBA11774 | UBA11774 sp003507655 | -0.62635 | 0.000458 |
| MGYG000004869_03236 | Firmicutes_A | Clostridia | Lachnospirales | Lachnospiraceae | Lactonifactor | Lactonifactor sp009677585 | -0.62519 | 0.000126 |
| MGYG000002993_00664 | Firmicutes_A | Clostridia | Oscillospirales | Oscillospiraceae | CAG-170 | CAG-170 sp000432135 | -0.62307 | 0.000422 |
| MGYG000003899_02064 | Firmicutes_A | Clostridia | Oscillospirales | Ruminococcaceae | Faecalibacterium | Faecalibacterium sp900539945 | -0.62265 | 0.000113 |
| MGYG000002478_00836 | Bacteroidota | Bacteroidia | Bacteroidales | Bacteroidaceae | Phocaeicola | Phocaeicola dorei | -0.62218 | 0.000549 |
| MGYG000001255_00621 | Firmicutes_A | Clostridia | Oscillospirales | Ruminococcaceae | Faecalibacterium | Faecalibacterium prausnitzii_F | -0.61606 | 0.000484 |
| MGYG000000249_02018 | Firmicutes_A | Clostridia | Lachnospirales | Lachnospiraceae | Mediterraneibacter | Mediterraneibacter faecis | -0.61536 | 7.86E-06 |
| MGYG000002138_00228 | Firmicutes_A | Clostridia | Oscillospirales | Butyricicoccaceae | Agathobaculum | Agathobaculum sp900555465 | -0.6148 | 0.000274 |
| MGYG000000249_00953 | Firmicutes_A | Clostridia | Lachnospirales | Lachnospiraceae | Mediterraneibacter | Mediterraneibacter faecis | -0.61478 | 0.001516 |
| MGYG000002293_01391 | Bacteroidota | Bacteroidia | Bacteroidales | Bacteroidaceae | Prevotella | Prevotella sp900557255 | -0.61418 | 0.000728 |
| MGYG000000262_00030 | Firmicutes_A | Clostridia | Lachnospirales | Lachnospiraceae | Anaerobutyricum | Anaerobutyricum hallii | -0.61375 | 0.00301 |
| MGYG000003694_02682 | Firmicutes_A | Clostridia | Lachnospirales | Lachnospiraceae | Agathobacter | Agathobacter faecis | -0.61315 | 0.005505 |
| MGYG000001456.1_02461 | Firmicutes_A | Clostridia | Lachnospirales | Lachnospiraceae | Eubacterium_I | Eubacterium_I ramulus | -0.61242 | 4.73E-05 |
| MGYG000000249_00677 | Firmicutes_A | Clostridia | Lachnospirales | Lachnospiraceae | Mediterraneibacter | Mediterraneibacter faecis | -0.61077 | 1.69E-06 |
| MGYG000000271_03348 | Firmicutes_A | Clostridia | Lachnospirales | Lachnospiraceae | Roseburia | Roseburia sp900552665 | -0.60952 | 0.000688 |
| MGYG000004479_00416 | Bacteroidota | Bacteroidia | Bacteroidales | Bacteroidaceae | Phocaeicola | Phocaeicola mediterraneensis | -0.60949 | 0.000984 |
| MGYG000003937_01627 | Firmicutes_A | Clostridia | Oscillospirales | Ruminococcaceae | Gemmiger | Gemmiger qucibialis | -0.60735 | 0.005065 |
| MGYG000003899_01863 | Firmicutes_A | Clostridia | Oscillospirales | Ruminococcaceae | Faecalibacterium | Faecalibacterium sp900539945 | -0.60721 | 0.000418 |
| MGYG000002080_01801 | Bacteroidota | Bacteroidia | Bacteroidales | Bacteroidaceae | Prevotella | Prevotella sp900544825 | -0.60664 | 0.006961 |
| MGYG000002570_00055 | Firmicutes_A | Clostridia | Oscillospirales | Acutalibacteraceae | Ruminococcus_E | Ruminococcus_E sp003526955 | -0.60651 | 0.000626 |
| MGYG000001623_02628 | Firmicutes_A | Clostridia | Peptostreptococcales | Peptostreptococcaceae | Terrisporobacter | Terrisporobacter othiniensis | -0.60493 | 0.005157 |
| MGYG000000193_01494 | Firmicutes_A | Clostridia | Lachnospirales | Lachnospiraceae | KLE1615 | KLE1615 sp900066985 | -0.60481 | 0.000329 |
| MGYG000000022_01525 | Firmicutes_A | Clostridia | Oscillospirales | Ruminococcaceae | Faecalibacterium | Faecalibacterium prausnitzii_C | -0.60367 | 0.00463 |
| MGYG000003221_01993 | Bacteroidota | Bacteroidia | Bacteroidales | Bacteroidaceae | OM05-12 | OM05-12 sp900760755 | -0.60349 | 0.00063 |
| MGYG000002478_03805 | Bacteroidota | Bacteroidia | Bacteroidales | Bacteroidaceae | Phocaeicola | Phocaeicola dorei | -0.60288 | 2.95E-05 |
| MGYG000003694_02223 | Firmicutes_A | Clostridia | Lachnospirales | Lachnospiraceae | Agathobacter | Agathobacter faecis | -0.60227 | 0.003916 |
| MGYG000000263_03415 | Firmicutes_A | Clostridia | Lachnospirales | Lachnospiraceae | Blautia_A | Blautia_A sp900066335 | -0.60175 | 5.09E-05 |
| MGYG000002293_01790 | Bacteroidota | Bacteroidia | Bacteroidales | Bacteroidaceae | Prevotella | Prevotella sp900557255 | -0.59538 | 0.001206 |
| MGYG000003694_01384 | Firmicutes_A | Clostridia | Lachnospirales | Lachnospiraceae | Agathobacter | Agathobacter faecis | -0.59162 | 0.000205 |
| MGYG000002993_01286 | Firmicutes_A | Clostridia | Oscillospirales | Oscillospiraceae | CAG-170 | CAG-170 sp000432135 | -0.59084 | 0.003551 |
| MGYG000004691_00620 | Firmicutes_A | Clostridia | Lachnospirales | Lachnospiraceae | UBA9502 | UBA9502 sp900555625 | -0.59064 | 0.000972 |
| MGYG000000242_02358 | Firmicutes_A | Clostridia | Lachnospirales | Lachnospiraceae | Enterocloster | Enterocloster sp000431375 | -0.58883 | 0.005952 |
| MGYG000001632_02055 | Firmicutes_A | Clostridia | Oscillospirales | Oscillospiraceae | CAG-170 |  | -0.58843 | 0.002617 |
| MGYG000001346_00367 | Bacteroidota | Bacteroidia | Bacteroidales | Bacteroidaceae | Bacteroides | Bacteroides uniformis | -0.58766 | 0.000516 |
| MGYG000002993_00499 | Firmicutes_A | Clostridia | Oscillospirales | Oscillospiraceae | CAG-170 | CAG-170 sp000432135 | -0.58691 | 2.38E-05 |
| MGYG000002272_00522 | Firmicutes_A | Clostridia | Oscillospirales | Ruminococcaceae | Faecalibacterium | Faecalibacterium prausnitzii_D | -0.58669 | 0.000485 |
| MGYG000000553_02287 | Bacteroidota | Bacteroidia | Bacteroidales | Bacteroidaceae | Prevotella | Prevotella sp900548535 | -0.58622 | 0.005785 |
| MGYG000002492_01384 | Firmicutes_A | Clostridia | Lachnospirales | Lachnospiraceae | Agathobacter | Agathobacter rectalis | -0.5855 | 0.003389 |

**Table S7** | **Underrepresented vitamin-associated microbial metabolic pathways in the low-risk group.**

| **Vitamin** | **High-risk** | **Low-risk** | **P-value** |
| --- | --- | --- | --- |
| Thiamin (Vitamin B1) mg/day | 1.505[1.112,1.632] | 1.91[1.373,2.057] | 0.012* |
| Riboflavin (Vitamin B2) mg/day | 2.194[1.668,2.613] | 2.717[2.227,3.103] | 0.002** |
| Niacin (Vitamin B3) mg/day | 22.711[16.935,26.471] | 26.578[22.307,31.078] | 0.028* |
| Pyridoxine (Vitamin B6) mg/day | 1.171[0.829,1.348] | 1.268[1.054,1.381] | 0.254 |

**Table S8 | Baseline variables in HFpEF cohort between predicted high-risk and low-risk patients.**

| **Human protein-based model** | | | |
| --- | --- | --- | --- |
| **Variable** | **Low-Risk (n =7)** | **High-Risk (n = 19)** | **p-value** |
| Age (years, mean[median]) | 66[62,71] | 69[66,74] | 0.389 |
| Female (%, n) | 71.4% (5) | 78.9%(15) | 1 |
| BMI (kg/m^2^, mean±SD) | 36.429±5.593 | 31.473±5.461 | 0.07 |
| **Microbial protein-based model** | | | |
| **Variable** | **Low-Risk (n =9)** | **High-Risk (n = 17)** | **p-value** |
| Age (years, mean[median]) | 67[61,71] | 69[66,75] | 0.389 |
| Female (%, n) | 88.9%(9) | 70.6%(12) | 0.380 |
| BMI (kg/m^2^, mean±SD) | 31.706 ± 6.791 | 34.889 ± 5.133 | 0.240 |

Legend: BMI, body mass index.

**Table S9 | Cumulative major cardiovascular events in circulating protein score groups.**

| **Time point** | **Below-median group** | **Above-median group** |
| --- | --- | --- |
| 500 days | 43 | 24 |
| 1000 days | 108 | 54 |
| 1500 days | 172 | 98 |
| 2000 days | 237 | 155 |
| 2500 days | 315 | 208 |
| 3000 days | 377 | 249 |
| Endpoint | 464 | 329 |
